# Sustainable and Multifunctional Natural Macromolecular Polymers for Aqueous Zn Metal Batteries

**DOI:** 10.1002/adma.74525

**Published:** 2026-08-08

**Authors:** Yunuo Shi, Bo Jiang, Jingcheng Zhang, Xiaodong Shao, Peixun Xiong, Lutong Shan, Shengyang Huang, Mengfang Liang, Mengyao Chang, Fangyan Liu, Wenwu Li, Baoyu Xia, Wei Ji, Ho Seok Park

**Affiliations:** ^1^ Center For Innovative Research in Synthetic Chemistry and Resource Utilization College of Chemistry Chemical Engineering and Resource Utilization Northeast Forestry University Harbin P. R. China; ^2^ School of Chemical Engineering Sungkyunkwan University (SKKU) Suwon Gyeonggi‐do Republic of Korea; ^3^ Department of Chemical and Biomolecular Engineering National University of Singapore Singapore; ^4^ State Key Laboratory of Optoelectronic Materials and Technologies Guangdong Province Key Laboratory of Display Material and Technology School of Electronics and Information Technology Sun Yat‐Sen University Guangzhou P. R. China; ^5^ State Key Laboratory of New Textile Materials and Advanced Processing Key Laboratory of Material Chemistry For Energy Conversion and Storage (Ministry of Education) School of Chemistry and Chemical Engineering Huazhong University of Science and Technology Wuhan P. R. China; ^6^ SKKU Institute of Energy Science and Technology (SIEST) Sungkyunkwan University (SKKU) Suwon Gyeonggi‐do Republic of Korea

**Keywords:** cycling stability, multifunctional, natural macromolecular polymers, sustainable, zinc anodes

## Abstract

Aqueous zinc‐ion batteries (AZIBs) have emerged as a highly competitive energy storage system, owing to their inherent safety, ideal capacity, and the abundance of zinc resources. However, the practical applications of AZIBs are significantly hampered by challenges such as uncontrolled dendrite growth, corrosion, and passivation of zinc anodes. Natural macromolecular polymers, leveraging their multifunctionality, sustainability, and multilevel structures featuring superior mechanical strength, demonstrate unique potential in the development of effective strategies for optimizing zinc anodes. This review is the first to systematically analyze, at both the microscopic and mesoscopic levels, the structure‐function relationships between the structural/surface characteristics of natural polymers and their optimization effect on zinc anodes, and elucidates the relevant regulatory mechanisms. Concurrently, the research progress on optimizing zinc anodes with these natural polymers is sorted out and analyzed according to different optimization strategies, further illustrating the interrelationship among the structure/properties of natural polymers, zinc anode optimization strategies, and regulatory mechanisms. Additionally, the review identifies and outlines the crucial challenges faced by natural polymers in terms of optimizing zinc anodes. Ultimately, this review also delivers some prospects for future research directions and innovative engineering strategies focusing on natural polymers to construct ultra‐stable zinc anodes for large‐scale applications.

## Graphical abstract

We comprehensively review the structure‐function relationships and regulatory mechanism of natural macromolecular polymers in stabilizing zinc anodes at the microscopic and mesoscopic scales. The interactions among polymer structures, optimization strategies, and regulatory mechanisms are discussed systematically summarizing recent related research progress, while valuable insights into the current challenges and future research directions are also addressed.

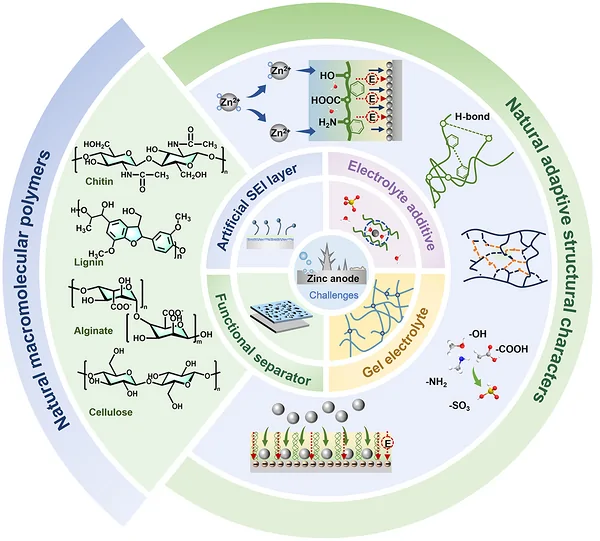

## 1 Introduction

The sustained exploitation and utilization of renewable clean energy sources have raised higher requirements for large‐scale electrochemical energy storage [1, 2, 3, 4]. Lithium‐ion batteries (LIBs), which have long underpinned large‐scale electrochemical energy storage, are increasingly struggling to meet the growing energy storage demands due to safety risks and the rising costs of lithium resources [5, 6, 7, 8]. Aqueous zinc‐ion batteries (AZIBs), with their advantages of high safety, ideal specific capacity and low cost, have emerged as a key alternative to LIBs, illuminating the way forward for large‐scale electrochemical energy storage [9, 10, 11]. However, zinc anodes in AZIBs encounter multiple challenges during cycling, such as uncontrolled dendrite growth, hydrogen evolution corrosion and passivation side reactions, which lead to low coulombic efficiency and unsatisfactory cycling lifespan, seriously hindering the practical application of AZIBs [12, 13, 14, 15]. These challenges and issues essentially stem from the coupled effects of various inherent adverse factors within AZIBs, including the sluggish transport kinetics of zinc ions, uneven distribution of interfacial ion/electric fields, and the unstable mechanical strength and chemical environment at the electrode‐electrolyte interface [16, 17, 18, 19]. To fabricate a zinc anode capable of long‐term cycling stability suitable for large‐scale practical applications, it is necessary to simultaneously address all of the aforementioned adverse factors.

Natural macromolecular polymers (such as chitin, cellulose, lignin, alginate, etc.) typically benefit from their interacting chain or network microstructures, highly tunable surface polarity and hydrophilicity, and outstanding mechanical properties and surface modifiability, as well as remarkable electric field responsiveness and self‐assembly capability, delivering significant multifunctionality in addressing the numerous adverse factors and issues faced by zinc anodes in AZIBs [20, 21, 22, 23]. Specifically, this multifunctionality of natural polymers essentially stems from their naturally formed surfaces rich in polar functional groups, extensive hydrogen‐bonding networks, and inherent hierarchical architectures, whereas synthetic polymers often require sophisticated molecular design and structural engineering strategies to realize comparable multifunctionality. Moreover, natural polymers are readily available, inexpensive, renewable and biodegradable, demonstrating excellent sustainability [24, 25, 26, 27, 28]. It is precisely the multifunctionality brought by these unique structural and surface characteristics and sustainability that endow natural polymers with advantages in stabilizing zinc metal anodes, which has also been confirmed by a large number of studies [29, 30, 31, 32, 33]. However, the structural‐activity correlations between the structural characteristics/surface chemical properties of different natural polymers and the optimization effect on zinc anodes have not been systematically summarized and analyzed so far, and the relevant regulatory laws and multiple deep‐level regulatory mechanisms have not yet been effectively revealed. Establishing these structure‐effect relationships and elucidating the underlying regulatory mechanisms are of great significance and urgency for guiding the development of stable zinc anodes based on natural polymers for large‐scale practical applications.

Herein, we systematically analyzed and established, for the first time from both microscopic and mesoscopic perspectives, the structure‐function relationships between the structural/chemical properties of various natural macromolecular polymers and the optimization effect on the stability of zinc anodes, and meticulously revealed the underlying multiple regulation mechanisms oriented by structural/surface characteristics. These natural polymers can not only promote the desolvation and efficient transport of zinc ions through their abundant surface polar groups and multilevel self‐assembly chain/network microstructures, but their ion selectivity and electric field responsiveness also homogenize the charge distribution and zinc ion flux at the interfaces of zinc anodes (Figure 1). More crucially, the chain/network microstructures stabilized by networks of weak interactions such as hydrogen bonds (H‐bonds) and modified by multiple functional groups enable these natural polymers to exhibit self‐assembly and self‐regulation characteristics in ever‐changing electrochemical environment, thereby dynamically maintaining the mechanical structure and chemical properties of the electrode–electrolyte interface in a stable state. Meanwhile, the representative research progress on optimizing the electrochemical stability of zinc anodes using natural polymers around these important regulatory mechanisms has been categorized, summarized and deeply analyzed according to different optimization strategies in this review, with a view to deepening people's understanding of the interrelationships among the structure/properties of natural polymers, zinc anode optimization strategies, and regulatory mechanisms. Finally, contrapose critical challenges faced by natural macromolecular polymers in optimizing zinc anodes, this review proposes a few perspectives that may provide inspiration for subsequent related research, offering some possible research ideas for successfully constructing natural polymer‐based zinc anodes suitable for large‐scale applications.

**FIGURE 1:**
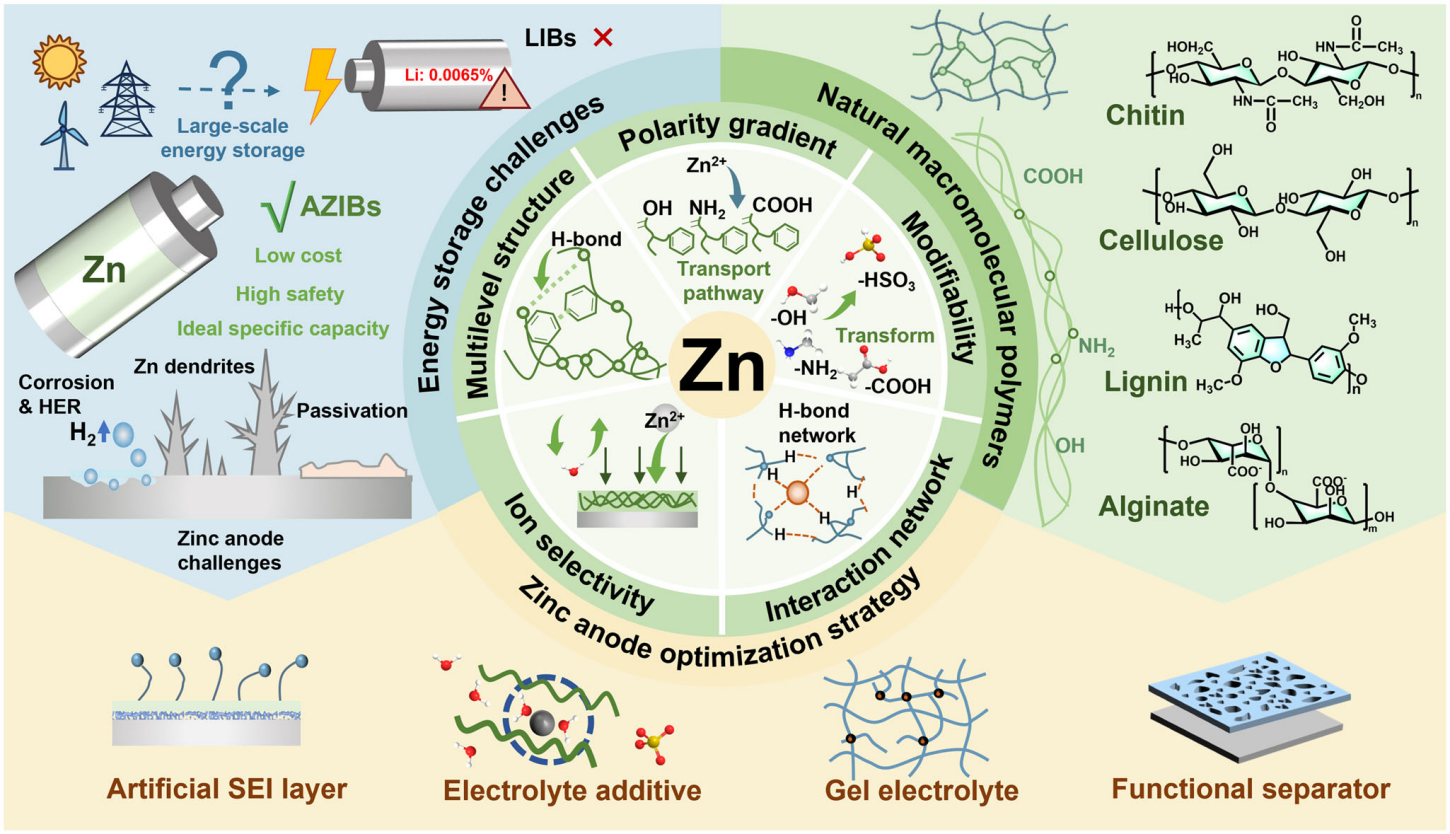
Schematic illustration of the chemical structure of natural macromolecular polymers and their regulatory strategies and related mechanisms in optimizing zinc anodes to address energy storage challenges.

## 2 Stabilizing Zinc Anodes with Natural Macromolecular Polymers: Mechanisms and Merits

AZIBs are conventionally composed of four key components: the cathode, the anode, the separator and the electrolyte. Among these, electrolytes are categorized by their properties into traditional liquid electrolytes, gel electrolytes and solid‐state electrolytes that have garnered significant attention in recent years [34, 35, 36]. Gel or solid‐state electrolytes integrate dual functionalities of ionic conduction and physical barrier, enabling them to function as both electrolyte and separator in batteries, thereby obviating the need for discrete separator elements used in traditional liquid systems. Within AZIBs systems, the reversibility of the deposition/stripping of zinc ions at the anode crucially governs the cycling stability of AZIBs, as represented by the electrochemical reaction (Equation (1)):

(1) $$Zn^{2+}+2e^{-}↔\mathrm{Zn}$$

Significantly, this redox process is accompanied by remarkable volumetric deformation and interfacial reconstruction, which readily provokes interfacial kinetic imbalances and parasitic side reactions, as well as their coupled effects [37, 38]. These intertwined detrimental electrochemical processes constitute the root causes of zinc anode failure, manifested through: (i) zinc corrosion and hydrogen evolution reactions (HER), (ii) irreversible accumulation of passivation layer, (iii) uncontrolled dendritic growth inducing internal short circuits [39, 40]. Consequently, regulating interfacial kinetics and suppressing the aforementioned parasitic reactions constitute the pivotal pathways to optimize zinc anodes, which will inevitably enhance the cycling stability of AZIBs. Currently, the optimization strategies for zinc anodes primarily focus on: constructing artificial solid electrolyte interface (SEI) layers, modulating electrolyte compositions, engineering three‐dimensional electrode architectures, and designing functionalized separators [14, 41, 42, 43]. These approaches synergistically extend cycle life of AZIBs through accelerating Zn^2+^ transport kinetics, homogenizing interfacial electric field distribution, and optimizing desolvation processes. Among the numerous materials applied to improve the electrochemical performance of AZIBs, natural macromolecular polymers exhibit natural superiorities due to their rich active functional groups, environmental friendliness, and cost‐effectiveness. The abundant functional groups, such as hydroxyl (–OH), carboxyl (–COOH), and amino (–NH_2_), in natural polymers not only regulate the deposition behavior of Zn^2+^, but also restructure the electrolyte hydrogen‐bond network, thereby enhancing structural stability of AZIBs. Recently, the improved AZIBs on account of natural macromolecular polymers have been progressively reported, attracting widespread research attention [20, 44, 45, 46].

### 2.1 Failure Mechanism of Zinc Anodes

Generally, the failure of zinc anodes stems from the synergistic coupling of interfacial kinetic imbalance and parasitic side reactions [47, 48, 49, 50, 51]. To be more specific, interfacial side reactions primarily encompass corrosion‐induced HER and surface passivation processes. The former drives continuous gas evolution, while the latter generates electrically insulating byproducts, both inflicting irreversible damage to zinc metal electrodes. During repeated deposition/stripping cycles of zinc ions, the electrode/electrolyte interface exhibits a dynamic instability due to the sluggish transport kinetics of Zn^2+^ and heterogeneous electric field distribution on the surface of zinc electrodes, which facilitates the preferential deposition of Zn^2+^ at localized active sites, forming disordered and discrete nucleation sites that are detrimental to the uniform deposition of Zn^2+^ [52, 53]. More crucially, a self‐amplifying synergistic vicious spiral exists between the intensified electrode polarization induced by side reactions and the surface morphological deterioration of zinc electrodes. The non‐conductive passivation layer triggered by side reactions continues to thicken, leading to an exponential increase in interfacial resistance and a gradual increase in the surface roughness of zinc anodes [46, 54]. This roughened electrode surface further intensifies the uneven current distribution, creating a vicious cycle that persistently disrupts the interfacial kinetic equilibrium. Ultimately, under the combined effects of corrosion, HER, passivation accumulation, and dendritic growth, zinc anodes suffer irreversible capacity decay and a significant decrease in coulombic efficiency, resulting in the actual electrochemical performance of AZIBs still failing to meet the standards for practical application (Figure 2) [55].

**FIGURE 2:**
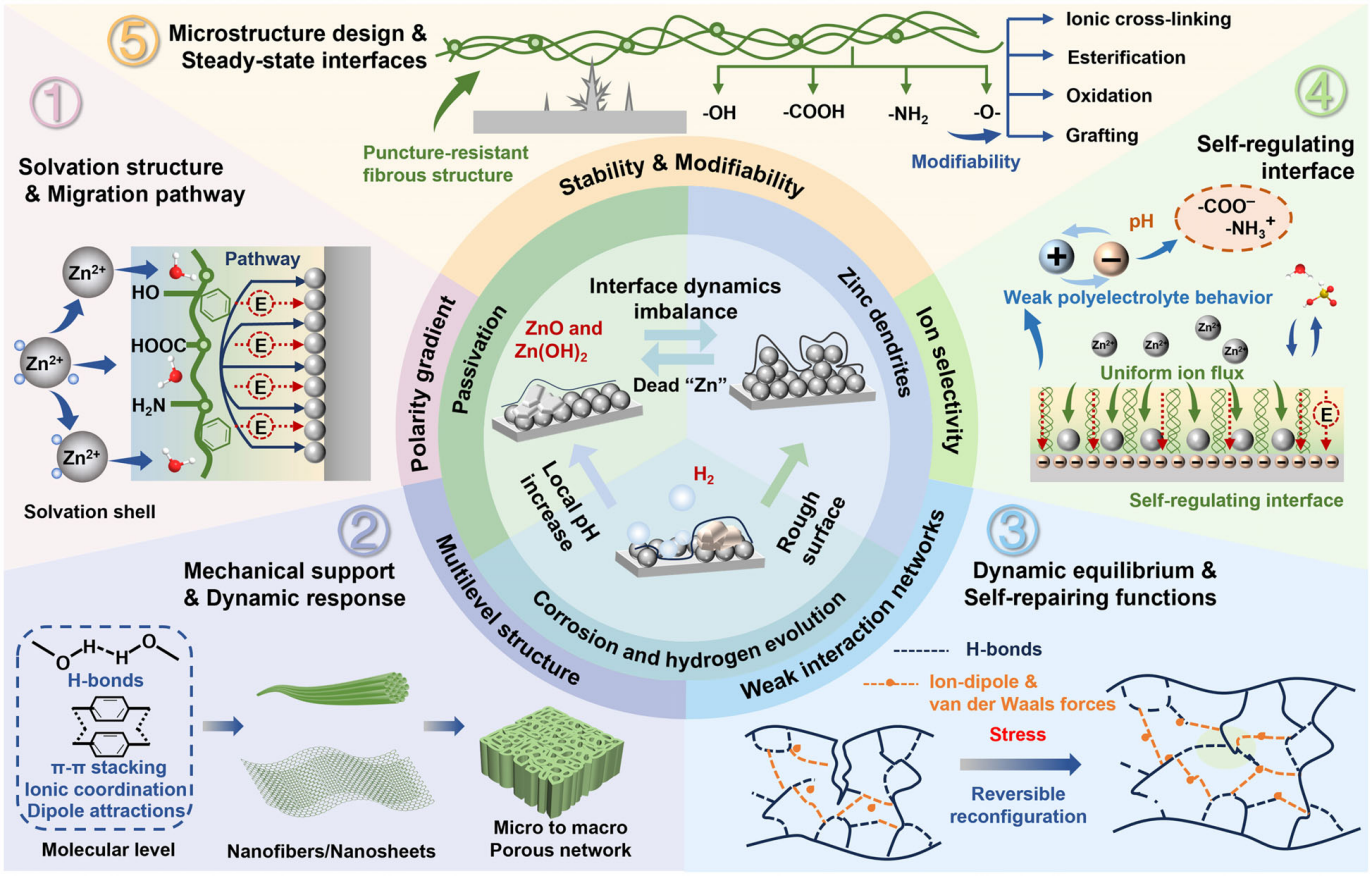
Analysis of the regulatory mechanism of natural macromolecular polymers in addressing zinc anode failure issues.

#### 2.1.1 Electrode Corrosion and Detrimental HER

Metallic zinc, as an active metal is prone to spontaneous corrosion reactions when used as an electrode in aqueous electrolytes. Once the zinc metal electrode is immersed in the weakly acidic zinc salt electrolyte solution adopted to improve the ionic conductivity of the electrolyte and the cycling stability of the battery, the corrosion of the zinc metal is further exacerbated, accompanied by a more pronounced corrosion HER (Equation (2)) [56, 57]. Meanwhile, during the deposition of zinc ions, the hydrated zinc ions bring water molecules to the surface of the zinc metal electrode, resulting in local enrichment of water molecules. H^+^ and water molecules attached to the electrode surface acquire electrons from zinc electrode, triggering the nonnegligible electrochemical HER side reactions that severely damage the stability of AZIBs (Equations (3) and (4)) [58, 59, 60, 61, 62]. Furthermore, zinc ions react with sulfate anions and water molecules to form insulating zinc sulfate hydroxide hydrate (Zn_4_SO_4_(OH)_6_·xH_2_O) byproducts (Equation (5)) [63, 64, 65]. These unwanted byproducts have a disordered structure and deposit unevenly on the zinc metal electrode, uncontrollably accelerating surface roughening of the electrode and irreversible capacity degradation of AZIBs [66, 67].

(2) $$\mathrm{Zn}+2H^{+}\to Zn^{2+}+2H_{2}↑$$

(3) $$2H^{+}+2e^{-}\to H_{2}↑$$

(4) $$2H_{2}O+2e^{-}\to H_{2}↑+2OH^{-}$$

(5) $$3Zn^{2+}+6OH^{-}+\mathrm{ZnS}O_{4}+xH_{2}O\to Zn_{4}SO_{4}{\left(\mathrm{OH}\right)}_{6}\cdot xH_{2}O$$

#### 2.1.2 Passivation Layer

During the electrochemical cycling of AZIBs, a passivation layer composed of byproducts such as ZnO and Zn(OH)_2_ usually forms on the surface of the zinc anodes. This occurs because, in a weakly acidic aqueous electrolyte, the corrosion HER at the zinc anode interface consumes H^+^, creating a localized highly alkaline environment, which in turn initiates more severe zinc corrosion (Equation (6)) and promotes the coordination of Zn^2+^ with hydroxyl ions (OH^‒^) to form Zn(OH)_4_^2‒^ complexes (Equation (7)) [68, 69]. Once the concentration of Zn(OH)_4_^2‒^ complexes exceeds the solubility threshold in the electrolyte, they decompose into ZnO or Zn(OH)_2_ byproducts, which then precipitate onto the surface of zinc anodes [70]. Concurrently, a portion of Zn^2+^ directly combine with OH^−^ at the interface to generate Zn(OH)_2_ byproducts. These sequentially generated byproduct sediments accumulate on the surface of the zinc electrode in a disorderly manner, gradually forming a passivation layer covering zinc anode. What's worse, this passivation layer is structurally loose, failing to form a dense physical barrier against aqueous corrosion [71, 72, 73]. Moreover, this passivation layer composed of byproducts with poor ion conductivity triggers an ion shielding effect during the Zn^2+^ stripping/deposition cycles, obstructing the efficient migration of Zn^2+^ and thereby slowing down the electrochemical mass transfer rate and reaction kinetics at the electrode‒electrolyte interface [46, 54]. Ultimately, this leads to an increase in the polarization voltage of the battery, significantly shortening the cycle life of AZIBs.

(6) $$\mathrm{Zn}+2H_{2}O+2OH^{-}\to \mathrm{Zn}{\left(\mathrm{OH}\right)}_{4}^{2-}+H_{2}↑$$

(7) $$Zn^{2+}+4OH^{-}↔\mathrm{Zn}{\left(\mathrm{OH}\right)}_{4}^{2-}$$

#### 2.1.3 Zinc Dendrite

In the Zn^2+^ deposition process, the generation of zinc dendrite is closely related to the transport kinetics of Zn^2+^, nucleation‐site distribution, and electric‐field uniformity. The transport of Zn^2+^ involves two modes: two‐dimensional (2D) surface diffusion and 3D interfacial diffusion. 2D surface diffusion, modulated by the surface morphology of electrode and the local electric field, causes zinc ions to deposit in a layer‐by‐layer manner. In contrast, 3D interfacial diffusion, which involves the vertical mass‐transfer process across the interface is considered more beneficial for ordered deposition of Zn^2+^. When the transport kinetics of Zn^2+^ is slow, 3D interfacial diffusion is restricted. Unordered 2D surface diffusion then dominates the deposition process, causing uneven deposition of Zn^2+^ and triggering uncontrollable growth of zinc dendrites [29, 74, 75, 76]. Moreover, zinc ions tend to nucleate preferentially at high‐energy sites such as grain boundaries and defects, forming initial zinc metal protrusions [77, 78]. Due to the tip effect, zinc ions will be directionally attracted to the protruding tips under the action of a local strong electric field, thereby leading to the formation dendrite structure [79, 80]. The inhomogeneous distribution of electric field further exacerbates the deposition deviation. Zinc dendrites tend to grow in regions of strong electric field, and their tips may penetrate the separator, causing a short circuit in the battery that severely shortens the cycle life of AZIBs [77, 81, 82, 83].

In AZIBs, the corrosion and passivation of zinc anodes, as well as the growth of zinc dendrites are interrelated and even mutually coupled to promote each other [57, 84, 85]. Typically, water molecules in the electrolyte trigger corrosion of zinc metal anodes and HER. As H^+^ is consumed and OH^‒^ continuously accumulates, the pH of localized regions within the electrolyte increases, promoting the formation of passivation layers composed of byproducts such as ZnO and Zn(OH)_2_. The loose passivation layer formed by the disorderly accumulation of byproducts impedes the transmission of Zn^2+^, resulting in a significant elevation in the polarization voltage of the battery and causing interface instability during cycling, thereby driving the growth of zinc dendrites [86]. Moreover, once zinc dendrites overgrow beyond their structural tolerance, large dendrites fracture into smaller fragments. These dendrite fragments that have detached from large dendrites disperse into the electrolyte, becoming “dead zinc” that no longer participates in normal electrochemical reactions within the battery [17, 87, 88]. These detrimental reactions of zinc dendrite growth and fragmentation cause substantial abnormal depletion of zinc anodes while severely reducing the Coulombic efficiency of the battery. All in all, the aforementioned harmful reactions form a vicious cycle through mutual coupling, constituting the central challenge to the stability of zinc anodes in AZIBs.

### 2.2 Natural Macromolecular Polymer‐Directed Optimization for Zinc Anodes

The aforementioned problems faced by zinc anodes are the key factors leading to the decline in the cycling stability of AZIBs. Therefore, for the coupling mechanism of the aforementioned side reactions, the optimization strategies for zinc anodes need to utilize materials with high polarity structure, moderate hydrophilicity, high mechanical strength, and dynamic response capabilities to effectively regulate the Zn deposition/stripping process at the zinc anode interface, thereby enhancing the stability of the zinc anode. Among various materials, natural macromolecular polymers have not only the aforementioned characteristics, but also their biodegradability, environmental friendliness, and abundant reserves, making them widely applied in the optimization strategies for zinc anodes [89]. Currently, the polarity gradient and micro‐phase distribution, multi‐level self‐assembly structure, weak interaction network, ion selectivity and electric field response ability, and chemical inertness and modifiability of natural polymers enable their application in the optimization strategies for anodes, effectively improving the cycling life of AZIBs and providing new opportunities for green and sustainable energy storage systems [90, 91, 92].

#### 2.2.1 Structural/Surface Characteristics‐Oriented Regulatory Mechanism

In response to the aforementioned issues faced by zinc anodes, the kinetics of Zn^2+^ could be improved and the interfacial side reactions of zinc anodes could be suppressed by stabilizing the pH of electrolyte, reducing the activation barrier for the desolvation of Zn^2+^, homogenizing the ion/electric field distribution, and enhancing the mechanical strength of the battery, thereby improving the stability of zinc anodes [93, 94, 95]. Therefore, the ideal materials required for optimizing zinc anodes should possess a series of targeted structural/surface chemical characteristics to address various challenges faced by zinc anodes from multiple levels and scales. In comparison with traditional inorganic materials and synthetic polymers, natural polymers demonstrate not only sustainability and cost advantages but also unique multifunctionality due to their multi‐level chain or network microstructures, highly tunable surface polarity and hydrophilicity, excellent mechanical properties and surface modifiability, as well as significant electric field responsiveness and self‐assembly capabilities, offering unparalleled benefits in optimizing zinc anodes [96, 97, 98, 99]. Thanks to their hierarchical structures and multifunctionality, natural polymers could exert multi‐level and multi‐faceted synergistic regulation on the electrochemical stability of zinc anodes, involving the following multiple regulatory mechanisms (Figure 2).

##### 2.2.1.1 Polarity Gradient and Microphase Distribution: Modulating Solvation Structure and Zn^2+^ Migration Pathway

Natural macromolecular polymer chains typically simultaneously contain a large number of hydrophilic functional groups (–OH, –COOH, –NH_2_, etc.) and hydrophobic carbon chains or aromatic ring segments [100, 101, 102]. This chemical asymmetry spatially forms a natural polarity gradient and microphase separation. The polar regions of natural polymer chains could form stable H‐bonds or ion‐dipole coordination with solvated Zn^2+^ and water molecules, while those nonpolar regions could restrict the penetration of highly active water molecules, maintaining the chemical stability of the natural polymer chain system in an aqueous environment [24, 103, 104, 105]. The constructed polarity‐differentiated microregions exert the dual functions of regulating Zn^2+^ transport pathways and inhibiting side reactions. Moreover, during electrochemical reactions, the ordered distribution of hydrophilic and hydrophobic regions induces the formation of built‐in dipole fields, which could automatically rearrange under an applied electric field to homogenize the local field strength, thereby mitigating the localization of interface electric fields and the uneven ion flux, especially alleviating the rapid failure of the zinc anode caused by the uneven ion/electric field distribution under high current density conditions [42, 106, 107, 108]. Therefore, natural macromolecular polymers could effectively optimize the solvation structure and transport pathways of zinc ions and promote the homogenization of the electric/ionic fields at the zinc anode interface, ensuring the stability of zinc anodes under high current density conditions. For instance, alginate‐ or cellulose‐derived polymer matrices can transiently coordinate Zn^2+^ through densely distributed oxygen‐containing groups, thereby constructing preferential migration sites and reducing local ion‐transport heterogeneity [26, 109].

##### 2.2.1.2 Multilevel Self‐Assembly Structure: Providing Mechanical Support and Dynamic Response Capability

Natural macromolecular polymers generally exhibit multilevel self‐assembly structures from the molecular level to the macroscopic level, arising from multiscale networks formed by abundant weak interactions between molecular chains [110, 111, 112]. At the molecular level, the chain segments are linked together through interactions such as hydrogen bonding, dipole attraction, π–π stacking, and ionic coordination to form local ordered crystalline regions or crystalline nuclei [113]. These interactions include the H‐bond networks between cellulose chains, the aromatic conjugated stacking of lignin, and the coordination crosslinking of Zn^2+^ in alginates [114, 115]. At the nanoscale level, multiple chains or crystalline regions could further assemble to form microfibers, nanosheets, or gel particles, which are typically driven by van der Waals forces, ionic cross‐linking, or physical entanglement [116, 117]. At the micro to macroscopic scale, these nanopolymers spontaneously would assemble into porous networks or frameworks, which achieve mechanical stability and the construction of ion transport channels under the support of fluid interface effects and weak physical crosslinking [118, 119]. This porous network structure enables the natural polymer‐based separators, gel electrolytes or interface layers to guide zinc ions to redistribute more evenly after passing through the pores, thereby homogenizing the electric/ion field on the surface of zinc anodes. Meanwhile, this structure effectively alleviates the excessive accumulation of local electrons/ions at surface protrusions on zinc anodes by spatially dispersing ion‐transport pathways, thereby achieving uniform deposition of zinc ions [120, 121].

Overall, the H‐bond networks provide support for molecular order and crystalline regions, ionic cross‐links form a steady 3D framework, van der Waals forces and chain entanglements strengthen the binding between microfibers, and the aromatic π–π interactions enhance structural rigidity and anti‐swelling properties [122, 123]. Their synergistic effects achieve the spontaneous assembly and dynamic maintenance of the structure at different scales, endowing the natural polymer‐based systems with reversible responsiveness and energy buffering capabilities. When external electric fields or ion concentrations change, the microscopic chain segments could rearrange or reassociate, causing the macroscopic networks to adjust accordingly to restore the steady state [124, 125]. This reversible multilevel self‐assembly capability is precisely the fundamental source of the high toughness, self‐healing capability, high dynamic responsiveness, and structural and interfacial stability of natural macromolecular polymer‐based materials.

##### 2.2.1.3 Weak Interaction Networks: Endowing Interface with Dynamic Equilibrium and Self‐ Repairing Functions

The weak noncovalent bonds such as H‐bonds, ion‐dipole interactions, and van der Waals forces that are ubiquitous in natural macromolecular polymers form a flexible and stable weakly cross‐linked network within them [126, 127]. Although these interactions have low energy, they are highly reversible and can constantly undergo breakage and reformation during electrochemical cycles, endowing natural polymer‐based materials with unique dynamic adaptability [128, 129]. When zinc ions are deposited or stripped at the electrode surface, local volume variations, and morphological fluctuations are prone to trigger microcracks and interface stress concentration [130, 131]. In such cases, the reversible restructuring of the weak bond network could rapidly repair these microcracks through chain segment migration and H‐bond regeneration, maintaining the continuity, and compactness of the coating layer, thereby effectively prolonging the lifespan of the natural polymer‐based interface layers. This dynamic equilibrium behavior is achieved through the intrinsic response mechanisms of the natural polymer structures, which not only effectively alleviates structural issues caused by the deposition/stripping of zinc ions, but is also highly effective in addressing internal stress and electro/ionic field imbalances within AZIBs during long‐term cycling [32, 115].

Since the weak bond network between molecule chains of natural macromolecular polymers could spontaneously rearrange under external forces, electric fields, or changes in ion concentration, natural polymer systems would reestablish local equilibrium at different cycling stages, preventing cracks on them from further propagating. Specifically, the H‐bond breakage‐reformation process provides self‐healing capability and a rapid energy dissipation pathway, the ion‐dipole coordination maintains the chemical continuity of the interface, and the van der Waals entanglement ensures the connection stability of the overall framework [24, 101]. The synergistic action of three weak interaction networks enables natural polymer system to maintain an intact interface through a “soft stress buffering‒structural self‐integration” manner during long‐term electrochemical cycling. For instance, cellulose contains abundant β‐1,4‐glycosidic linkages and hydroxyl groups, which construct a dynamic H‐bond network. This structural feature enables cellulose‐based interfacial layers to better dissipate stress and suppress crack propagation during the repeated deposition/striping process of zinc ions [114, 132].

##### 2.2.1.4 Ion Selectivity and Electric Field Response: Establishment of Self‐Regulating Interface

Natural polymers contain abundant ionizable groups (e.g., –COOH, –OH, and –NH_2_), which could partially ionize in aqueous solution either in their native form or after chemical modification, depending on the environmental pH, ionic strength, and electrode potential, exhibiting typical weak polyelectrolyte behavior [133, 134, 135]. This characteristic enables the surface charge density and polarity of these natural polymers to be dynamically adjusted, thereby imparting them with selective ion conduction and self‐regulating electric field capabilities during electrochemical reactions [136, 137]. In neutral or weakly acidic environments, the –COOH groups of the alginates would partially dissociate into –COO^‒^, while the –NH_2_ groups of chitosan could be protonated to –NH_3_^+^ [32, 138]. The coexistence of these two groups in the electrolyte allows them to mutually regulate each other based on the actual pH environment, forming a mixed‐type charge network containing both positive and negative charges.

When the natural polymers with polar functional groups are used for modifying zinc anodes or constructing gel electrolytes, these self‐regulating charge groups self‐assemble at the interface to form a uniform ion‐regulating layer [123, 139]. On the one hand, the negatively charged regions could preferentially attract Zn^2+^ and guide its directional migration while repelling SO_4_^2‒^, significantly inhibiting electrode corrosion and other side reactions. On the other hand, the adjacent regions formed by positively charged or neutral groups could block excessive Zn^2+^ accumulation, resulting in a more uniform overall ion flux, thereby effectively evading the interfacial tip effect caused by aggregated deposition of Zn^2+^ on zinc anodes [105, 140]. Moreover, their weak polyelectrolyte properties endow these natural polymers with excellent electric field response characteristics [42]. The electric field response capability and ionic selectivity jointly enable these natural polymers to achieve self‐regulation at the electrode interface, not only physically blocking active water but also responding to environmental changes to achieve synergistic optimization in ion sieving, electric field homogenizing, and pH buffering.

##### 2.2.1.5 Chemical Stability and Modifiability Coexist: Targeted Microstructure Design and Constructing Steady‐State Interface

The stable carbon‐oxygen‐carbon or aromatic frameworks confer excellent chemical corrosion resistance to natural macromolecular polymers in highly polar media [141, 142]. Compared with traditional synthetic polymers, these natural polymers mostly derived from biological polysaccharides or aromatic polymers, with higher covalent bond energy and lower chemical reactivity. Therefore, they could maintain continuous chain structures and excellent chemical tolerance even in acidic, alkaline environments or under high potential conditions. At the same time, the abundant reactive functional groups distributed on their molecular chains, such as –OH, –COOH, –NH_2_ and ether linkage (–O–), endow them with modifiability and multifunctional regulation capabilities [143, 144]. Through appropriate chemical modifications, such as ionic crosslinking, oxidation, esterification, or grafting reactions, the density and spatial distribution of functional groups on these natural polymers could be precisely adjusted according to the optimization goals of zinc anodes [145, 146]. For instance, increasing the content of –COOH or carbonyl groups could strengthen the interaction between natural polymer chains and Zn^2+^, promoting Zn^2+^ to nucleate and deposit more uniformly, thereby smoothing the deposition morphology and inhibiting the formation of zinc dendrites [42, 147].

Additionally, introducing hydrophobic alkyl or aromatic chain segments would moderately enhance the ability of natural polymer chains to reduce the activity of free water, thereby effectively facilitating the desolvation of hydrated Zn^2+^ and reducing the probability of water‐induced side reactions, endowing the electrode interface with a higher electrochemical stability [100, 148]. Through these chemical and structural fine‐tuning, a dynamic equilibrium between ion conductivity and interface stability could be achieved. Moreover, the diversity of surface functional groups also enables these natural polymers to have extremely high structural designability. Through ionic crosslinking or hybrid assembly, these natural polymer chains could form multiscale structures such as porous membranes rich in nanoscale channels and micrometer‐scale pores, demonstrating controllable characteristics in terms of mechanical support, ion‐selective channels, and electrolyte retention capabilities [132, 149]. The coexistence of chemical stability and modifiability of these polymers enables them to provide a stable and corrosion‐resistant framework in optimizing zinc anodes, while achieving precise regulation of the electrochemical environment at the electrode interfaces through functional group engineering, laying a solid foundation for constructing of highly stable and reversible zinc anodes.

#### 2.2.2 Typical Natural Macromolecular Polymers Applied in Optimizing Zinc Anodes

##### 2.2.2.1 Chitin‐Based Materials

Chitin is the second most abundant renewable natural macromolecular polymers in the natural world, a natural polysaccharide widely present in the exoskeletons of marine crustaceans (e.g., shrimp, crabs) and organisms like insects and fungi [150, 151]. It possesses excellent biocompatibility and biodegradability. Furthermore, the substantial ‒OH and acetylamino (‒NHCOCH_3_) groups within chitin molecular chains not only form extensive intramolecular and intermolecular H‐bond networks, but also endow chitin with favorable amphiphilicity and render it readily amenable to modification [152, 153]. Although these formed intramolecular and intermolecular H‐bond networks confer high mechanical strength upon chitin, they simultaneously reduce its solubility in neutral or alkaline solutions and diminish its affinity for electrolytes, which somewhat limits its direct application in AZIBs [154]. In contrast, as the most representative derivative of chitin, chitosan features numerous protonatable ‒NH_2_ groups distributed along its linear molecular chains, which significantly improve its solubility in acidic aqueous solutions. Typically, when approximately 50% of the ‒NH_2_ groups are protonated, chitosan becomes soluble in aqueous solutions [155, 156, 157]. In addition to being rich in free ‒NH_2_ groups, chitosan—a linear polysaccharide derived from chitin through deacetylation, composed of glucosamine units linked by glycosidic bonds—also contains numerous ‒OH and ‒O‒ along its molecular chains [158, 159]. These functional groups provide abundant reactive sites for further functionalization of chitosan. Given that chitosan possesses high mechanical strength, rich organic functional groups, and hydrophilicity after protonation, researchers have fabricating a variety of chitosan‐based functional materials suitable for AZIBs through strategies such as chemical modification or composite formation with other materials, and extensively applied them for protecting zinc anodes in AZIBs [101, 160, 161]. Although chitin exhibits lower water solubility than chitosan, its outstanding mechanical properties and high chemical reactivity brought about by numerous functional groups have also enabled it to be applied in multiple ways for improving zinc anodes. To date, chitin‐based functional materials, primarily composed of chitin and chitosan, have achieved a series of important advancements for improving the stability of zinc anodes in AZIBs [38, 101, 120, 162].

##### 2.2.2.2 Cellulose

Cellulose, as the most abundant natural macromolecular polymers can be extracted from various biological systems like plants (trees and algae), bacteria, and so on. As a polysaccharide polymer composed of glucose molecules, it contains numerous ‒OH groups, leading to the formation of numerous hydrogen bonding within and between its molecular chains [163]. The numerous intermolecular H‐bonds render cellulose poorly water‐soluble, resulting in inferior wettability of membrane materials derived from it and limiting its application in optimizing zinc anodes [164]. In order to enhance the applicability of cellulose in AZIBs, a series of physical/chemical treatments (e.g., strong acid treatment, intense ultrasonication, high‐shear mixing, etc.), surface functionalization and molecular structural design (such as etherification, esterification, oxidation, grafting, etc.) have been applied to elevate its water solubility [123, 140, 165]. Beyond these aforementioned direct modification strategies for producing hydrophilic cellulose‐based materials, compositing cellulose with inherently highly hydrophilic materials to construct the overall hydrophilic composites also represents an effective approach for preparing cellulose‐based functional materials aimed at optimizing zinc anodes [166, 167, 168, 169]. On the other side, the abundant ‒OH groups on cellulose can promote the uniform transport of zinc ions and guide their even deposition, which is conducive to the application of cellulose‐based materials in AZIBs [42, 123, 125]. Thus, the modified cellulose and cellulose‐based composites integrate multiple advantages such as abundant ‒OH groups, excellent mechanical properties, and good compatibility with aqueous electrolytes. The application research of such materials in optimizing zinc anodes has been continuously deepened, attracting the research interest of numerous scientific researchers [170].

##### 2.2.2.3 Lignin

Lignin is a carbon‐rich biopolymer whose structure consists of three main units: guaiacyl (G), syringyl (S), and p‐hydroxyphenyl (H), which are connected via carbon‐carbon bonds and –O– bonds [171, 172]. The molecular structure of lignin involves numerous reactive groups such as methoxy (‒OCH_3_), ‒COOH, carbonyl (‒CO), ‒OH, and conjugated double bonds (C═C‒C═C) [173, 174]. Furthermore, lignin also boasts high thermal stability, high mechanical strength, high charge density, and abundant carbon‒ and heteroatom‒ containing functional groups, endowing it with significant potential for energy storage [173, 174, 175, 176, 177, 178]. However, the numerous H‐bonds existing between the molecules of lignin seriously reduce its water solubility, complicating its application in AZIBs [179, 180, 181]. In particular, lignin typically exhibits low ionic conductivity, and its application is not conducive to the efficient transmission of zinc ions in AZIBs, resulting in an increase in the polarization voltage of the battery. Currently, improving the electrochemical performance of lignin through strategies such as chemical modification (such as sulfonation, carboxylation, chemical crosslinking, etc.), physical modification (such as blending with polymer materials and physical crosslinking, etc.) and structural regulation, and combining its inherent advantages (such as multiple functional groups, high mechanical strength, etc.) to expand its application in AZIBs has become a research hotspot [182, 183, 184]. With the advancement of research, an increasing number of lignin‐based materials have been applied to AZIBs, aiming to achieve effective optimization for zinc anodes.

##### 2.2.2.4 Alginate

Alginate is a natural polysaccharide extracted from brown algae such as seagrass, macroalgae, and kelp, consisting of linear chains composed of β‐D‐mannuronic acid (M) and α‐L‐guluronic acid (G) [185, 186]. One notable characteristic of alginate is that it can coordinate and cross‐link with many kinds of metal cations to generate hydrogels with high ionic conductivity and abundant organic functional groups [187, 188]. In recent years, some alginate‐based hydrogels have been attempted as gel electrolytes for AZIBs, demonstrating their ability to mitigate the detrimental effects of active water on zinc anodes. These significant research advancements have further stimulated broader applications of alginate in AZIBs [189]. Whereas, alginate‐based hydrogels typically exhibit poor elasticity and mechanical properties, along with insufficient stability. These deficiencies result in suboptimal interface contact between alginate‐based hydrogels and electrodes in AZIBs, posing a risk of being punctured by zinc dendrites. To construct alginate‐based hydrogels that can more effectively optimize the performance of AZIBs, some researchers have enhanced the elasticity, mechanical strength and thermal stability of alginate hydrogels by synthesizing organic‐inorganic alginate‐based composite materials [190]. Moreover, a few of the latest studies have developed highly flexible porous alginate‐based hydrogel electrolytes with uniform pore structures to fulfil controlled regulation of the transport and deposition of zinc ions, improve the compatibility of the electrolyte‐electrode interface, thereby achieving effective optimization of zinc anodes in AZIBs [32, 109].

In summary, these representative natural polymers with wide sources, including chitin, cellulose, lignin, and alginate have shown considerable potential for zinc anode optimization in AZIBs. Their abundant functional groups, structural diversity, and intrinsic renewability enable them to play multiple roles in enhancing the stability of zinc anodes and AZIBs, including dendrite suppression, side‐reaction inhibition, and battery structure reinforcement (Figure 3). Although some intrinsic drawbacks still restrict their direct large‐scale application, various strategies such as chemical modification and structural engineering are continuously broadening the prospects for the application of these natural polymers in AZIBs.

**FIGURE 3:**
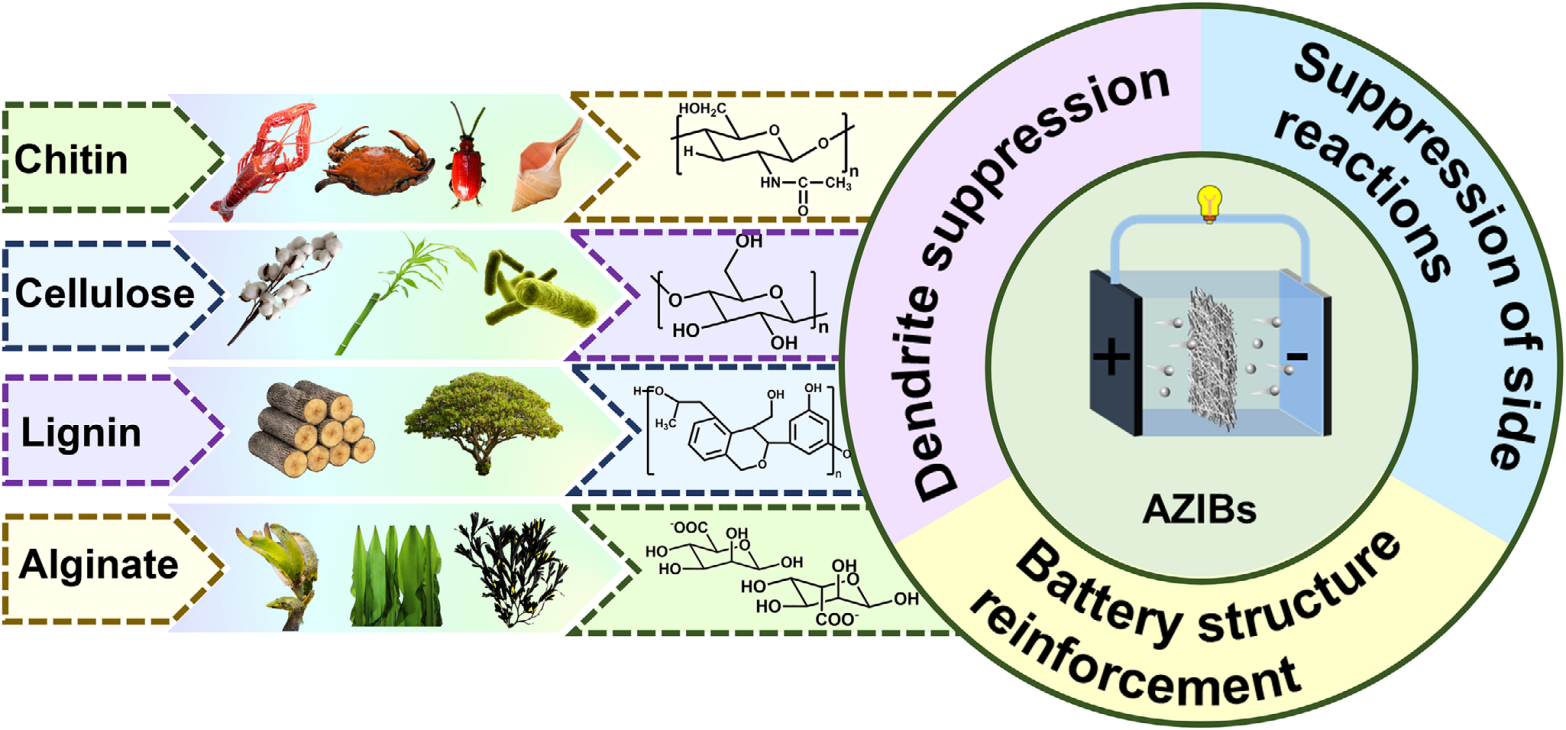
The sources of natural macromolecular polymers and the regulatory strategies for protecting zinc anodes in AZIBs.

#### 2.2.3 Multiscale Structure‐Function Relationships of Natural Macromolecular Polymers in Optimizing Zinc Anodes

The optimization effect of natural macromolecular polymers on zinc anodes arises from the multiscale coupling of microscopic and mesoscale structural features. Analyzing this multiscale coupling and establishing the interrelationship between the microscopic/mesoscopic structure features of natural polymers and their multiple functions is crucial for elucidating the intrinsic advantages of such polymers and their key role in enhancing the cycling stability of zinc anodes in AZIBs.

At the microscopic level, the abundant functional groups on natural polymers and their molecular chain structures exert the important regulatory effects on the electrode interface/electrolyte microenvironment [191, 192]. These polymers contain a variety of polar groups, which can strongly interact with Zn^2+^, H_2_O, and anions in the electrolytes through coordination, ionic‐dipole interactions, and H‐bonds. Previous studies, especially those based density functional theory (DFT) calculations have shown that the binding energies of these polar groups to Zn^2+^ typically decrease in the order of amide > –COOH > –OH > –NH_2_ [42, 46, 193, 194], and all exceed the binding energy of Zn^2+^–H_2_O coordination (The binding energy may sometimes vary due to the different chemical properties of the electrolyte and the electrochemical conditions). These interactions can reconstruct the local solvation environment of hydrated Zn^2+^, weaken Zn^2+^–H_2_O coordination, and facilitate the desolvation of Zn^2+^ prior to deposition. Since desolvation is a major kinetic barrier for the deposition of Zn^2+^, this molecular‐level regulation directly affects the nucleation behavior of Zn^2+^ and their deposition reversibility [195, 196]. Meanwhile, the interactions between polymer chains and H_2_O can reduce the activity of free water near the electrode surface and block its reaction with zinc anodes, thereby suppressing electrode corrosion and hydrogen evolution side reaction [103, 197].

Furthermore, the functional behavior of natural polymers depends not only on the type of functional groups but also on their density, spatial distribution, and configurational continuity [198, 199]. For instance, continuously and uniformly distributed zincophilic functional groups can create appropriate pathways for the desolvation and migration of Zn^2+^, thereby homogenizing ion flux and eliminating electric current/electric field localization [25, 200, 201, 202]. In contrast, both sparse and overly aggregated functional groups may be detrimental to the rapid transport of Zn^2+^ and may trigger uneven nucleation. Similarly, aside from chain length and configuration, chain continuity and flexibility, as well as the microstructure formed by interchain interactions, also significantly affect the functional behavior of natural polymers in optimizing zinc anodes [134, 203]. Based on the above analysis, it can be roughly concluded that the microscopic structure‐function relationship of natural polymers is mainly composed of the following four key factors: (1) The specific chemical properties of functional groups, determined by their type, govern their affinity for Zn^2+^ and other ions. (2) The density and distribution of functional groups jointly regulate the transport of Zn^2+^ and their distribution on the electrode surface. (3) The length of the polymer chains, their interlaced/entangled state, and the spatial distribution of the polar microregions within the formed chain network all affect the directionality and uniformity of zinc ion transport. (4) The continuity or flexibility of the main chain of the polymers determines whether these polymers can respond promptly to the dynamic changes of the interface environment on zinc anodes.

More notably, these factors not only act individually and need to be optimized in terms of quantity and state, but their mutual coupling together constitutes the microscopic structure‐function relationship of natural polymers in optimizing zinc anodes. Typically, a high density of polar groups is beneficial within a certain threshold, but too many groups can form excessively strong interactions with Zn^2+^, hindering ion transport and impairing the uniformity of Zn^2+^ deposition [26, 132]. Likewise, the long chains of natural polymers, or the networks they form can adsorb onto the surface of zinc anodes to create a protective interface, while the free zinc‐affinity functional groups on their side chains accelerate the desolvation of zinc ions and promote their deposition. Simultaneously, these polymers must maintain sufficient chain flexibility and appropriate chain length, otherwise polarization phenomena (or even gelation) may occur due to excessive adsorption or aggregation of the polymers [204, 205]. In addition, since the polar functional groups are loaded on the polymer chains, the extension and entanglement states of the chains can significantly regulate the spatial distribution, accessibility, and action behavior of the functional groups [206]. Excessive entanglement of the chains hinders the interaction between the functional groups on them and the ions in the electrolyte as well as the electrode interface. In contrast, the interaction of various functional groups can also affect the orientation and entanglement state of the polymer chain. Similar to the aforementioned cases, more such coupled effects collectively regulate the optimization effect of natural polymers on the zinc anode. More coupling effects similar to those mentioned above jointly regulate the influence of natural polymers on the transport and distribution of ions as well as the electrode interface state, thereby adjusting their optimization effect on the stability of zinc anodes.

At the mesoscopic scale, the durability of natural polymers in stabilizing zinc anodes is determined by their self‐assembled morphology, chain entanglement structure, pore/channel architecture, and structural compactness [31, 153, 207]. Because the zinc anode/electrolyte interface in AZIBs is intrinsically dynamic, undergoing repeated depositing/stripping, volume fluctuation, stress accumulation, and chemical environment variation, its stability cannot be significantly enhanced solely by microscopic chain structure and functional groups. Fortunately, the hierarchical structure of chain segments, 3D networks, and so on formed by natural polymer chains can provide continuous ion transport channels, homogenize ion flux and electric field distribution at the electrode interface, and promote interface reconstruction, thereby optimizing the stability of the zinc anode at the mesoscopic scale [208, 209]. That is to say, the mesoscopic structure‐function relationship of natural polymer in stabilizing zinc anodes is reflected in how hierarchical architectures optimize ion transport/distribution and electric field distribution, as well as improve the corrosion resistance and self‐adaptability of the electrode interface. More specifically, the porous/rich‐channel characteristics and chain network of natural polymer matrices compactness exert dual effects on the stability of zinc anodes: ordered transport channels facilitate directional ion diffusion and alleviate concentration polarization near the surface of zinc anodes, while an appropriately dense chain network/H‐bond network can mechanically suppress dendrite growth, prevent direct contact between free water and the electrode interface, and enhance the self‐healing ability of the electrode interface [99, 210]. Accordingly, the optimization effect of natural polymers on zinc anodes at the mesoscopic scale has gone beyond simple physical blocking, arising instead from the synergistic coupling of ion/electric field homogenization, stress buffering, and interface self‐healing guided by their hierarchical structures.

Overall, the stabilizing effect of natural polymers on zinc anodes is jointly governed by structure–function relationships across the microscopic and mesoscale regimes. At the microscopic level, functional groups and chain configurations regulate the transport behavior of Zn^2+^ and suppress interfacial side reactions. At the mesoscopic scale, the formed hierarchical architectures not only homogenize the ion/electric field on the electrode surface and enhance interfacial chemical stability, but also determine whether these advantages can be maintained during prolonged electrochemical cycling. It is precisely this multiscale structure‐oriented synergistic optimization that makes natural polymers particularly important and significantly superior in stabilizing zinc anodes.

### 2.3 Comparison with Traditional Material‐Oriented Optimization Strategies

Currently, traditional materials such as carbon‐based materials (e.g., graphene, carbon nanotubes) [211, 212], metal oxides/sulfides (e.g., TiO_2_, ZnS) [213, 214, 215, 216, 217], polymers (e.g., polyethylene glycol) [214, 218], and alloy materials (e.g., Zn‐Cu alloy) [218, 219, 220] have achieved significant progress in optimizing zinc anodes. However, these materials are confronted with challenges including poor environmental friendliness, high cost, and limited functionality [221], resulting in insufficient sustainability and restricting their further application expansion in the field of AZIBs (Figure 4). By contrast, natural macromolecular polymers, with their core advantages such as renewability, multi‐functional integration, and environmental friendliness have demonstrated significant potential in stabilizing zinc anodes, offering a novel pathway for the sustainable development of AZIBs [20, 222, 223].

**FIGURE 4:**
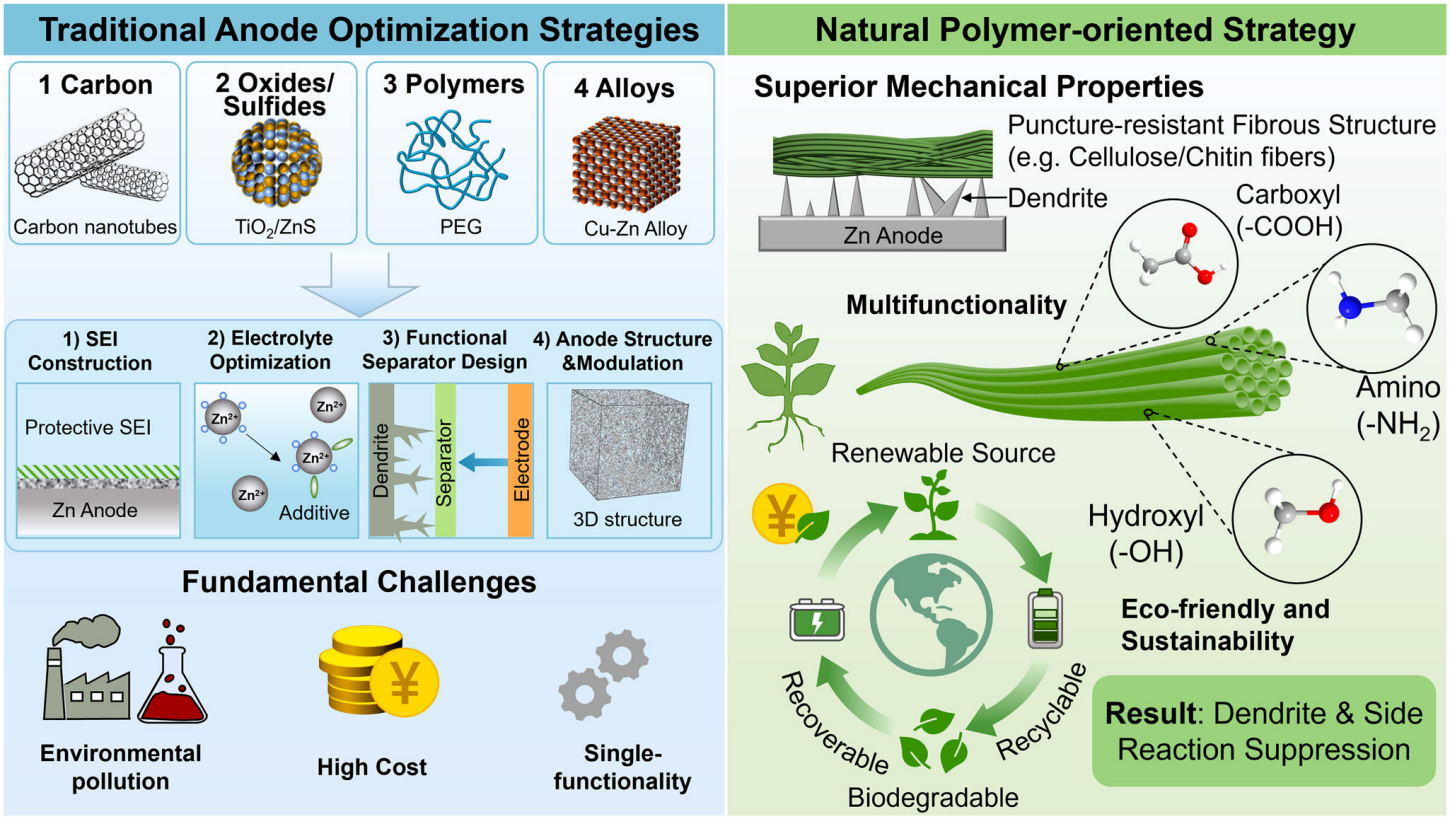
Comparison of natural macromolecular polymer‐oriented strategies with traditional material‐oriented optimization strategies.

#### 2.3.1 Traditional Material‐Oriented Optimization Strategies for Zinc Anodes

In existing researches, various optimization strategies based on traditional materials have been designed to overcome the challenges faced by zinc anodes during cycling in AZIBs [37, 224]. These optimization strategies primarily focus on the following aspects:

Artificial SEI layer: Establishing an SEI protective layer on the surface of zinc anodes via in situ or ex situ construction methods can effectively regulate the deposition behavior of zinc ions on the surface of zinc anodes, providing direct protection for zinc anodes and suppressing interfacial side reactions [225, 226]. Engineering the SEI layer is recognized as a direct and efficient approach to significantly enhance the cycling performance of AZIBs [227]. Specifically, it is because the SEI layer can effectively shield zinc anodes from HER and electrode corrosion side reactions while inhibiting the disorderly growth of zinc dendrites [33, 228, 229].

Electrolyte optimization: Electrolyte additives enable effective regulation of key electrolyte parameters, including the solvation structure and deposition behavior of zinc ions, as well as the pH value and ionic conductivity of the electrolyte, which optimizes the electrochemical reversibility of zinc anodes during cycling, thereby enhancing the cycling stability of AZIBs [15, 204, 230, 231, 232, 233]. Additionally, the gel electrolytes designed based on conventional materials prevent the uncontrollable side reactions caused by excessive contact between zinc anode and active water, thereby effectively optimizing zinc anodes [8, 234, 235, 236, 237].

Separator design: Separator design encompasses a variety of approaches, including modifying conventional commercial separators with functional materials and constructing novel self‐supporting separators based on functional materials [140, 238]. These functionalized commercial separators and novel self‐supporting separators typically exhibit superior regulation of zinc ion transport when replacing conventional commercial separators [239]. By leveraging the advantages of functionalized design, they can promote the desolvation of zinc ions, accelerate the transport kinetics of zinc ions, and uniformize the surface electric field distribution on zinc anodes, thereby effectively restraining the side reactions and dendrite growth on zinc anodes [240].

Anode composition and structure Regulation: The composition and structure of zinc‐based anodes can be effectively regulated through a series of methods such as constructing zinc‐based alloys, designing 3D porous structures, and fabricating densified structures, which can enhance the mechanical strength and corrosion resistance of zinc‐based anodes, homogenize the electric field distribution on the electrode surface, and optimize the deposition behavior of zinc ions, thereby suppressing the growth of zinc dendrites [241, 242]. Ultimately, this yields AZIBs with outstanding cycling stability.

#### 2.3.2 Advantages Over Traditional Material‐Oriented Optimization

Traditional materials such as graphene [211], oxides/sulfides [213, 214], and synthetic polymers [214, 218] have been extensively used to optimize zinc anodes and have significantly improved the cycling stability of zinc anodes due to their respective advantages. To be specific, graphene, valued for its exceptional conductivity and mechanical strength is widely employed in constructing artificial SEI layers [243]. Metal oxide coatings can suppress dendrite growth through physical blocking [213, 244, 245]. Synthetic polymers offer good interfacial compatibility, and their main chain/side chain structures, molecular weight, and crosslinking density can be reasonably controlled, thus having significant advantages in controllably regulating the transport and deposition behavior of Zn^2+^ as well as the mechanical properties of the electrode interface [218, 246]. Currently, synthetic polymers remain an important class of materials in the interfacial engineering of zinc anodes, continuously attracting considerable research interest.

Unlike traditional materials, which are artificially designed and manufactured, natural macromolecular polymers are typically sourced directly from nature and are extracted from abundant biomass. In addition, their inherent hierarchical structure and naturally distributed surface polar functional groups (amide, –COOH, –OH, –NH_2_, etc.) enable them to regulate the transport and deposition of Zn^2+^ and suppress side reactions at the electrode interface without the need for sophisticated structural design and surface group grafting, as is required for traditional materials [247, 248]. For instance, cellulose can be extracted from cotton, wood, cornstalks, rice straw, and even acetobacter xylinum, featuring stable cellulose chains/networks and abundant hydrophilic functional groups [123, 249]. Leveraging its advantages in structure and surface properties, cellulose can optimize zinc anodes by chelating zinc ions, adsorbing on the electrode surface, buffering pH, and constructing ion‐transport channels [21, 250]. It is precisely these innate hierarchical structures and diverse surface functional groups that endow natural polymers with remarkable multifunctionality, enabling them to stabilize zinc anodes across multiple scales and thereby distinguishing them from traditional materials. Moreover, there are still considerable challenges in designing and manufacturing traditional materials that can rival the diverse hierarchical structures and functional group‐rich surface characteristics of natural polymers. Meanwhile, natural polymers also share similar characteristics with traditional materials, such as excellent tunability of structural and surface properties. Therefore, these polymers can also achieve a balance between performance, cost and environmental benefits through further molecular structure design and functional synergy [251, 252].

Generally, the flexibility and biocompatibility of natural polymers are superior to traditional materials such as synthetic polymers, which is attributed to their source and chain‐like microstructure. Therefore, natural polymers are often used to assemble flexible AZIBs, thereby playing an important role in flexible electronics, wearable devices, and biomedicine [98, 253]. In addition, it is worth noting that the entangled chains, networked microstructure, H‐bond networks, and functional group‐rich surfaces of these natural polymers provide a foundation for further optimization of many of their properties, such as mechanical performance. Some natural polymers with optimized microstructures possess mechanical strength, interfacial compatibility, and self‐healing abilities that exceed those of synthetic polymers and exhibit outstanding performance in stabilizing zinc anodes [99, 254]. One of the more common ones is to use these optimized polymers to construct the SEI layer for protecting zinc anodes. The novel SEI protective layers constructed from these polymers typically exhibit higher mechanical strength compared to the traditional material‐derived SEI layers, enabling more effective protection of the zinc anode interface and inducing Zn (002) deposition [108, 255]. Simultaneously, these natural polymer‐derived SEI layers possess in situ self‐healing capabilities, whose internal dynamic hydrogen‐bonding networks, imparted by the biomass materials, can in situ repair their own structural damage as well as microcracks at the electrode interfaces, thereby enhancing the cycling stability of zinc anodes [41, 256].

The inherent functional diversity represents another core advantage of natural polymers in improving the performance of zinc metal anodes. Among numerous traditional materials, synthetic polymers that are structurally similar to natural polymers and possess excellent designability can achieve specific functions through structural design [257, 258], but this process usually requires precise regulation of the distribution and substitution degree of functional groups as well as the chain structure. Moreover, these regulations often rely on strict and controllable reaction conditions, and the process is complex and time‐consuming [259]. In contrast, the inherent uniformly distributed polar functional groups and hierarchical self‐assembled structures of natural polymers endow them with adjustable surface polarity/hydrophilicity, surface/structure adjustability, excellent mechanical properties, significant electric field responsiveness and self‐assembly capabilities. Consequently, these inherent characteristics confer natural advantages to such polymers in optimizing ion transport, regulating the zinc anode interfaces, and interface self‐repairing as well as suppressing side reactions, demonstrating remarkable multifunctionality [109, 260]. It is worth noting that, in practical applications, factors such as source variability, structural heterogeneity, processing stability, and compatibility with the electrolyte system must still be carefully considered to ensure that the advantages of natural polymers can be stably and effectively exerted.

From the perspectives of eco‐friendliness and sustainability, natural polymers offer more pronounced advantages over traditional materials. These polymers mostly originate from renewable resources like crops, forestry products, and herbaceous plants, which can be continuously supplied through the natural cycle growth of these renewable resources [91, 261]. Additionally, the production and modification processes of these polymers feature low carbon emissions, which is conducive to environmental protection. Also, these materials are only recyclable and reusable but also biodegradable, helping to alleviate the secondary environmental pollution caused by their disposal [262]. Economically, natural polymers have a wide range of sources and are derived from agricultural and forestry plant waste, typically costing significantly less than many conventional materials. Currently, cellulose and lignin production has already basically achieved industrialization with low costs, while the cost of chitin and alginates can be reduced by utilizing the waste generated during aquaculture and algal farming as feedstock [90, 263]. Moreover, natural macromolecular polymers can be engineered to customize their performance through the modification of functional group (e.g., sulfonation, esterification) and composite designs (e.g., MXene/cellulose heterostructures), balancing electronic and ionic conductivity as well as mechanical strength, and breaking the performance limitations of traditional materials [264, 265, 266].

Overall, traditional materials such as synthetic polymers can be customized for target functions through rational molecular and structural design, playing an important role in optimizing the cycling stability of zinc anodes. At present, a variety of cost‐effective traditional functional materials with mature synthesis techniques have been widely attempted to be used in AZIBs, providing an alternative material library for effectively improving the stability of zinc anodes. To further push zinc metal anodes toward practical applications, it is necessary to develop materials with multiple functions to address the various challenges faced by zinc anodes. Synthetic polymers, as representative traditional materials have great potential in developing multifunctional new materials due to their significant surface/structural designability and have been extensively studied in recent years. A series of multifunctional synthetic polymers have been developed for the optimization of zinc anodes. However, the integration of multiple functions in synthetic polymers requires complex design and cumbersome synthesis processes. Moreover, the more diverse the functions, the higher the manufacturing cost and synthesis difficulty of synthetic polymers, and the lower the yield. Therefore, promoting the large‐scale application of zinc anodes by constructing multifunctional synthetic polymers capable of fully addressing all associated issues faces obstacles such as the difficulty of synthesis, low yield, and high cost of such polymers. In contrast, natural polymers, due to their inherent multifunctionality, hierarchical structures, good flexibility, environmental friendliness, and sustainable raw materials are considered to have great potential in improving the stability of zinc anodes and promoting their large‐scale practical application. Although there has been an increasing number of related studies in recent years, the regulatory mechanisms and the involved structure‐function relationships of various natural polymers in stabilizing the zinc anode have not been systematically analyzed and summarized. To advance the large‐scale practical application of natural polymer‐based zinc metal anodes, these regulatory mechanisms and structure–function relationships deserve in‐depth analysis and study.

## 3 Applications of Natural Macromolecular Polymers for Optimizing Zinc Anode

To advance the green development and low‐cost practical application of AZIBs, natural macromolecular polymers, which are widely available, environmentally friendly, renewable, biodegradable, inexpensive, and possess unique physicochemical properties, have been increasingly developed in recent years to address the most challenging issue in AZIBs, that is, how to improve the cycling stability of zinc anodes [20, 90, 180, 267]. The abundant surface functional groups and diverse morphologies of natural polymers enable them to exhibit pronounced advantages in overcoming issues such as uncontrolled dendrite growth, passivation, and corrosion on zinc anodes [20, 268, 269]. This section systematically categorizes and analyzes the current advances in employing these polymers to achieve highly stable zinc anodes from three perspectives: (i) artificial SEI layers, (ii) electrolyte optimization, and (iii) functional separator design.

### 3.1 Artificial SEI Layer

Designing and constructing the SEI layer on zinc anodes is regarded as a direct and highly efficient route to enhancing the cycling stability of zinc anodes and thereby optimizing the electrochemical performance of AZIBs [225, 270, 271]. An ideal SEI layer can not only shield zinc metal anodes from hydrogen evolution side reactions and surface corrosion, but also effectively suppress the growth of dendrites [272]. Nevertheless, most SEI layers based on traditional materials currently still suffer challenges such as complex fabrication processes, high costs, and potential environmental hazards [273, 274]. There is an urgent need to develop novel materials that are more affordable, environmentally friendly, and offer superior performance to achieve the straightforward construction of the SEI layers. Natural macromolecular polymers, characterized by their ubiquity, renewability, environmental friendliness, low cost, abundant functional groups, and high intrinsic mechanical strength, perfectly meet these requirements for constructing novel SEI layers suitable for AZIBs.

Currently, various artificial SEI layers have been constructed based on natural polymers and their derivatives, aiming to effectively enhance the cycling stability of zinc metal anodes. He et al. successfully constructed a robust novel artificial SEI layer on zinc metal anodes by employing chitin molecules rich in polar functional groups (–OH, –NH_2_, –C═O) [108]. Benefiting from the zinc‐philicity and strong electronegativity of the polar groups, this chitin‐based SEI layer firmly adhered to the surface of zinc anodes (Zn@chitin), generating an electrostatic shielding effect that promoted the uniform deposition of Zn^2+^, and thereby inhibiting the formation of zinc dendrites (Figure 5a). Meanwhile, the hydrophobic and water‐insoluble chitin‐based SEI layer effectively prevented the direct contact between active water and zinc anodes, fundamentally avoiding water‐induced side reactions and electrode corrosion.

**FIGURE 5:**
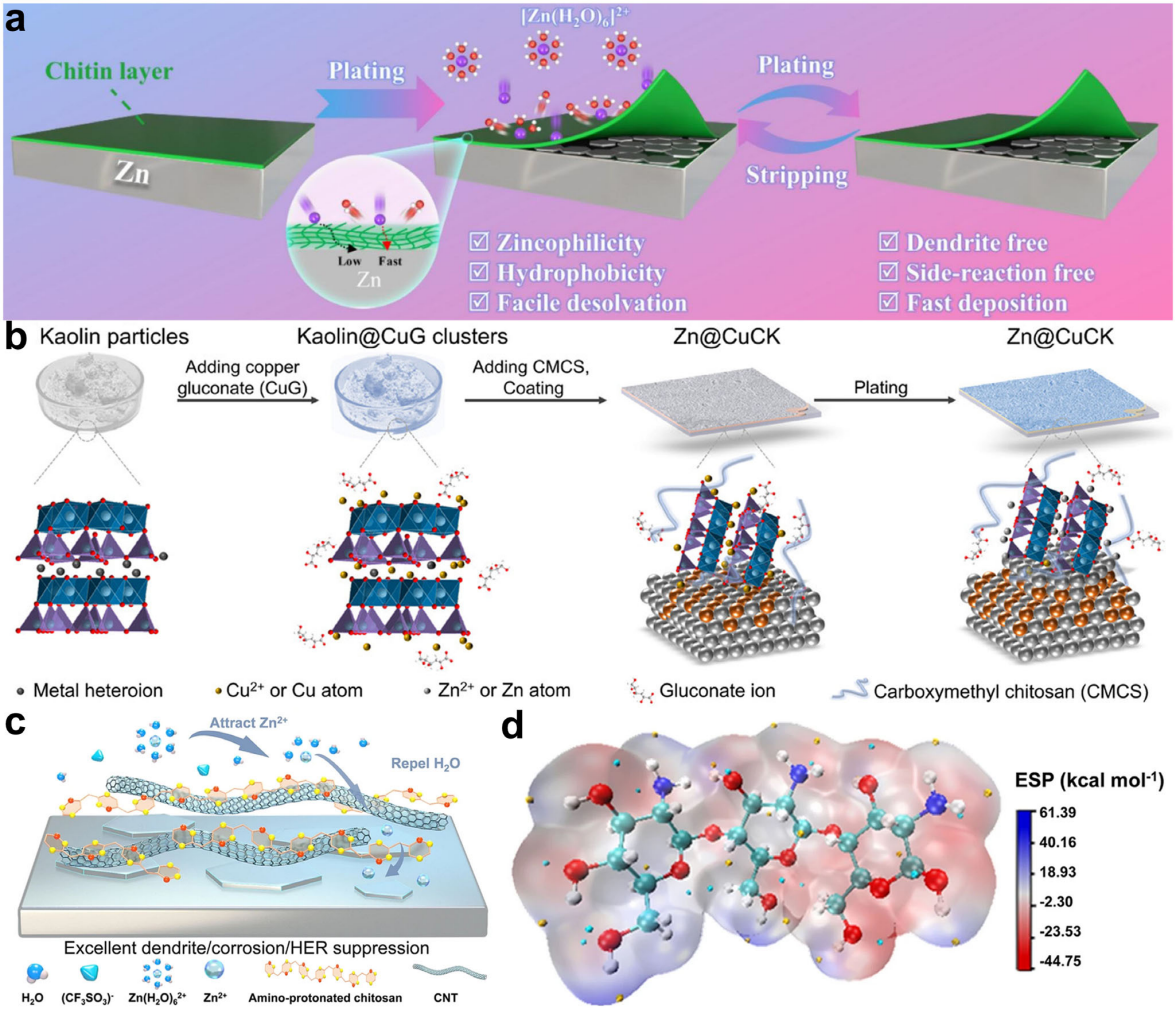
(a) Schematic diagram of the Zn^2+^ deposition process regulated by Zn@chitin anode. Reproduced with permission [108]. Copyright 2024, Elsevier. (b) Schematic illustration of constructing the CuCK SEI layer on zinc anodes. Reproduced with permission [275]. Copyright 2025, Wiley‐VCH. (c) Schematic diagram illustrating the mechanism for optimizing zinc anodes by using the chitosan/CNTs SEI layer. (d) The electrostatic potential (ESP) simulation of chitosan molecule. Reproduced with permission [276]. Copyright 2025, Spring Nature.

Chitosan, as the most representative derivative of chitin, has been further explored in constructing artificial SEI layers suitable for zinc metal anodes. Chen and collaborators employed a simple physical coating method to uniformly distribute a mixed slurry of copper gluconate, kaolin, and carboxymethyl chitosan onto the surface of zinc metal anodes, forming a novel multifunctional CuCK SEI layer (Figure 5b) [275]. The synergistic effect of multiple components not only endowed this novel SEI layer with 3D gradient copper‐zinc nucleation sites, promoting uniform distribution of the interfacial electric field and inhibiting dendrite growth, but also resulted in the SEI layer demonstrating excellent zinc affinity and high adhesion to zinc anodes. In the meantime, carboxymethyl chitosan and kaolin also effectively accelerated the desolvation and transport of zinc ions while suppressing side reactions such as HER, ultimately yielding the highly stable zinc anodes. Similar studies have also constructed SEI protective layers on zinc metal anodes by blade‐coating a composite of chitosan and highly conductive carbon nanotubes (Figure 5c) [276]. The uniformly distributed carbon nanotubes within the constructed chitosan/CNTs SEI layer facilitated even surface electric field distribution across the electrode, while protonated chitosan firmly attached the SEI layer to zinc anodes by virtue of its adhesiveness, and its electron‐rich –OH groups simultaneously optimized the transport of zinc ions (Figure 5d). The two components act synergistically to promote the uniform deposition of zinc ions, thereby suppressing the generation and growth of zinc dendrites. Additionally, Zhou et al. also employed a cross‐linked coating approach to form a carboxymethyl chitosan‐based gel SEI layer on the electrode, enabling effective protection of the zinc anode [277]. This flexible and highly malleable SEI layer featured high ionic conductivity and excellent wettability, and uniformized the surface charge distribution, which facilitated the stable deposition and stripping of zinc ions, thereby significantly enhancing the cycling stability of zinc anodes.

Typically, cellulose and lignin, rich in oxygen‐containing moieties (–COOH and –OH) possess excellent water‐locking and zinc‐philicity, while their oxygen‐containing hydrophilic groups can modulate the solvation sheath of zinc ions, accelerating the migration of zinc ions [124, 264, 278]. For instance, Cheng and colleagues constructed a composite SEI layer on zinc metal anodes using hydroxypropyl cellulose hybridized with zinc trifluoromethanesulfonate, which regulated the transport of zinc ions and improved their desolvation kinetics [114]. Concurrently, the abundant –OH groups on hydroxypropyl cellulose drove the constitution of the interfacial H‐bond networks, reducing the activity of interfacial water and effectively suppressing hydrogen evolution side reactions, consequently stabilizing zinc anodes. Similarly, a protective layer based on amino‐grafted bacterial cellulose (NB‐cellulose) was also employed as an SEI film to stabilize zinc metal anodes [279]. The constructed SEI layer not only shielded zinc anode from aqueous corrosion and evaded side reactions such as HER, but also the ‒NH_2_ groups it contains, which have a stronger binding ability with zinc ions, regulated the solvation structure of zinc ions and induced their uniform deposition, ultimately resulting in a modified zinc anode with good cycling stability. Another research utilized sulfonate grafted cellulose to construct a cholesterol‐structured cellulose nanocrystal (C‐CNC) film that served as an SEI layer capable of homogenizing the surface electric field of zinc electrodes [24]. Simultaneously, the strong coordination effect of polar groups such as sulfonic acid groups on this new type of SEI layer homogenized the distribution of zinc ions at the electrode interface, balancing the transport and reduction of zinc ions at the interface, eventually effectively suppressing zinc dendrites (Figure 6a). Additionally, a highly hydrophilic SEI layer with low interfacial resistance has been reported, constructed by utilizing the polar ester groups in hydrophilic cellulose acetate (CA) to chelate zinc salts [278]. This SEI layer also facilitated rapid diffusion and uniform deposition of zinc ions, effectively restraining the nucleation and growth of zinc dendrites on zinc anodes.

**FIGURE 6:**
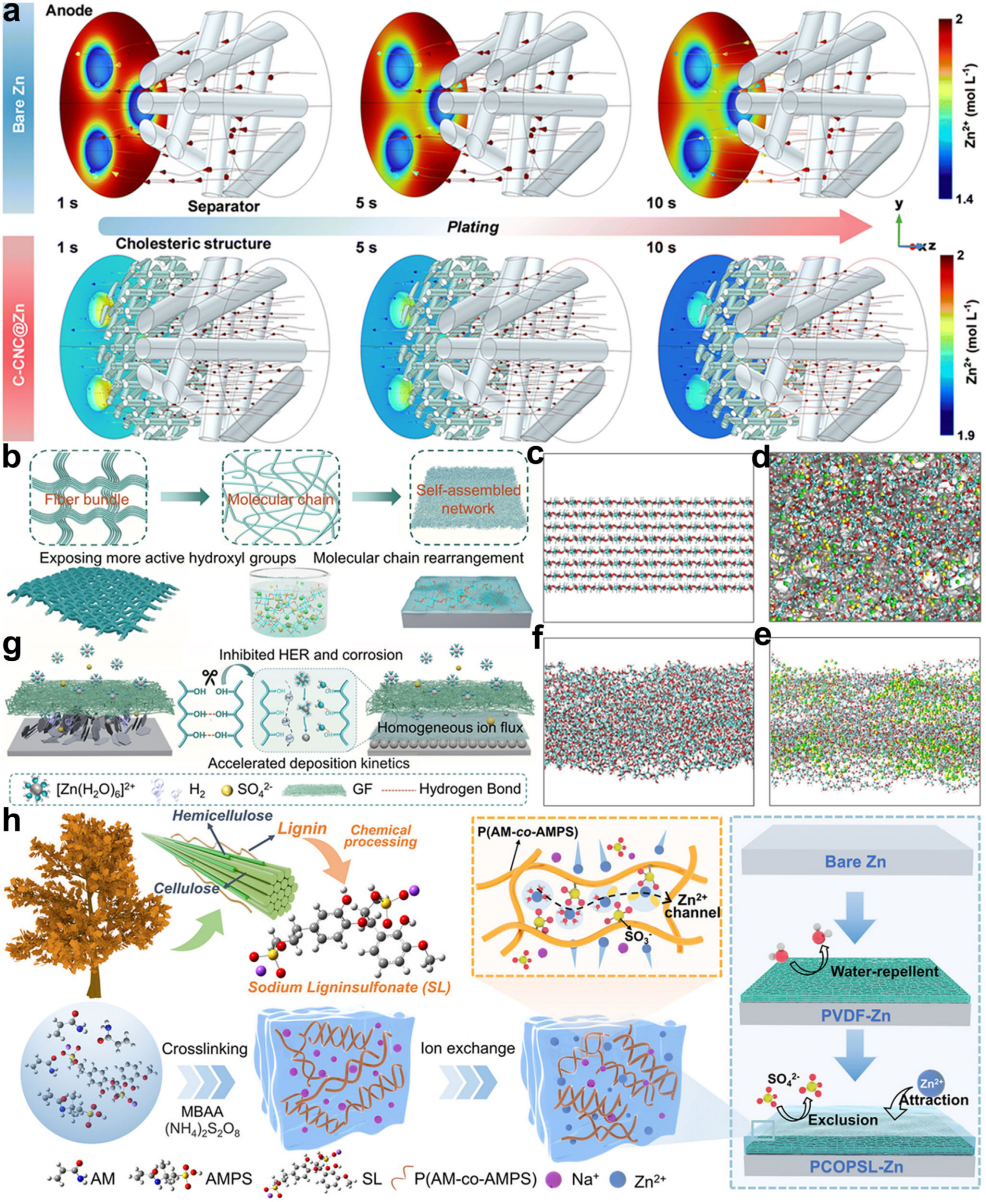
(a) Finite element simulation diagram of the concentration of Zn^2+^ on bare Zn and C‐CNC@Zn anodes. Reproduced with permission [24]. Copyright 2025, The Royal Society of Chemistry. (b) Schematic diagram of the rearrangement process of cellulose molecular chains. (c–f) Molecular dynamics (MD) simulation of the structural evolution of cellulose chains. (g) Schematic illustration of the optimization mechanism of the regenerated cellulose‐based SEI layer for zinc anodes. Reproduced with permission [124]. Copyright 2024, Wiley‐VCH. (h) Schematic illustration of the synthesis of SL and P(AM‐co‐AMPS)/SL, the construction of the P(AM‐co‐AMPS)/SL SEI layer, and the associated ion exchange and transport processes. Reproduced with permission [25]. Copyright 2025, Wiley‐VCH.

In addition to fabricating functionalized cellulose more suitable for constructing SEI layers that can optimize zinc anodes through grafting polar groups, regulating the molecular chain structure of cellulose has also been proven to be an effective strategy [25, 106, 124, 280, 281]. A molecular chain rearrangement strategy was applied to regulate the molecular chain structure of cellulose, yielding regenerated cellulose with lower crystallinity and more exposed active –OH groups (Figure 6b–f) [124]. The artificial SEI layer constructed based on this regenerated cellulose not only accelerated the diffusion of zinc ions by means of the amorphous region and more abundant –OH groups on its interface, promoting the uniform distribution of zinc ions at the electrode interface, but also isolated water molecules and harmful anions, thereby suppressing zinc dendrites and various side reactions (Figure 6g). There are also studies that combine traditional inorganic materials with cellulose to design better SEI protective layers for optimizing zinc metal anodes. Tian et al. developed a novel hybrid hydrogel film by crosslinking and assembling 2D MXene with cellulose nanofibers (CNFs), which effectively improved the stability of zinc anodes as an SEI protective layer [106]. This new type of hybrid SEI layer not only possessed outstanding toughness, high mechanical strength, and ionic conductivity, but also exhibited high affinity for zinc ions, which enabled the uniform distribution of electric fields and stresses across the electrode surface, thereby facilitating the uniform deposition of zinc ions and inhibiting the growth of zinc dendrites. In another research, a CNFs‐graphene (G) composite film assembled from CNFs and G was employed as an SEI layer to regulate the deposition of zinc ions [280]. Within this SEI layer, CNFs rich in hydrophilic functional groups modulated the solvation structure of zinc ions and shielded zinc anodes from the active water molecules. Simultaneously, G with excellent electrical and thermal conductivity within the SEI layer effectively guided the deposition of zinc ions, promoting preferential growth of zinc atoms along the (002) crystal plane, thereby avoiding zinc dendrites.

Furthermore, the SEI layer constructed from lignin or its derivative materials has also been demonstrated to be highly effective in optimizing zinc anodes. Sun and colleagues structured an SEI protective layer based on renewable lignin on zinc anodes via a facile drop‐casting method [282]. The oxygen‐containing functional groups in lignin, such as phenolic hydroxyl groups, enabled its tight and uniform coverage on zinc anodes, forming a dense physical shielding layer that not only promoted the uniform distribution of zinc ions at the electrode interface and inhibited the growth of zinc dendrites, but also effectively prevented the corrosion of polyiodide ions. In a recent study, a polyanionic hydrogel layer (P(AM‐co‐AMPS)/SL) with sodium lignosulfonate (SL) molecules as the active component, constructed using a strategy that combines the scraping‐coating method with further in situ photopolymerization, serving as a dynamic adaptive artificial SEI layer to effectively protect zinc metal anode (Figure 6h) [25]. The SL molecules in this hydrogel SEI layer adsorb onto zinc anodes to cause electrostatic shielding, inducing uniform deposition of zinc ions. Concurrently, these SL molecules, along with the anionic functional groups in the SEI layer that promoted zinc ion migration and a polyvinylidene fluoride layer within the SEI layer that blocked active water, collectively suppressed zinc dendrites and harmful side reactions, enabling long‐term stable cycling of the optimized zinc anodes.

Alginate, endowed with excellent mechanical strength and abundant carboxylate anions is highly attractive for structuring artificial SEI layers to optimize zinc anodes. Currently, a number of researchers are actively exploring alginate‐based SEI layers that can effectively enhance the stability of zinc anodes. Xie et al. established a sodium alginate (SA)‐based SEI layer on the surface of zinc anode, which stabilized the chemical environment at the electrode interface through simple in situ complexation [283]. The carboxylate anions (COO^‒^) in SA interacted with zinc ions, homogenizing the chemical interactions between zinc ions and the electrode interface, and facilitating the uniform diffusion and deposition of zinc ions on zinc anodes. As a result, this SA layer suppressed the zinc dendrites and side reactions, thereby improving the stability of zinc anodes. He and collaborators first spin‐coated alginic acid onto the surface of zinc anodes, and then, during the deposition and stripping of zinc ions, a SA SEI layer with outstanding mechanical properties was generated in situ through the chelation between alginic acid and metal ions (Figure 7a) [284]. Meanwhile, the interaction between alginic acid and zinc ions established the uniform zinc ion diffusion pathways at SEI‐electrode interface, promoting the even distribution of zinc ions across the surface of zinc anodes. Furthermore, this SEI layer modulated the desolvation structure and deposition behavior of zinc ions, facilitated the formation of Zn (002) crystal facets, and thereby resulted in a dense and dendrite‐free zinc deposition layer (Figure 7b).

**FIGURE 7:**
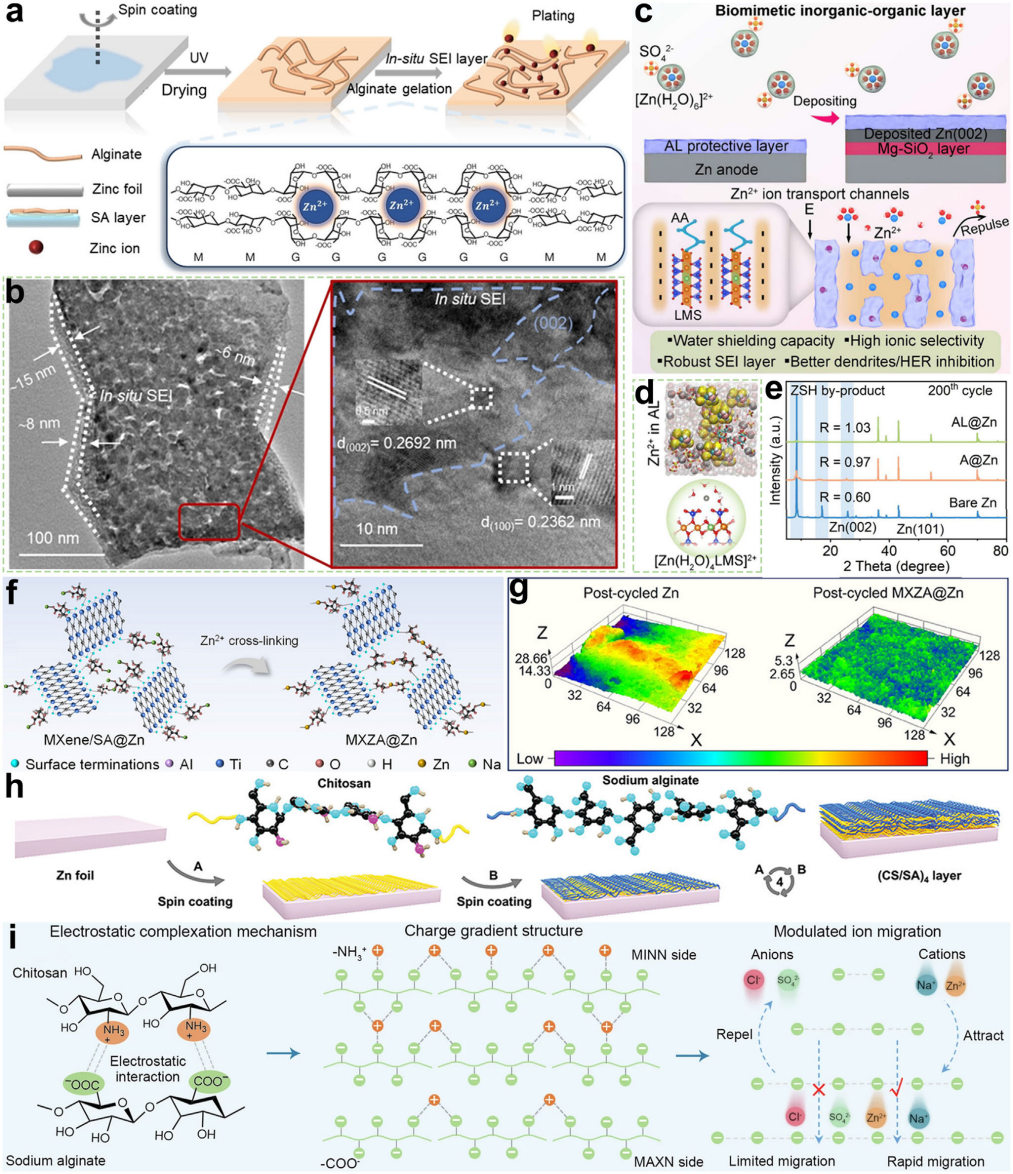
(a) Schematic illustration of in situ construction of SA SEI layer. (b) Transmission electron microscope (TEM) image of zinc anode covered with the SA SEI layer after cycling and corresponding local magnification image. Reproduced with permission [284]. Copyright 2023, Wiley‐VCH. (c) Mechanism diagram of AL SEI layer regulating the deposition of zinc ions. (d) Simulated solvation structure of zinc ions in AL SEI layer. (e) X‐ray Diffraction (XRD) patterns of different zinc anodes after 200 cycles. Reproduced with permission [285]. Copyright 2025, American Chemical Society. (f) Schematic illustration of constructing MXZA SEI layer on zinc anode through the cross‐linking effect of zinc ions. (g) Confocal laser scanning microscope (CLSM) images of bare zinc anode and zinc anode coated with MXZA SEI layer after cycling. Reproduced with permission [287]. Copyright 2025, Wiley‐VCH. (h) Schematic of fabricating the (Chitosan/SA)_4_ SEI layer by sequentially spin‐coating. Reproduced with permission [153]. Copyright 2024, Wiley‐VCH. (i) Schematic diagram of the components, gradient charge structure, construction mechanism of the CG‐Chitosan/SA layer, along with its guided ion transport. Reproduced with permission [288]. Copyright 2025, Spring Nature.

In the past two years, the construction of organic‐inorganic composite SEI layers by hybridizing alginic acid or alginate with inorganic functional materials to improve the electrochemical stability of zinc anodes has garnered increasing attention. Qin and colleagues fabricated the modified zinc anodes covered with the composite SEI layer by uniformly coating a hybrid slurry of alginic acid and lithium magnesium silicate (LMS) onto zinc electrode [285]. Within this alginic acid‐LMS (AL) SEI layer, the rapid ionic pathways formed by alginic acid and LMS modulated the solvation structure of zinc ions, accelerating their transport kinetics (Figure 7c,d). Meanwhile, this SEI layer not only prompted the formation of a zinc‐affinity and robust Mg‐SiO_2_ interface phase at the electrode interface, facilitating the deposition of zinc ions along the Zn (002) facet (Figure 7e), but also reduced surface‐active water molecules on zinc anodes, thereby suppressing water‐induced side reactions and electrode corrosion. Yang et al. developed an enhanced SiO_2_@SA hybrid SEI film by simply scraping a mixed slurry of SiO_2_ and SA, effectively optimizing the interface double electric layer near zinc anodes [286]. In addition, this novel SEI layer promoted the homogenization of zinc ions, effectively isolated SO_4_^2‒^ and reduced the activity of free water molecules. Ultimately, it effectively inhibited zinc dendrites and side reactions, significantly enhancing the stability of zinc anodes.

Notably, several functional inorganic materials (such as molecular sieves and MXene) have also been employed in conjunction with alginates to construct organic‐inorganic hybrid SEI layers. Chen and collaborators constructed a unique hybrid SEI layer with excellent isotropic compatibility and firm adhesion to zinc anodes by confined cross‐linking hollow Zn‐SSZ13 molecular sieves with alginates and further in situ grafting [145]. The alginates in this SEI layer facilitated the uniform deposition of zinc ions, while the Zn‐SSZ13 particles therein inhibited the reactivity of interfacial water. Their synergistic action enabled this novel SEI layer to significantly enhance the electrochemical performance and stability of zinc anodes. Zhang et al. mixed Ti_3_C_2_T_x_ MXene material with organic zinc alginate to form an organic‐inorganic hybrid slurry, which was then coated onto the surface of zinc electrodes and cross‐linked with zinc ions to construct a composite MXZA SEI layer (Figure 7f) [287]. Benefitting from the enhancement of the electrolyte wettability and hydrogen evolution overpotential by zinc alginate, as well as the optimization of electron/ion conduction, inhibition of side reactions and promotion of uniform zinc ion deposition by MXene, this SEI layer effectively suppressed zinc dendrites and improved the cycling stability of zinc anodes (Figure 7g).

Besides, different kinds of natural polymers were also jointly applied to construct functional SEI layers simultaneously, integrating their respective structural and property advantages to improve the electrochemical performance of zinc anodes. In 2022, Mei and collaborators vacuum‐filtered a mixed dispersion of lignin‐containing cellulose nanofibers (LCNFs) and MXene into a composite membrane, which served as an artificial SEI layer to protect zinc anodes [264]. LCNFs exhibited lower porosity compared to pure cellulose, which enhanced the mechanical strength of the constructed SEI layer, thus effectively suppressing zinc dendrites. Meanwhile, the MXene component within the SEI layer facilitated the transport of zinc ions and guided the lateral growth of zinc metal. As a result of these synergistic optimizations, this dual‐functional SEI layer significantly extended the cycling life of zinc anodes. Song et al. constructed a novel SEI protective layer on zinc electrodes by sequentially spin‐coating chitosan and SA, leveraging intra‐ and inter‐layer self‐assembly (Figure 7h) [153]. This gel‐like multilayer (Chitosan/SA)_4_ SEI film is soft and chemically stable, which not only accelerated the desolvation of hydrated zinc ions and enhanced the transport kinetics of zinc ions, but also homogenized the surface electric field of zinc electrode, promoting the uniform deposition of zinc ions. Furthermore, the excellent mechanical strength of this SEI film enabled it to adapt to the volume variations during the depositing/stripping of zinc ions and suppress adverse side reactions and zinc dendrites, consequently enhancing the electrochemical cycling stability of zinc anodes.

Some researchers have also improved the electrochemical stability of zinc anodes by crosslinking a mixed hydrogel composed of carboxymethyl chitosan (CMCHS) and SA onto zinc electrode as an artificial SEI layer [99]. This gel SEI layer not only was rich in reconstructed H‐bond networks, accelerating the desolvation of hydrated zinc ions, but also had strong zinc‐affinity and abundant cross‐linked chains, promoting the ordered migration of zinc ions and their homogenization at the electrode interface, thereby effectively inhibiting side reactions such as HER and zinc dendrites. Yu, Wu, and colleagues fabricated an SEI layer with a charge gradient by sequentially casting SA and chitosan (CG‐Chitosan/SA SEI layer) and utilizing diffusion‐controlled electrostatic complexation (Figure 7i) [288]. This new type of CG‐Chitosan/SA SEI layer, featuring negatively charged regions that progressively intensify toward the zinc electrode was capable of dispersing the anions (chloride ions, SO_4_^2‒^) diffusing towards zinc anodes while accelerating the transport of zinc ions, inhibiting side reactions and electrode corrosion, and promoting the uniform deposition of zinc ions. These multiple protective and optimizing effects enable the SEI‐coated zinc anodes to exhibit outstanding cycling stability in seawater‐based electrolytes.

By reviewing the research advances on natural polymer‐based artificial SEI layers aimed at improving the cycling stability of zinc anodes, this section showcased the ingenious design and construction of artificial SEI layers while conclusively confirming that the structural and surface characteristics of natural polymers conferred on them outstanding superiority in constructing artificial SEI layers with excellent performance.

In a nutshell, the hierarchical architectures such as the chain structures, network frameworks, and H‐bond networks of various natural polymers support the construction of SEI layers, which is the structural basis for SEI layers to have excellent mechanical properties, flexibility, self‐supporting ability, self‐healing capability, and abundant ion channels. Meanwhile, the different surface polar functional groups on the natural polymers endow the SEI layer with the ability to homogenize the electrode interface electric field and drive the desolvation, rapid transport and uniform deposition of Zn^2+^. At present, these existing related studies have achieved preliminary breakthroughs in addressing the challenge of insufficient interface stability of zinc anodes, which leads to unsatisfactory cycle durability for practical applications. Moreover, this research field continues to flourish, holding immense potential for advancing the industrial application of AZIBs. Therefore, summarizing and comparing the construction approaches and electrochemical performances of various reported artificial SEI layers (Table 1), alongside conducting in‐depth analysis of these researches, offers significant reference and guiding value for further advancing this crucial research topic in the future and ultimately achieving the commercial application of zinc anodes.

**TABLE 1:** Construction approaches for natural macromolecular polymer‐based artificial SEI layers and their electrochemical cycling performance.

Artificial SEI layer	Constructing approaches	Cycling performance of symmetric batteries	Cycling stability of full batteries	Refs
Zn@Chitin	Coating	1300 h (20 mA cm^−2^/1 mAh cm^−2^)	1500 cycles/78.8 % (5A g^−1^)	[108]
Zn@CuCK	Coating	3600 h (2 mA cm^−2^/1 mAh cm^−2^)	12000 cycles/83.5% (5A g^−1^)	[275]
Chitosan/CNTs @Zn	Coating	2000 h (2 mA cm^−2^/1 mAh cm^−2^)	3000 cycles/−(1A g^−1^)	[276]
Zn‐carboxymethyl chitosan gel	Coating	2000 h (1.25 mA cm^−2^/‐)	—	[277]
Hydroxypropyl cellulose@Zn	Coating	9500 h (0.5 mA cm^−2^/0.25 mAh cm^−2^)	4000 cycles/89.1% (5A g^−1^)	[114]
NB‐cellulose@Zn	Coating	1200 h (2 mA cm^−2^/1 mAh cm^−2^)	1000 cycles/− (1A g^−1^)	[279]
C‐CNC@Zn	vacuum filtration	1000 h (1 mA cm^−2^/1 mAh cm^−2^)	1000 cycles/92.0% (0.5 c)	[24]
CA‐Zn(CF_3_SO_3_)_2_ @Zn	Coating	2800 h (1 mA cm^−2^/1 mAh cm^−2^)	2000 cycles/110% (1A g^−1^)	[278]
Regenerated cellulose@Zn	Coating	8000 h (10 mA cm^−2^/1 mAh cm^−2^)	5000 cycles/76.1% (5A g^−1^)	[124]
MXene‐CNF|Zn	vacuum filtration	2700 h (1 mA cm^−2^/1 mAh cm^−2^)	2000 cycles/−(3A g^−1^)	[106]
CNFs‐G/Zn	Drying of dispersion solution	5500 h (0.25 mA cm^−2^/0.5 mAh cm^−2^)	5000 cycles/87.8% (5 c)	[280]
Zn@lignin	Coating	650 h (2 mA cm^−2^/2 mAh cm^−2^)	35000 cycles/99.7% (6A g^−1^)	[282]
P(AM‐co‐AMPS)/SL‐Zn	Coating	9000 h (0.1 mA cm^−2^/0.1 mAh cm^−2^)	1000 cycles/70.12% (5A g^−1^)	[25]
SA@Zn	in‐situ complexation	1200 h (1 mA cm^−2^/1 mAh cm^−2^)	1300 cycles/−(2A g^−1^)	[283]
SA‐coated Zn	Coating	1000 h (0.5 mA cm^−2^/0.5 mAh cm^−2^)	3000 cycles/87.1% (5A g^−1^)	[284]
Alginic acid/LMS‐Zn	Coating	5500 h (1 mA cm^−2^/1 mAh cm^−2^)	8000 cycles/− (5A g^−1^)	[285]
Zn@SiO_3_‐SA	Coating	3000 h (2 mA cm^−2^/1 mAh cm^−2^)	1500 cycles/−(7.5A g^−1^)	[286]
Zn‐SSZ13/ carrageenan@Zn	Coating	1200 h (10 mA cm^−2^/1 mAh cm^−2^)	600 cycles/76.3% (1A g^−1^)	[145]
MXZA@Zn	Coating	2500 h (2 mA cm^−2^/2 mAh cm^−2^)	1000 cycles/98.4% (4A g^−1^)	[287]
Zn|LCNFs‐Mxene	Drying of dispersion solution	800 h (0.5 mA cm^−2^/0.5 mAh cm^−2^)	1000 cycles/90% (1A g^−1^)	[264]
(Chitosan/SA)_4_‐Zn	Self‐assembly	6500 h (1 mA cm^−2^/1 mAh cm^−2^)	14000 cycles/83.6% (5A g^−1^)	[153]
CMCHS/SA‐Zn	In situ Gelation	2860 h (1 mA cm^−2^/1 mAh cm^−2^)	60,000 cycles/‐(5A g^−1^)	[99]
CG‐Chitosan/SA @Zn	Coating	1300 h (1 mA cm^−2^/1 mAh cm^−2^)	5000 cycles/51% (5A g^−1^)	[288]

### 3.2 Electrolyte Optimization

At present, optimizing electrolytes using natural macromolecular polymers to improve the electrochemical cycling stability of zinc anodes has received increasing attention, and some previous related progress has laid a solid foundation and provided strong reference for the subsequent development in this field [180, 289]. These researches mainly optimize the electrolytes to achieve the improvement of zinc anodes through two main pathways: (i) introducing natural polymers as additives into aqueous electrolytes, and (ii) constructing functionalized gel electrolytes using the polymers. The reason these two approaches dominate is that they can suppress the inevitable spontaneous side reactions on the surface of the zinc electrodes within aqueous electrolyte systems by regulating the solvation structure of zinc ions and their surface distribution on the zinc electrode, and by blocking active water molecules from eroding the zinc electrode, and reducing the activity of interfacial water [21, 290] Benefiting from abundant functional groups (–OH, –COOH, –NH_2_, etc.), exceptional mechanical strength, environmental friendliness and renewability, natural polymer‐based additives, and gel electrolytes have yielded a series of achievements in optimizing the electrochemical performance of zinc anodes, delivering a green and eco‐friendly pathway for the sustainable development of AZIBs.

#### 3.2.1 Electrolyte Additives

Applying functional additives to the electrolyte of AZIBs is regarded as a cost‐effective, facile, and readily scalable strategy for optimizing the electrochemical performance of zinc anodes [205, 291, 292, 293]. Typically, introducing such additives can inhibit side reactions like HER, interfacial corrosion, and zinc dendrites by regulating the composition and properties of the electrolyte, optimizing the solvation structure and transport of hydrated zinc ions, reducing interfacial water activity, and forming a protective interfacial layer, thereby effectively improving the cycling durability of zinc anodes [294, 295, 296, 297, 298, 299]. Because of their advantages such as multifunctionality, eco‐friendliness, and wide availability, natural polymers are increasingly being engineered as functionalized electrolyte additives for stabilizing zinc anodes [162, 250, 290, 300, 301].

When such natural polymers are incorporated into the electrolyte of AZIBs, their abundant hydrophilic groups can actively participate in the formation of the solvation shell of zinc ions, optimizing the solvation structure and transport kinetics of zinc ions, while also reconstructing the hydrogen‐bond network within the electrolyte to suppress the reactivity of interfacial water molecules [21, 23, 96, 255, 302, 303]. For instance, cellulose nanocrystals (CNCs) extracted from wood and rich in electronegative oxygen‐containing groups such as ‒OH and ‒COOH were applied as additives to restructure the solvation environment of the electrolyte, reducing the number of water molecules coordinated to Zn^2+^ and thereby accelerating the transport kinetics of Zn^2+^ (Figure 8a–d). Meanwhile, owing to their intrinsic self‐assembly capability, these CNC additives could spontaneously form a dynamic, self‐healing interfacial protective layer on zinc anodes, facilitating uniform deposition of zinc ions and effectively suppressing interfacial side reactions and zinc dendrite growth [21]. In addition, a type of chitin nanocrystals (ChNCs) extracted from crab shells is served as a colloidal electrolyte additive, enabling long‐term stable cycling of zinc anodes (Figure 8e) [162]. These colloidal additives simultaneously optimized both the electrolyte bulk environment and the electrode interface chemistry, and their cyclic shuttling between the electrolyte and the electrode interface ensured the long‐term dynamic effectiveness of these functions (Figure 8f). In particular, during cycling, ChNCs migrating to zinc anodes could repeatedly self‐assemble into a functionalized protective layer to resist internally induced mechanical damage and external physical destruction, endowing zinc anodes with self‐healing capability and thereby ensuring their excellent cycling performance (Figure 8g). Furthermore, Niu and colleagues employed CMCHS, a water‐soluble zwitterionic polymer derived from chitosan, as an electrolyte additive for AZIBs, leveraging its anti‐polyelectrolyte effect to enhance the cycling stability of zinc anodes (Figure 8h) [304]. The anti‐polyelectrolyte effect of CMCHS was triggered by the concentration gradient of zinc ions near zinc anodes, where CMCHS molecules self‐assembled at high zinc ion concentrations into cross‐linked ionic channels that could homogenize the distribution of ions and electrons on the surface of zinc anodes and reduce interfacial water activity, thereby inhibiting dendrite growth and water‐induced side reactions.

**FIGURE 8:**
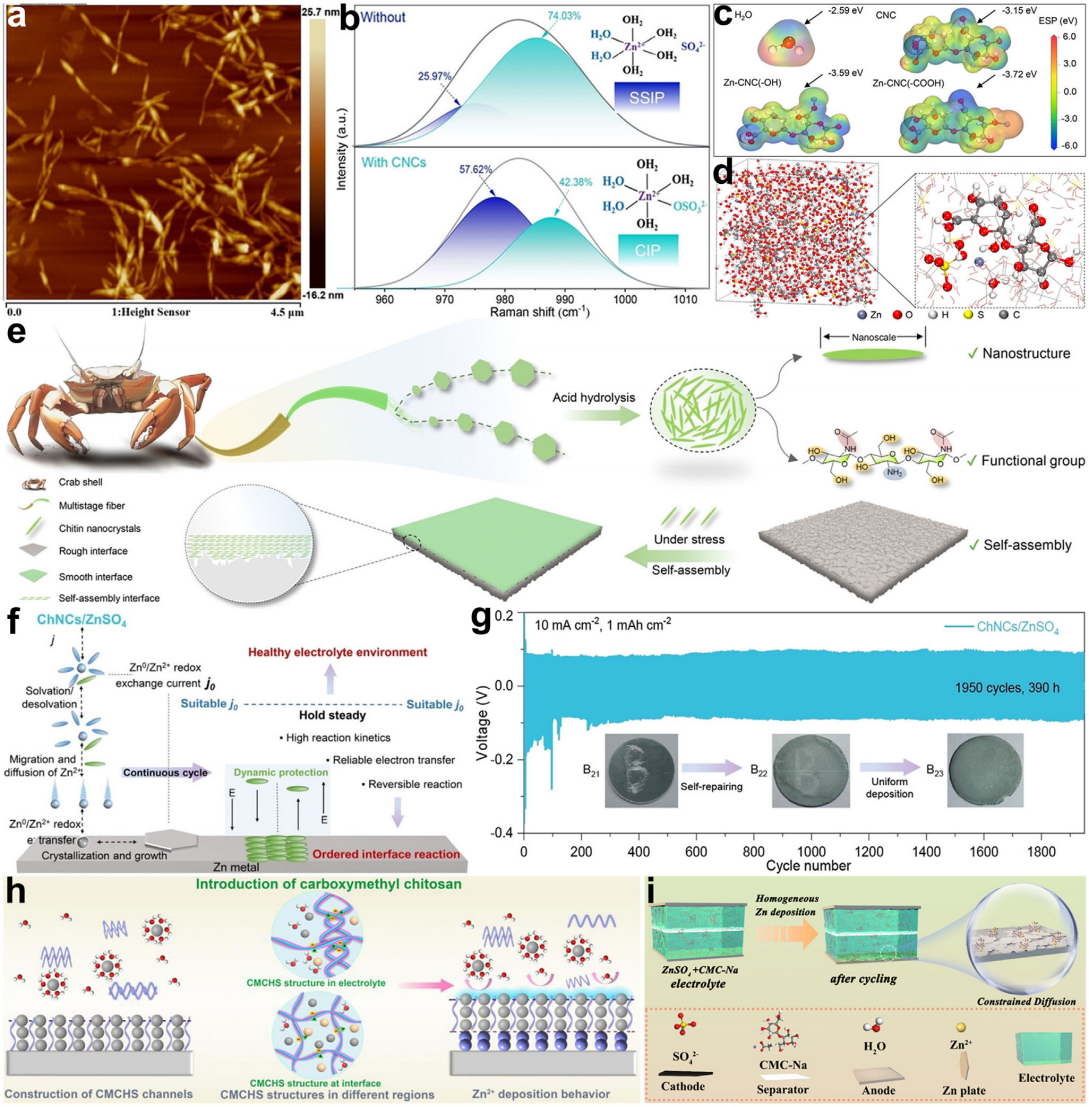
(a) The atomic force microscopy (AFM) image of CNCs. (b) Raman fitting spectra of ν‐SO_4_^2−^ in different electrolytes. (c) ESP simulations of H_2_O, CNC, Zn‐CNC‐OH, and Zn‐CNC‐COOH. (d) MD simulation of the ZnSO_4_ (ZSO) electrolyte containing CNC and corresponding local enlarged structure. Reproduced with permission [21]. Copyright 2024, Wiley‐VCH. (e) Schematic diagram of the preparation and structure/property‐oriented optimization mechanism of ChNCs. (f) Schematic depiction showing the variation of exchange current density (*j*_0_) in ZSO electrolyte with ChNCs. (g) The cycling performance of engraved zinc anodes in the Zn||Zn symmetrical batteries using ZSO/ChNCs electrolyte. Reproduced with permission [162]. Copyright 2025, Wiley‐VCH. (h) Mechanism diagram of the anti‐polyelectrolyte effect induced by CMCHS regulating the deposition behavior of zinc ions. Reproduced with permission [304]. Copyright 2025, American Chemical Society. (i) Mechanism diagram of the optimization of zinc anode by CMC‐Na electrolyte additives. Reproduced with permission [290]. Copyright 2024, Elsevier.

In addition to leveraging the regulatory effect of such functional natural polymers alone, combining them with the strong charge repulsion effect of metal ions to achieve synergistic optimization is also an important strategy for constructing highly stable zinc anodes. When the inexpensive carboxymethyl cellulose sodium (CMC‐Na) was incorporated into the electrolyte of AZIBs as an additive, the negatively charged CMC chains released upon ionization regulated the solvation structure of zinc ions and optimized the hydrogen‐bond network of the electrolyte, while the released sodium ions formed an electrostatic protective layer at the electrode interface, suppressing differences in the surface electric field on the electrode [290]. The synergy of these effects promoted the uniform deposition of zinc ions, significantly enhancing the cycling performance of zinc anodes (Figure 8i).

In order to further develop natural polymer‐based electrolyte additives with better improvement effects on zinc anodes, natural polymers were functionalized through strategies including chemical grafting and protonation. Zhou et al. successfully fabricated an amphoteric carboxyethyl quaternized cellulose (CEQC) via stepwise grafting, achieving controllable regulation of the number of both positively and negatively charged groups along the molecular chains of the synthetic products (Figure 9a) [250]. This new type of cellulose‐based polymer, as an additive could not only effectively regulate the solvation structure of zinc ions, promoting their rapid transport and uniform deposition, but also adsorb onto zinc anodes through positively charged groups to form a protective layer that isolated interfacial water, thereby suppressing zinc dendrites and harmful side reactions (Figure 9b,c). Benefitting from these, Zn||Zn symmetric batteries incorporating this additive demonstrated a cycle life exceeding 900 h at high current densities and deposition capacities. Similarly, cellulose levulinate esters with high water solubility were synthesized using microcrystalline cellulose as the raw material and α‐angelica lactone as the derivatizing monomer, which were utilized as an electrolyte additive to enhance the stability of zinc anodes [255]. The synergistic action of abundant functional groups on these cellulose‐derived polymer additives accelerated the desolvation of zinc ions, optimizing their transport kinetics and deposition uniformity. Concurrently, these polymer molecules adsorbed onto the surface of zinc anodes and in situ generated a protective layer that blocked the corrosion of interfacial water and inhibited dendrite formation, thus effectively enhancing the electrochemical performance of zinc anodes.

**FIGURE 9:**
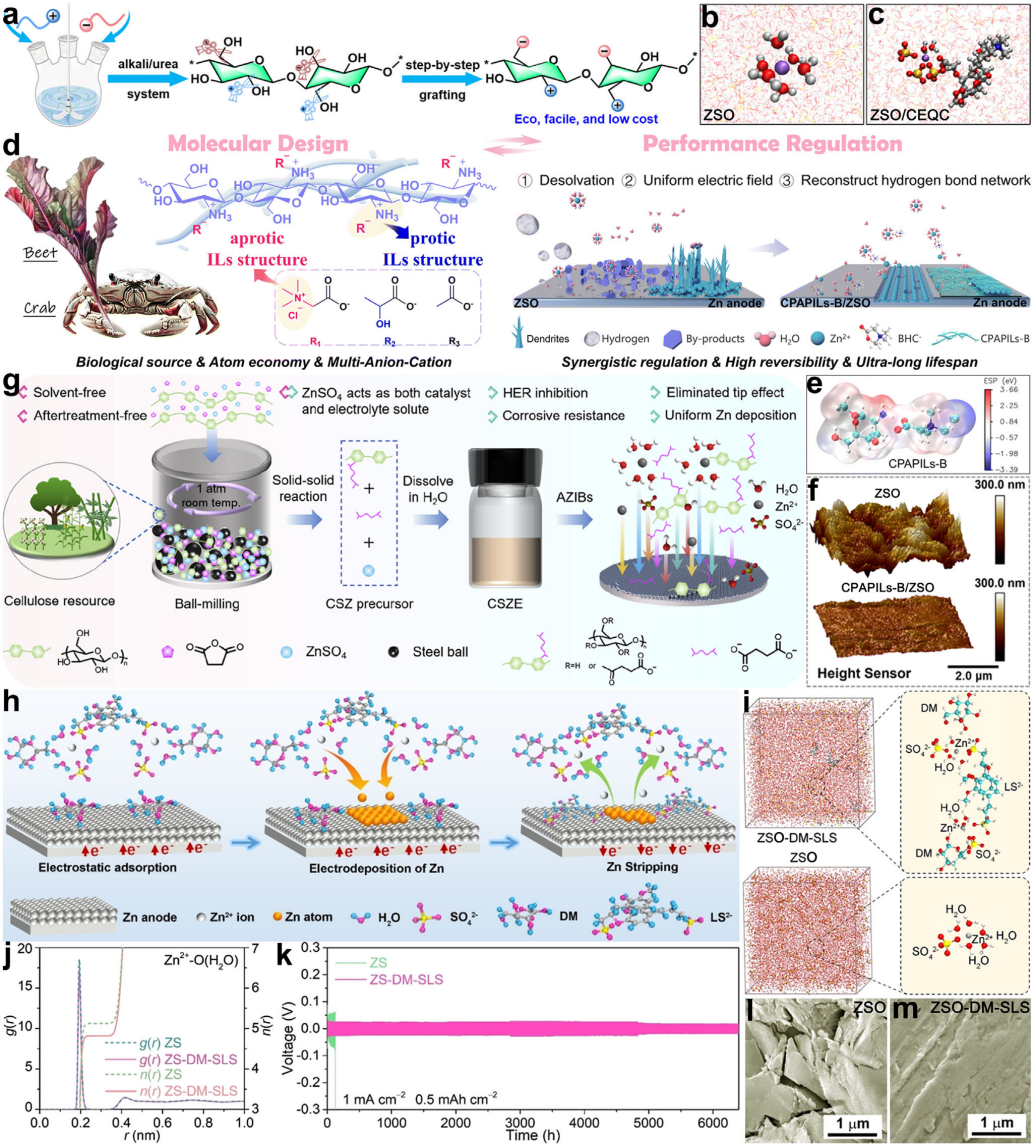
(a) Schematic depiction of the synthesis of CEQC. (b, c) MD simulation snapshots of the solvation structure of zinc ions in ZSO and ZSO/CEQC electrolytes. Reproduced with permission [250]. Copyright 2025, American Chemical Society. (d) Schematic diagram of the preparation of CPAPILs‐B and its regulatory mechanism on zinc anodes. (e) ESP simulation of CPAPILs‐B. (f) AFM images of zinc anodes after cycling in ZSO and CPAPILs‐B/ZSO electrolytes. Reproduced with permission [23]. Copyright 2025, The Royal Society of Chemistry. (g) Diagram of the preparation of CSZE and its application in AZIBs. Reproduced with permission [267]. Copyright 2025, Elsevier. (h) Schematic depiction of SLS and DM electrolyte additives synergistically optimizing the deposition and stripping processes of zinc ions. (i) MD simulations of different electrolytes and corresponding coordination atomic structures. (j) Radial distribution function (RDF) of Zn^2+^−O and coordination numbers of Zn^2+^ with H_2_O in two different electrolytes. (k) The long‐cycle performances of Zn||Zn symmetric batteries with different electrolytes. (l, m) Scanning electron microscope (SEM) images of zinc anodes after cycling in different electrolytes. Reproduced with permission [305]. Copyright 2024, Wiley‐VCH.

Besides, one kind of betaine hydrochloride derived chitosan‐based poly(aprotic/protonic ionic liquid)s (CPAPILs‐B) was synthesized and employed as an electrolyte additive, which improved the electrochemical stability of zinc metal anodes through multi‐anion‐cation interactions with the electrolyte (Figure 9d,e) [23]. The abundant COO^‒^ and chloride ions on these CPAPILs‐B adjusted the solvation structure of zinc ions and reconstructed the hydrogen‐bond network in the electrolyte, thereby optimizing the transport of zinc ions and reducing the activity of interfacial water. And a large amount of quaternary ammonium cations and protonated amines dissociated from CPAPILs‐B anchored and in situ constructed an interfacial shielded layer, promoting uniform distribution of zinc ions and the electric field at the electrode interface. As a result, these synergistic effects of multi‐anions and cations enabled uniform deposition of zinc ions, preventing zinc dendrites (Figure 9f).

Except as chemical grafting and synthesis, a solvent‐free mechanical ball‐milling method has also been successfully applied to develop novel natural polymer‐based electrolyte additives. A mixture of succinic anhydride, microcrystalline cellulose, and zinc sulfate was ball‐milled and then dissolved in water to fabricate a zinc sulfate electrolyte containing two additives cellulose succinate ester and succinic acid (CSZE), which was applied to optimize AZIBs (Figure 9g). The synergistic action of cellulose succinate ester and succinic acid optimized the transport and deposition of zinc ions while adsorbing onto zinc anode to form a protective layer that inhibited the erosion of interfacial water, thereby restraining zinc dendrites and corrosion side reactions [267]. Coincidentally, the superiority of the combined regulation by the dual additives had been verified even earlier. Jiang et al. simultaneously incorporated trace amounts of sodium lignosulfonate (SLS) and D‐mannose (DM) as additives into the zinc sulphate electrolyte, achieving significant improvements to zinc anodes [305]. Research revealed that due to the differing polarities of LS ions and DM, their adsorption energies onto the electrode vary with the positive or negative charge distribution on the electrode surface, allowing them to alternatively and parallelly anchor to the stripping and deposition electrodes (Figure 9h). These dynamically alternating adsorbed additive molecules assembled into reversible dynamic protective layer on the surfaces of the stripping and deposition electrode, enhancing zinc ion transport and deposition kinetics, facilitating the uniform deposition of zinc ions, and thereby inhibiting the growth of zinc dendrites. Moreover, the LS ions and DM molecules desorbed from the electrodes effectively altered the solvation structure of zinc ions, synergistically accelerating their desolvation efficiency and promoting 3D diffusion of zinc ions at the electrode interface, which reduced the activity of interfacial water and further suppressed water‐induced side reactions (Figure 9i,j). Benefiting from these synergistic effects of the dual additives, the assembled Zn||Zn symmetric batteries maintained stable cycling for over 6400 h under 1 mA cm^‒1^ and 0.5 mAh cm^‒2^, and the surface of zinc anodes after cycling was significantly flatter compared to that of the cycled zinc anodes in the symmetric battery without additives (Figure 9k–m).

To mitigate side reactions triggered by both free water and solvated water in aqueous electrolytes, researchers have not only simply utilized additives but also combined them with the construction of deep eutectic electrolytes to regulate the content and activity of water. Such deep eutectic electrolytes are low‐melting mixtures formed through intermolecular interactions such as hydrogen bonding, Lewis acid‐base coordination, and van der Waals forces. By optimizing composition ratios and intermolecular interactions, deep eutectic electrolytes can achieve high ionic conductivity while also offering advantages such as simple preparation, low cost, and environmental friendliness [306, 307, 308]. Yuan et al. developed a deep‐eutectic electrolyte based on ZnCl_2_‐H_2_O utilizing cellulose as a complexing additive, which effectively elevated the cycling durability of zinc anodes [306]. In the study, it was confirmed that the cellulose‐mediated intermolecular interactions effectively suppressed the activity of water, substantially expanding the stable electrochemical window of the deep eutectic electrolyte and markedly enhancing the transference number of zinc ions within this system. Concurrently, an artificial SEI layer predominantly composed of organic matter and incorporating cellulose‐Zn^2+^ complexes was successfully constructed on the surface of the zinc anode, significantly improving the corrosion resistance and electrochemical performance of the zinc anode. As a result, the assembled Zn||Zn symmetric batteries with this deep eutectic electrolyte containing cellulose delivered highly stable stripping/plating even at high current densities, achieving a remarkable depth‐of‐discharge of 85 %.

Natural macromolecular polymer‐based additives excel in modulating the solvation structures of Zn^2+^ and improving the stability of the electrode interface [21, 23, 96, 305]. Nevertheless, these polymers still face some hurdles in promoting the practical application of AZIBs, such as limited aqueous solubility, monofunctional regulation, and poor compatibility with other electrolyte components [21, 96, 309]. To overcome these challenges, future efforts are recommended to focus on (i) developing composite additive systems that combine multiple natural polymer species or integrating natural polymers with other co‐additives to exploit synergistic effects, and (ii) extracting or chemically modifying natural polymer‐derived compounds rich in zinc‐philic functional groups to achieve optimal multifunctionality and optimized synergistic effects. Moreover, it should be noted that for additive systems, especially polymer‐based additives, appropriately increasing the concentration is conducive to enhancing solvation structure regulation of Zn^2+^ achieving more complete interfacial adsorption. However, excessive dosage may lead to excessively high viscosity of the electrolyte and overly thick interfacial layers, significantly aggravating the concentration polarization of Zn^2+^, hindering the rapid transport and ordered deposition of Zn^2+^, and ultimately compromising the ring stability of zinc anodes. Therefore, the optimization of the dosage of natural polymer‐based additives should also receive more attention in future research.

#### 3.2.2 Gel Electrolytes

Gel electrolytes demonstrate significant superiority in eliminating side reactions such as HER caused by active water in AZIBs by replacing traditional aqueous electrolytes, which reduce water content and the activity of water [310, 311]. This class of electrolyte integrates the high ionic conductivity and fluidity of aqueous electrolytes with the excellent mechanical properties and safety of solid‐state electrolytes, and therefore is increasingly being developed and applied to optimize the electrochemical performance of AZIBs [91]. However, conventional gel electrolytes usually rely on flammable organic solvents to be constructed and are composed of non‐degradable synthetic polymers, posing safety hazards and environmental pollution risks during their synthesis and application [312, 313]. In contrast, natural macromolecular polymer‐derived gel electrolytes are inherently green: they are lower in cost, biodegradable, renewable, and environmentally benign. More importantly, the abundant polar functional groups distributed on the macromolecular polymers not only confer strong affinity for Zn^2+^ and superior ionic conductivity upon these electrolytes, but also form stable hydrogen‐bond networks with water molecules, inhibiting the transition of water to ice, thereby enabling this type of electrolyte to exhibit outstanding antifreezing performance [91, 289, 314]. Moreover, these polymers inherently possess excellent mechanical strength, film‐forming ability and flexibility, which leads to the gel electrolyte they serve as the framework for construction having outstanding mechanical properties and remarkable flexibility. Meanwhile, the flexible molecular chains of these polymers can move moderately and flexibly in the gel electrolyte, promoting the repeated breaking and regeneration of dynamic chemical bonds (such as H‐bonds, etc.) within the gel, and endowing the natural polymer‐based gel electrolyte with excellent self‐healing capabilities. Owing to the aforementioned numerous advantages, natural macromolecular polymer‐based gel electrolytes hold significant potential for optimizing AZIBs, especially flexible wearable zinc‐ion batteries (ZIBs) and quasi‐solid‐state ZIBs, and related research has progressively become a hot topic in recent years [107, 315].

Typically, strategies such as chemical/physical crosslinking, polymer grafting, ion exchange, and electrogelation are employed to construct natural macromolecular polymer‐based gel electrolytes suitable for AZIBs [289, 316, 317]. Chemical crosslinking exploits the abundant functional groups on natural macromolecular polymers to form robust hydrogen or covalent bonds with other components, endowing the generated gel electrolytes with good mechanical integrity, whereas physical crosslinking offers a facile manufacturing process and enables the synthesized gel electrolytes to have a certain degree of self‐healing capability. Through sequential physical and chemical crosslinking, Zhou and colleagues successfully constructed a kind of dual‐crosslinked cellulose‐based hydrogel exhibiting outstanding mechanical properties, employing it as an electrolyte for AZIBs [107]. Benefiting from the self‐assembly and interlaced entanglement of cellulose chains, these synthesized cellulose‐based hydrogel electrolytes possess a unique network structure, not only demonstrating high toughness and elasticity but also containing abundant ionic transport channels internally, which facilitated the rapid transport of zinc ions while suppressing the growth of zinc dendrites. Moreover, the numerous ‒OH on the cellulose chains endowed these gel electrolytes with outstanding hydrophilicity and zinc affinity, promoting the desolvation of zinc ions and reducing the activity of interfacial water molecules, thereby effectively inhibiting interfacial side reactions.

Peng et al. recently found that the dual‐crosslinked cellulose‐based hydrogels (DCH) obtained through chemical and physical double crosslinking exhibited significantly improved mechanical properties and ionic conductivity after being soaked in sulfate ion solution (DCH‐S) [118]. Sulfate ions displayed a significant anion‐enhancing effect on both the mechanical properties and ionic conductivity of the DCH electrolytes, which was attributed to their ability to trigger the reconstruction of the hydrogen‐bond network within the DCH (breaking the original H‐bonds between water molecules and cellulose to facilitate the formation of H‐bonds between cellulose chains, and simultaneously forming strong anion‐hydrogen bonds with bound water), causing some of the free water to be expelled from the hydrogel system and evolving into a more uniform and dense porous cellulose network (Figure 10a–c). In particular, the uniform cellulose network with abundant interchain H‐bonds, coupled with rich anion‐hydrogen bonds, synergistically accelerated the desolvation and transport kinetics of zinc ions, while promoting uniform deposition of zinc ions on zinc anodes (Figure 10d,e). Consequently, the application of these DCH‐S electrolytes effectively suppressed zinc dendrites and harmful side reactions, significantly enhancing the cycling stability of zinc anodes. In addition, Huang and collaborators combined chemical crosslinking with in situ derivatization to develop a carboxylic acid‐functionalized cellulose/PVA containing Zn^2+^ gel (COOH‐f‐CellPZ gel) electrolyte [318]. These optimized cellulose‐based gel electrolytes not only exhibited excellent mechanical properties but also had a uniform cellulose network and high ionic conductivity, facilitating the efficient transport and uniform deposition of zinc ions, thereby suppressing harmful interfacial side reactions and zinc dendrites.

**FIGURE 10:**
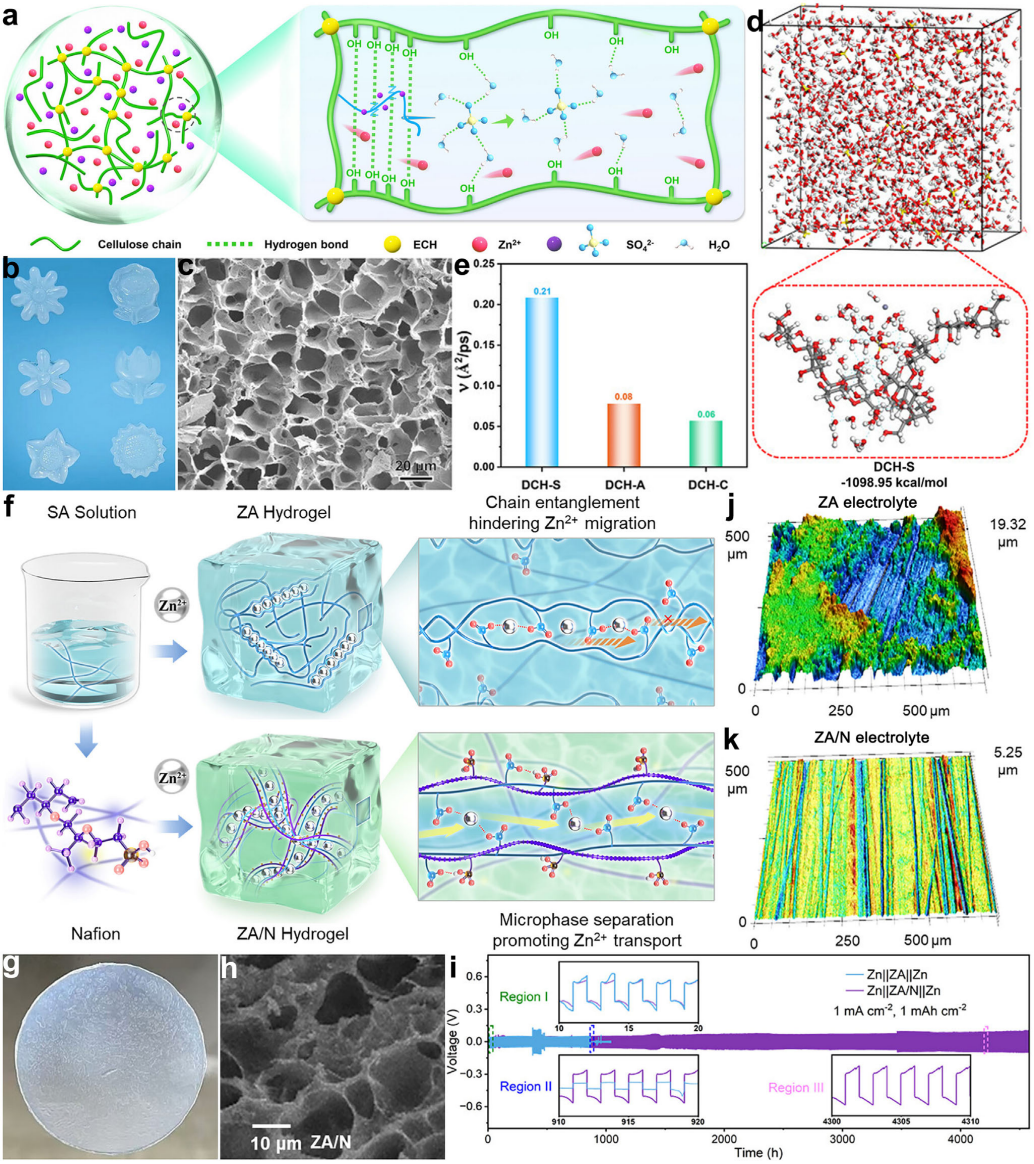
(a) Illustration of DCH‐S electrolyte regulating the transport behavior of zinc ions. (b) Digital photos of DCH‐S electrolytes in different shapes. (c) SEM characterization of DCH‐S electrolyte after freeze‐drying. (d) MD calculation result of DCH‐S electrolyte and the corresponding binding energy. (e) The average transfer rate of zinc ions in different DCH‐based electrolytes. Reproduced with permission [118]. Copyright 2025, Wiley‐VCH. (f) Diagram of the construction of ZA and ZA/N gel electrolytes. (g) Digital photo of ZA/N gel electrolyte. (h) SEM characterization of ZA/N gel electrolyte after freeze‐drying. (i) The long‐term cyclic stabilities of the Zn||Zn symmetric batteries using ZA and ZA/N gel electrolytes. (j, k) CLSM images of zinc anodes after cycling in Zn||Zn symmetric batteries using ZA and ZA/N gel electrolytes. Reproduced with permission [134]. Copyright 2025, Wiley‐VCH.

Beyond cellulose‐based gel electrolytes, alginate‐based and chitosan‐based gel electrolytes have also been developed to optimize the stability of zinc anodes, thereby enhancing the electrochemical performance of AZIBs [109, 134, 159, 315, 319]. For example, Hu et al. have fabricated a chitosan‐based gel coordinated with zinc ions (chitosan‐Zn), which possesses high ionic conductivity for zinc ions and excellent mechanical properties [159]. This synthesized chitosan‐based gel electrolyte, whose water content can be effectively regulated, performed best in improving the stability of zinc anodes when its water content is 57%. The research has confirmed that the chitosan‐based gel electrolyte with a water content of 57% not only accelerated the desolvation and efficient transport of zinc ions but also facilitated the uniform deposition of zinc ions to eliminate zinc dendrites. Jiang and colleagues constructed a flexible alginate‐based hydrogel (SA‐Zn(Ac)_2_ hydrogel) electrolyte to optimize the electrochemical stability of zinc anodes [315]. Since the highly hydrophilic gel framework traps most of the water molecules within the hydrogel, this synthesized SA‐Zn(Ac)_2_ hydrogel electrolyte reduced the contact between free water and zinc anodes, effectively inhibiting water‐induced corrosion reactions. At the same time, the high ionic conductivity and the embedded alginate chain network enabled this hydrogel electrolyte to facilitate rapid zinc ion transport while also homogenizing their distribution at the electrode interface, thereby inhibiting zinc dendrites caused by the aggregated deposition of zinc ions.

Recently, a group of researchers have developed a Nafion‐induced regulation strategy to overcome the problem that the severe self‐entanglement of internal polymer chains in traditional alginate‐based gel electrolytes hinders the formation of continuous ion transport channels, which restricts the high‐speed conduction of zinc ions [134]. Specifically, the incorporation of Nafion triggered the formation of hydrophilic/hydrophobic microdomains in the alginate‐based hydrogel coordinated with zinc ions (ZA hydrogel), unraveling the entanglement of polymer chains and establishing continuous zinc ion transport channels (Figure 10f–h). This Nafion‐modified ZA hydrogel (ZA/N hydrogel), serving as the electrolyte in AZIBs, ensured the directional and efficient transport of zinc ions while promoting their uniform distribution at the electrode interface, thereby significantly suppressing side reactions and zinc dendrites. Consequently, the assembled Zn||Zn symmetric batteries with the ZA/N hydrogel electrolyte stably cycled at 1 mA cm^‒2^ for more than 4600 h, and the corresponding zinc anode after cycling exhibited a flatter surface (Figure 10i–k).

Additionally, some researchers have attempted to chemically or physically crosslink inorganic functional materials or synthetic polymers with natural macromolecular polymers to develop gel electrolytes with superior performance, aiming to advance the development of AZIBs [137, 147, 320, 321, 322]. Hu and colleagues have constructed a novel organic‐inorganic hybrid hydrogel (denoted as CTPHE) through chemical crosslinking of CMCHS, sulfonated porous aromatic frameworks with ‒NH_2_ groups and sulfonic acid groups (PAF‐222‐SO_3_H) and tannic acid (TA), and demonstrated its remarkable effectiveness as an electrolyte in protecting zinc anodes (Figure 11a,b) [320]. The CMCHS chains and PAF‐222‐SO_3_H with hierarchical pore structure jointly constructed plenty of high‐speed ionic channels for zinc ion transport, and the –COOH, sulfonic, and ‒NH_2_ groups on both collaboratively facilitated the desolvation of zinc ions while homogenizing their interfacial distribution (Figure 11c). Meanwhile, while the ternary components including CMCHS constructed a 3D gel network capable of further inducing uniform deposition of zinc ions, the abundant functional groups on their surfaces, such as ‒COOH, ‒OH, ‒NH_2_, and sulfonic acid groups, were interconnected to form a 3D H‐bond network that could prevent water molecules from corroding the surface of the zinc electrode, thereby effectively suppressing zinc dendrites and interfacial side reactions (Figure 11d–g). Ran et al. incorporated graphene oxide (GO) into a CMC‐based gel system and fabricated a composite cellulose‐based hydrogel electrolyte with antiswelling properties and excellent ionic conductivity through chemical crosslinking to improve the electrochemical performance of zinc anodes [321]. The crosslinked cellulose chain network and GO sheets effectively channeled the rapid transport of zinc ions and homogenize the electric field at the electrode interface, thereby promoting uniform zinc ion deposition to yield a dendrite‐free zinc anode.

**FIGURE 11:**
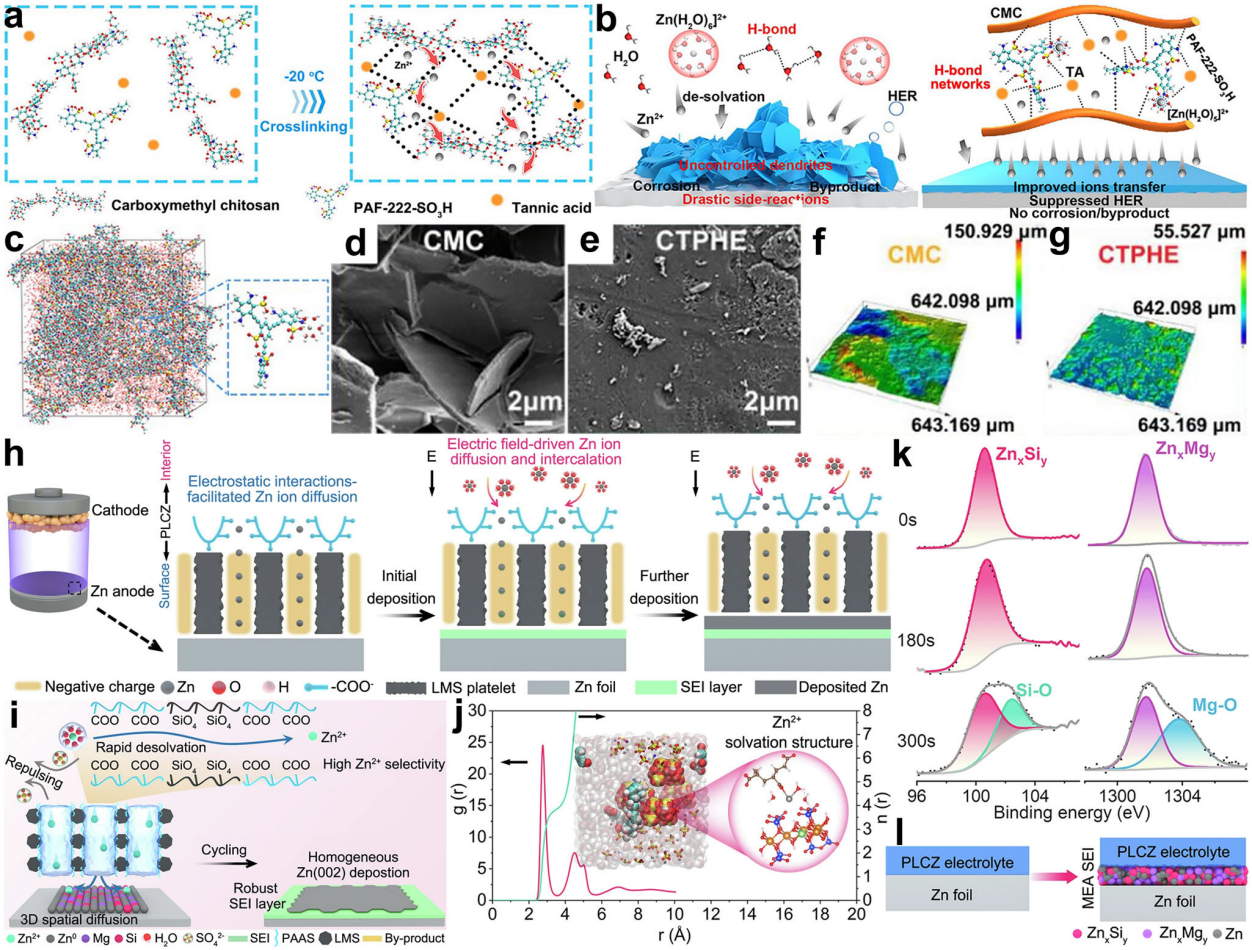
(a) Schematic diagram of the synthesis of CTPHE electrolyte. (b) Mechanism diagram of the effect of CTPHE electrolyte with and without PAF‐222‐SO_3_H on zinc anodes. (c) MD simulation of CTPHE electrolyte and enlarged solvation structure of zinc ions. (d, e) SEM characterizations of the cycled zinc anodes in different electrolytes. (f, g) CLSM images of the cycled zinc anodes in different electrolytes. Reproduced with permission [320]. Copyright 2025, Wiley‐VCH. (h) Schematic diagram of PLCZ electrolyte regulating the diffusion and transmission of zinc ions. (i) Schematic diagram of PLCZ electrolyte optimizing the deposition behavior of zinc ions on zinc anodes. (j) RDF simulation of Zn^2+^‐O and the solvation structure of zinc ions in PLCZ electrolyte. (k) Si 2p and Mg 1s x‐ray photoelectron spectroscopy (XPS) spectrums of zinc anodes after cycling with PLCZ electrolyte. (l) Schematic diagram of the generation of the medium‐entropy alloy SEI lay on the zinc electrode using PLCZ electrolyte. Reproduced with permission [137]. Copyright 2025, Springer Nature.

Moreover, lithium magnesium silicate (LMS) has also been employed to crosslink with sodium polyacrylate (PAAS) and CNFs, subsequently undergoing ion exchange with zinc ions to construct organo‐inorganic hybrid polymer‐based gel (PLCZ gel) electrolytes that were more conducive to enhancing the stability of zinc metal anodes [137]. The silicate tetrahedra within the LMS crystals structured numerous ionic channels through electrostatic bonding with carboxylate groups in the polymer chains. Zinc ions selectively accumulated within this constructed ion channels to generate an ion concentration gradient, and the combined effect of the concentration gradient and the electric field drove the rapid transport of zinc ions through these channels (Figure 11h). Simultaneously, these ion channels not only significantly regulated the solvation structure of zinc ions and facilitated their desolvation but also repelled SO_4_^2‒^ away from zinc anodes, effectively suppressing interfacial side reactions (Figure 11i,j). More intriguingly, LMS interacted with zinc ions to form a robust Zn‐Mg‐Si alloy SEI protective layer in situ on the electrode surface, effectively suppressing zinc dendrite growth (Figure 11k,l).

In comparison with inorganic functional materials, water‐soluble polymers such as polyacrylamide (PAM) have been more widely utilized in the combined construction of natural macromolecular polymer‐based gel electrolytes. In 2023, a kind of composite cellulose‐based gel electrolyte containing Zn^2+^ that significantly improved the cycling stability of zinc metal anodes was fabricated by crosslinking and polymerizing amphoteric CEQC with acrylamide monomers (PCZ‐gel electrolyte) [322]. The ampholytic cellulose chains, grafted with positively charged quaternary ammonium groups and negatively charged carboxyethyl groups, utilized these charged functional groups to construct multitudinous anion/cation migration pathways, facilitating the desolvation and rapid transport of zinc ions while ensuring their uniform distribution at the interface, which inhibited side reactions and promoted even zinc deposition. Concurrently, the crosslinked network formed by cellulose chains and PAM endowed this gel electrolyte with outstanding flexibility and mechanical strength, significantly inhibiting dendrite growth on zinc anodes.

Also, sulfonated chitosan could also be crosslinked with PAM to construct composite chitosan‐based gel electrolytes. Fan and collaborators discovered that the constructed sulfonated chitosan‐PAM hydrogel exhibited favorable mechanical properties and high ionic conductivity, significantly improving the electrochemical performance of zinc anodes when employed as an electrolyte for AZIBs [269]. Notably, owing to the promotion of desolvation of hydrated zinc ions and the entrapment of free water molecules by the sulfonate groups on the sulfonated chitosan molecular chains, this composite chitosan‐based hydrogel electrolyte enabled the uniform deposition of zinc ions on zinc anodes while suppressing side reactions and electrode corrosion. Another study also leveraged the properties of sulfonate groups by chemically crosslinking zwitterionic sulfobetaine (MDS) containing sulfonate groups with carboxylated chitosan and PAM to fabricate a composite polyzwitterionic chitosan‐based hydrogel electrolyte (SC‐PAM hydrogel electrolyte) with enhanced performance (Figure 12a–c) [147]. The zinc‐affinity sulfonate groups on MDS induced the formation of highly zinc ion‐selective transport pathways within SC‐PAM electrolyte, facilitating the rapid transport of zinc ions (Figure 12d,e). In the meanwhile, the –COOH groups on the carboxylated chitosan chains anchored water molecules and formed a hydrophobic interface layer on the zinc anode surface, inhibiting water‐induced electrode corrosion (Figure 12f). What's more, SC‐PAM facilitated uniform distribution of zinc ions at the electrode interface while equalizing the distribution of the interfacial electric field, inducing the uniform deposition of zinc ions and circumventing zinc dendrites (Figure 12g).

**FIGURE 12:**
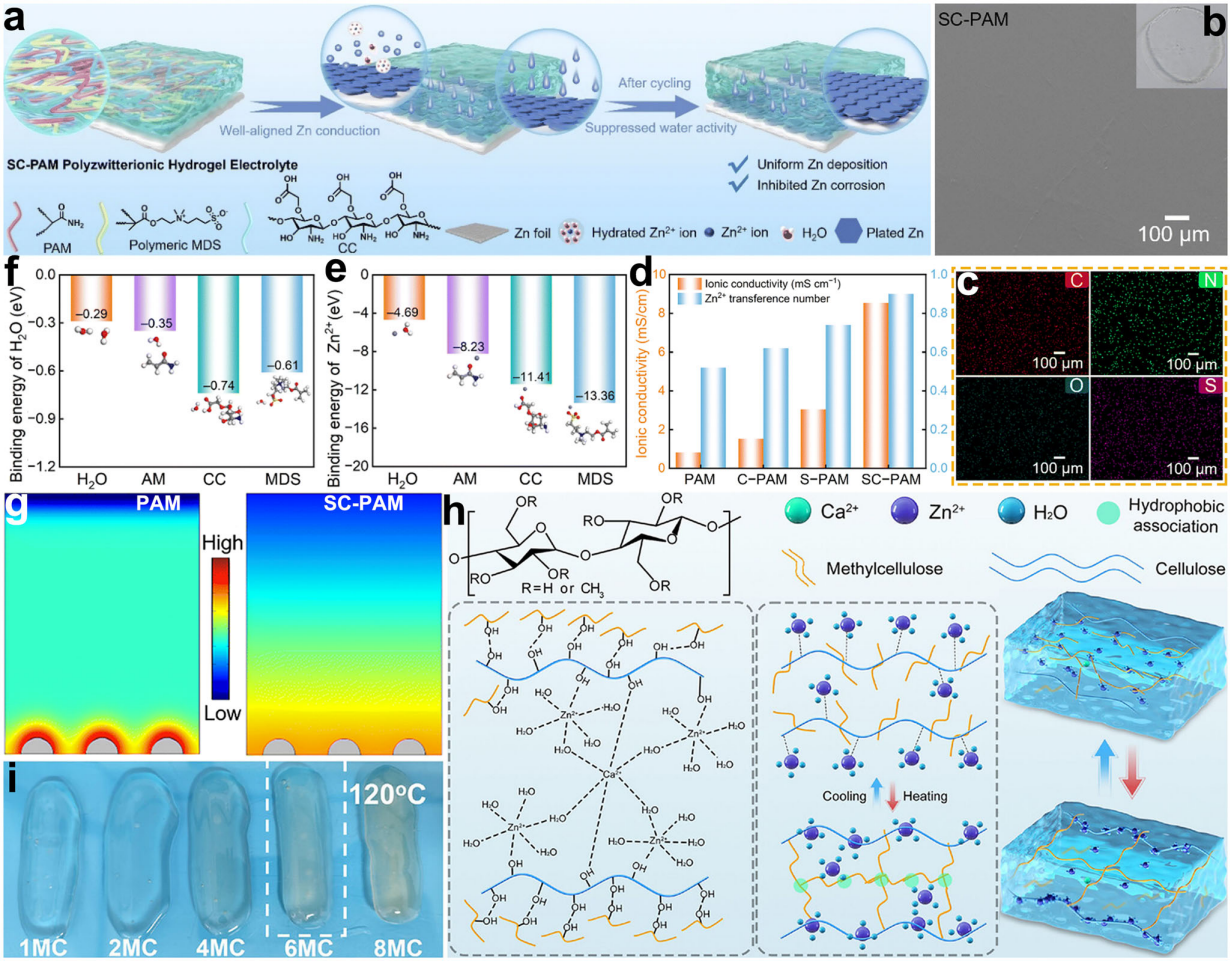
(a) Schematic depiction of SC‐PAM electrolyte regulating the deposition of zinc ions and suppressing interfacial corrosion. (b, c) SEM characterization and digital photograph (inset) of SC‐PAM electrolyte as well as corresponding energy dispersive spectroscopy (EDS) images. (d) The ionic conductivity and transfer number (zinc ions) in different electrolytes. (e, f) The binding energies of different components in SC‐PAM electrolyte with zinc ions and H_2_O respectively. (g) COMSOL simulations of the distribution of zinc ions on zinc anodes paired with PAM and SC‐PAM electrolytes. Reproduced with permission [147]. Copyright 2026, The Royal Society of Chemistry. (h) Mechanism diagram of constructing of high‐temperature‐resistant dual‐network cellulose‐based hydrogel. (i) Digital photograph of dual‐network cellulose‐based hydrogel containing different amounts of methylcellulose at 120°C. Reproduced with permission [249]. Copyright 2025, Wiley‐VCH.

More intriguingly, crosslinking Zn(ClO_4_)_2_ with PAM and chitosan yielded a composite Zn(ClO_4_)_2_‐PAM/chitosan (C‐PAMCS) hydrogel electrolyte exhibiting outstanding fatigue resistance [323]. Researchers found that the synthesized electrolyte can exhibit optimal fatigue resistance while ensuring uniform deposition of zinc ions. Researchers have found that the synthesized C‐PAMCS electrolyte could exhibit optimal fatigue resistance while ensuring uniform deposition of zinc ions, and have uncovered the mechanism by which the copolymer matrix and inorganic salt counteract crack initiation and propagation. The C‐PAMCS electrolyte delivered a significant improvement in the cycling stability of zinc anodes under high current densities and deposition capacities, enabling Zn||Zn symmetric batteries incorporating C‐PAMCS electrolyte to cycle stably for over 1500 h at 10 mA cm^‒2^/10 mAh cm^‒2^. Additionally, a portion of the research teams crosslinked trehalose with PAM to optimize the physical and chemical properties of PAM hydrogels, such as mechanical strength and interfacial chemistry characteristics, thereby constructing novel composite gel electrolytes with superior performance [253, 324]. Similarly, some researchers have crosslinked CNC as a building block into PAM‐chitosan hydrogels to further manufacture modified gel electrolytes with better mechanical properties and chemical adsorption/coordination capabilities [325]. All these optimized composite polymer electrolytes have demonstrated significant efficacy in enhancing the stability of zinc anodes, holding great significance for advancing the development of gel electrolytes for AZIBs.

In recent years, double polymer network gel electrolytes constructed by crosslinking two natural macromolecular polymers have been increasingly widely applied in AZIBs. This stems from the fact that such gel electrolytes integrating the advantages of both natural polymer components, typically exhibit greater potential for optimizing the cycling durability of zinc anodes, thereby stimulating considerable research interest among scholars. In order to overcome issues such as the difficulty in controlling the water content and the poor ionic conductivity of hydrogel electrolytes formed by crosslinking pure cellulose, Hu and cooperators combined nanocellulose and CMC to fabricate a composite hydrogel electrolyte featuring a dual‐cellulose network with high ionic conductivity and mechanical strength [326]. These CMC‐cellulose gel electrolytes possessed a moderate water content that ensured sufficient ionic conductivity for rapid zinc ion transport while preventing excessive water from causing surface corrosion of zinc anodes. Meanwhile, CMC, possessing both zinc‐affinity and hydrophilic properties, not only facilitated the transport of zinc ions but also effectively reduced free water at the electrode interface, thereby suppressing various water‐induced side reactions and promoting the uniform deposition of zinc ions. Furthermore, owing to their high stability and excellent mechanical properties, these composite hydrogel electrolytes effectively inhibited the growth of zinc dendrites over extended periods, significantly prolonging the cycling life of zinc anodes.

Recently, a study has utilized methylcellulose and cotton short fiber cellulose to jointly construct a cellulose‐based double‐network composite hydrogel electrolyte incorporating a high concentration of zinc salts (Figure 12h) [249]. These cotton linter celluloses simultaneously stabilized methylcellulose and high concentrations of zinc ions through their abundant –OH groups, preventing the precipitation of high‐concentration zinc salts at elevated temperatures due to incompatibility with methylcellulose, thereby allowing the composite cellulose‐based gel electrolyte to exhibit excellent high‐temperature stability (Figure 12i). Coupled with outstanding mechanical properties, both factors ensured that the synthesized gel electrolyte effectively enhanced the cycling stability of zinc anodes at elevated temperatures. Srinivasan et al. incorporated cellulose nanofibers (CNF) into chitosan‐based gels to construct a CNF‐chitosan polymer gel electrolyte with excellent mechanical properties, significantly enhancing the cycling stability of zinc anodes and enabling pouch batteries using them to stably cycle over 1000 cycles (Figure 13a,b) [22]. Thereinto, the chitosan polymer chains, rich in ‒NH_2_ and –OH groups, modulated the solvation structure of zinc ions and accelerated their transport, while synergizing with the chitosan polymer glue to promote uniform distribution of zinc ions at the electrode interface, resulting in uniform deposition of zinc ions without dendrites (Figure 13c,d). Furthermore, the CNF‐chitosan polymer gel electrolyte effectively anchored active water, thereby avoiding electrode corrosion and interfacial side reactions. Additionally, a kind of composite gel composed of LS and chitosan (LS‐chitosan gel) was developed by Sipponen and collaborators and utilized as an electrolyte to improve the electrochemical performance of AZIBs [327]. These dual natural polymer‐based gel electrolytes optimized the diffusion and transport of zinc ions, and their various functional groups, such as sulfonic groups, regulated the solvation structure of hydrated zinc ions and trapped free water molecules, thereby facilitating uniform deposition of zinc ions on zinc anodes and circumventing side reactions and zinc dendrites.

**FIGURE 13:**
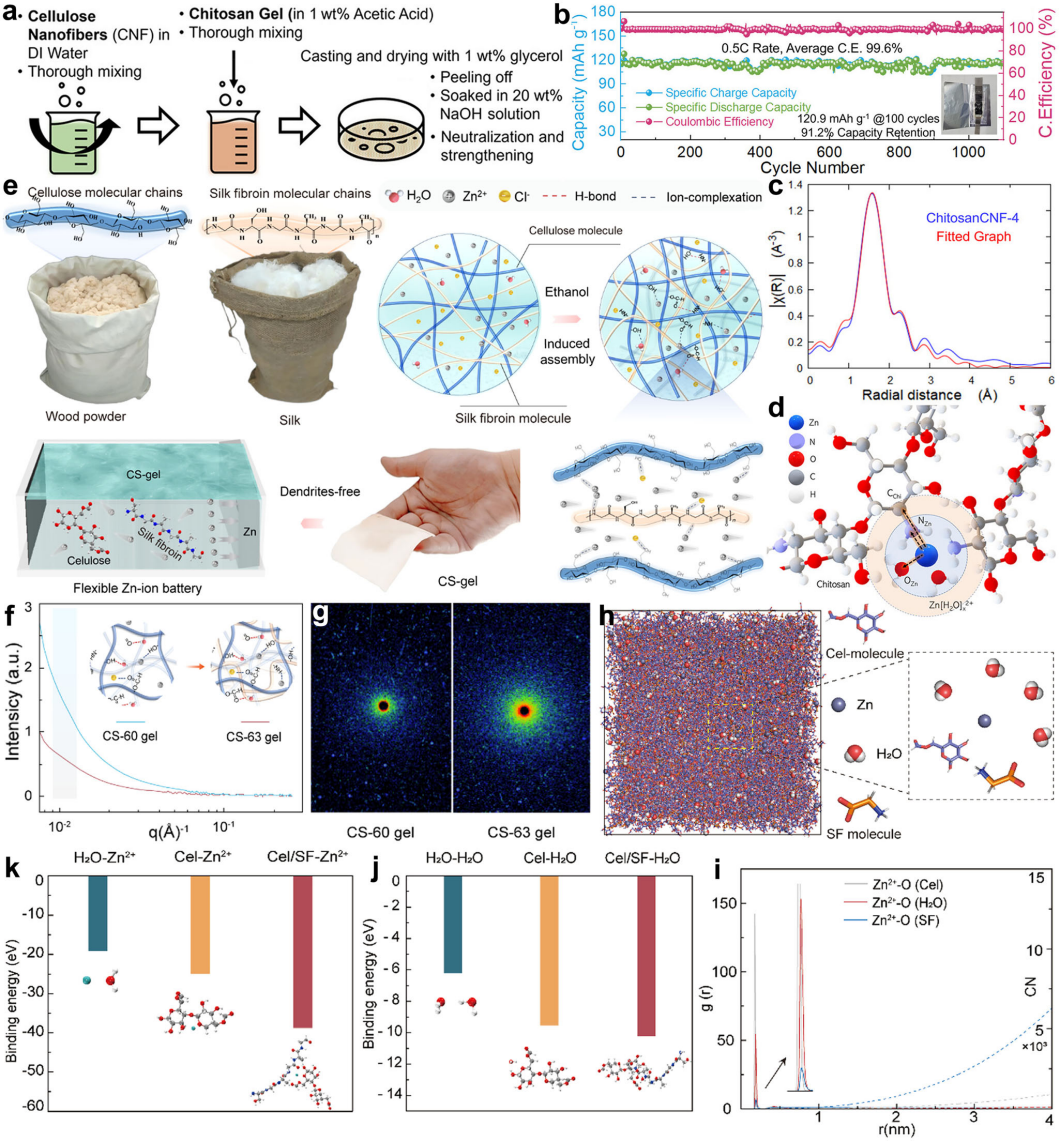
(a) Schematic diagram of the fabrication of CNF‐chitosan polymer gel. (b) The long‐term stability of the assembled Zn||LiMn_2_O_4_‐Carbon Fiber full pouch battery with CNF‐chitosan electrolyte. (c) Fourier transform extended X‐ray absorption fine structure (EXAFS) spectrums for CNF‐chitosan electrolyte. (d) Schematic illustration of the solvation structure of zinc ions in CNF‐chitosan electrolyte. Reproduced with permission [22]. Copyright 2025, Wiley‐VCH. (e) Schematic diagram of the ingredients, construction mechanism and action mechanism of CS‐gel. (f, g) SAXS profiles and corresponding 2D SAXS patterns of different CS‐gel‐based electrolytes. (h) MD simulation snapshot of CS‐gel and corresponding solvation structure of zinc ions. i) RDF of Zn^2+^ in CS‐gel. (j, k) The binding energies of H_2_O and zinc ions with different substances respectively. Reproduced with permission [98]. Copyright 2025, Elsevier.

On the other hand, amino acid polymers were also used in combination with natural polymer substrates commonly employed for gel construction, such as cellulose, chitosan, and alginate to jointly fabricate novel composite gel electrolytes. Yu and colleagues successfully constructed a new type of double‐network cellulose–silk fibroin gel (CS‐gel) by co‐dissolving silk fibroin and cellulose, followed by crosslinking and assembly, and demonstrated its outstanding effectiveness as an electrolyte in optimizing AZIBs (Figure 13e) [98]. This type of synthetic gel electrolyte featured the highly interpenetrating hydrogen‐bond networks and contained ordered *β*‐lamellar domains, as confirmed by multiple characterization techniques including SEM testing and small‐angle x‐ray scattering (SAXS) analysis (Figure 13f,g). It is precisely these structural advantages that endow these CS‐gel electrolytes with excellent mechanical properties and abundant ion channels, enabling them to withstand mechanical strain and facilitate the rapid transmission of zinc ions. Concurrently, these CS‐gel electrolytes were rich in polar functional groups such as –NH_2_ and –COOH, which effectively regulated the solvation structure of zinc ions and promoted the desolvation of hydrated zinc ions (Figure 13h). Furthermore, the strong hydrophilic and zinc‐binding properties enabled the CS‐gel electrolyte to accelerate the transport of zinc ions while restricting free active water, thereby promoting the uniform deposition of zinc ions and effectively suppressing side reactions as well as zinc dendrites (Figure 13i–k).

Xu and collaborators utilized polyaspartic acid (PASP) and chitosan, both abundant in the shells of mollusks such as snails, to jointly manufacture a biodegradable PASP‐chitosan hydrogel electrolyte, which was used as an electrolyte to significantly improve the stability of zinc metal anodes [328]. Due to the distribution of numerous anionic functional groups such as –O– and –COOH, PASP, and chitosan chains exhibited high zinc affinity, effectively optimizing the transport and interfacial distribution uniformity of zinc ions, enabling the constructed PASP‐chitosan hydrogel electrolyte to induce the uniform deposition of zinc ions along the Zn (002) plane. Moreover, the PASP‐chitosan hydrogel electrolyte not only possessed excellent mechanical properties, but the abundant anions within it also restricted the decomposition of free water molecules, thereby significantly inhibiting side reactions and eliminating zinc dendrites. Besides, Hu et al. developed a dual‐network composite hydrogel electrolyte featuring abundant carboxylate ions (Gel/SA‐acetate electrolyte) constructed through crosslinking of gelatin, SA, and acetate, dedicated to stabilizing zinc metal anodes [329]. The zinc‐philic functional groups on the polymer chains interacted with water molecules to reconfigure the H‐bond network, while regulating the solvation structure of hydrated zinc ions. The optimized hydrated zinc ions underwent further desolvation as they were directionally transported along the polymer chains, achieving uniform distribution at the electrode interface and subsequent uniform deposition on zinc anodes. Meanwhile, the constructed Gel/SA‐acetate electrolyte strongly bound and interacted with zinc anodes, reducing the activity of interface water and minimizing contact between water molecules and zinc electrodes, thereby significantly suppressing side reactions such as HER and zinc dendrites. As a result, the assembled Zn||Zn symmetric battery based on these Gel/SA‐acetate electrolytes exhibited excellent cycling stability, maintaining operation for 1580 h at 0.1 mA cm^‒2^/0.1 mAh cm^‒2^.

Apart from the widely employed chemical/physical crosslinking strategies, some promising techniques such as electro‐assisted crosslinking, electrogelation, and chemical grafting are increasingly being explored for constructing gel electrolytes suitable for AZIBs [155, 319, 330, 331, 332]. Zhou and colleagues developed a green electro‐crosslinking strategy to successfully fabricate a zinc alginate gel (Zn‐Alg) electrolyte for building wire‐shaped AZIBs [330]. This electro‐crosslinking approach relied on electrochemical oxidation to activate free zinc ions and the –COOH groups of alginates for superionic binding, enabling rapid gel formation without the use of toxic chemical initiators or reagents. This Zn‐Alg electrolyte was in situ generated on the surface of zinc anodes, featuring high ionic conductivity, which promoted the uniform deposition of zinc ions and suppressed zinc dendrites as well as side reactions, significantly improving the cycling stability of zinc anodes. Meanwhile, an electrogelation technique capable of controllable construction of natural macromolecular polymer‐based hydrogels was proposed by Chen and collaborators and was successfully employed to in situ structure chitosan‐based hydrogel electrolyte with high ionic conductivity and excellent mechanical properties on zinc anodes [319]. These chitosan‐based hydrogel electrolytes effectively restrained free water, thereby significantly inhibiting side reactions such as HER and electrode corrosion and the growth of zinc dendrites, enabling zinc anodes to maintain excellent cycling stability over a wide temperature range from −15°C to 110°C. Such wide‐temperature performance is closely related to the segmental dynamics of natural polymer chains and the stability of the H‐bond network. At low temperatures, the polar groups on the polymer chains form H‐bonds with water molecules, disrupting the orderly arrangement of water molecules, inhibiting the formation of ice crystals, and maintaining the ion transport channels. At high temperatures, the stable crosslinked H‐bond network helps retain water, maintain structural integrity, and reduce electrolyte volatilization, thereby enabling stable ionic conduction and interfacial compatibility over a broad temperature range. As the temperature changes, differences in the kinetic properties of relatively independent segments along the polymer chains lead to differentiation in the kinetic behavior of water molecules, thereby inhibiting ice formation at low temperatures and water loss at high temperatures, and consequently making a significant contribution to the wide‐temperature performance of such hydrogel electrolytes.

Wang et al. validated the feasibility of an electrophoresis‐driven phase separation technique for in situ fabrication of a mesoporous chitosan‐based gel quasi‐solid electrolyte on zinc anodes [155]. The newly constructed electrolytes were composed of twisted chitosan nanofiber bundles with ‒NH_2_ groups, exhibiting a 3D framework with continuous spindle‐shaped pores and positively charged surfaces. The aforementioned structural and surface characteristics endowed these electrolytes with high mechanical strength and ionic conductivity, promoting the uniform deposition of zinc ions while inhibiting zinc dendrites and alleviating harmful side reactions such as electrode corrosion, thereby achieving the long‐term cycling stability of zinc anodes. Another research constructed a novel SA/electron‐ionic dual transport channel (SA/EIDC) gel electrolyte by coating a copolymer of poly (3,4‐ethylenedioxythiophene) and polystyrene sulfonate (PEDOT:PSS) onto SA gel and then performing interlayer chemical crosslinking (Figure 14a,b) [332]. These SA/EIDC gel electrolytes exhibited a porous structure and excellent mechanical properties, significantly optimizing the electrochemical performance of zinc anodes (Figure 14c). Thereinto, the SA gel layer effectively altered the solvation structure of hydrated zinc ions, accelerating their desolvation process (Figure 14d,e). At the same time, the PEDOT polymer chains provided ample electron migration pathways for speedy electron transfer, while the linear PSS chains, through their polar groups interacting with zinc ions, offered numerous ionic transmit channels for the rapid transport of zinc ions (Figure 14f–h). The synergistic effect of the three components facilitated the uniform migration and deposition of zinc ions on zinc anodes, inhibiting zinc dendrites and various side reactions. Also, Zhou and cooperators cleverly grafted polyacrylic acid (PAA) onto the surface of cellulose hydrogels and then soaked them in a zinc sulfate solution to fabricate a kind of carboxylated zinc‐ion‐containing composite Cel@PAA (CPZ) gel electrolyte, which effectively improved the stability of zinc anodes [331]. Specifically, the cellulose layer in these CPZ gel electrolytes regulated and guided the uniform transport and deposition of zinc ions, while the PAA layer offered ample protons to the cathode. Consequently, the synergistic action of the cellulose gel layer and PAA layer enabled the CPZ gel electrolyte to suppress dendrite growth and side reactions on zinc anodes.

**FIGURE 14:**
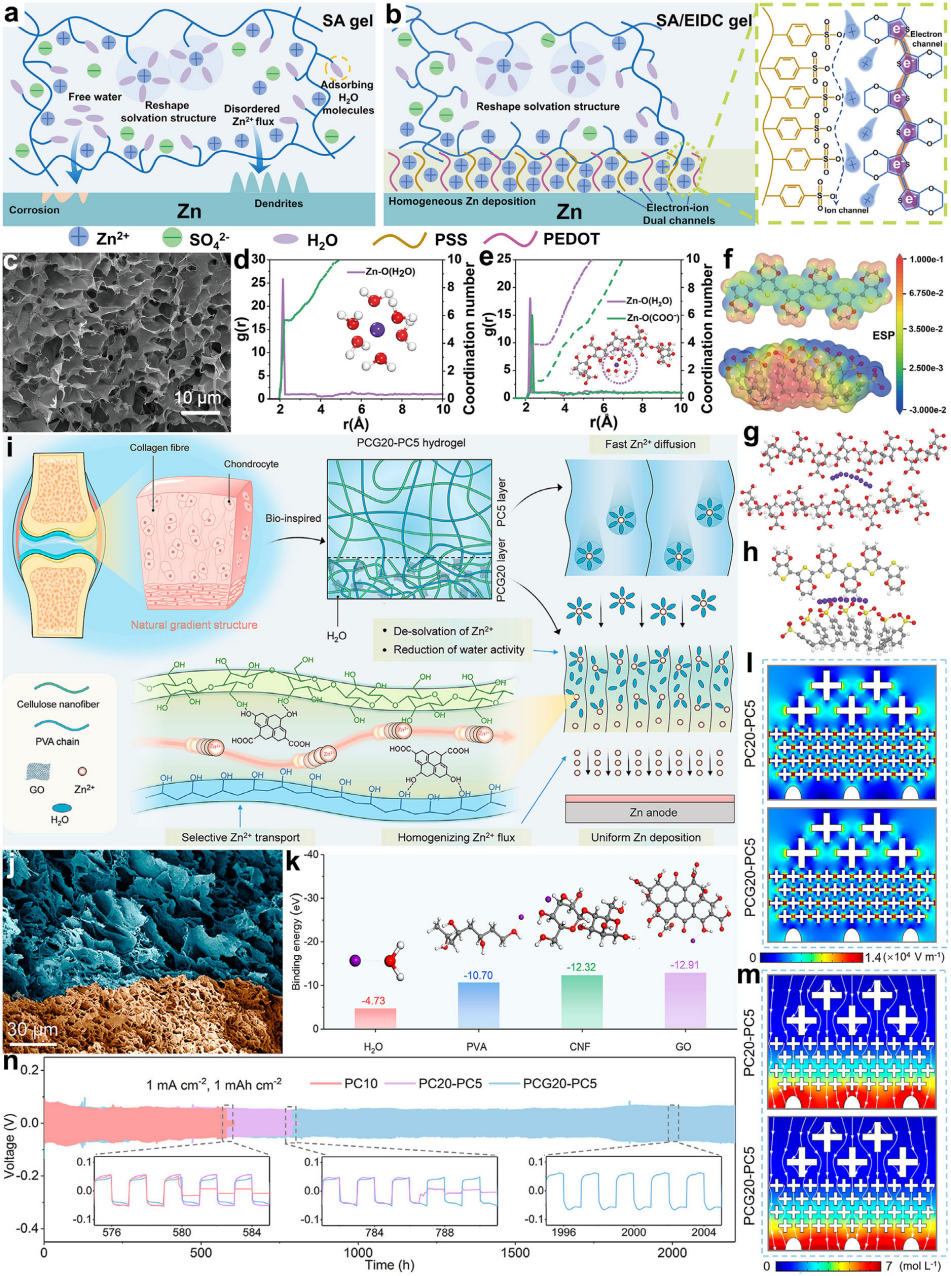
(a, b) Schematic diagram of the regulation of deposition of zinc ions by SA gel and SA/EIDC gel electrolytes. (c) SEM characterization of SA/EIDC gel after freeze‐drying. (d, e) RDFs of Zn^2+^‐H_2_O in LE and SA gel electrolyte as well as corresponding solvation structures of zinc ions. (f) ESP simulations of PEDOT and PSS chains. (g, h) The transport paths of zinc ions along the SA molecular chains and the PSS/PEDOT chains. Reproduced with permission [332]. Copyright 2025, Elsevier. (i) Schematic depiction illustrating the regulation of the transport and deposition behaviors of zinc ions by PCG20–PC5 electrolyte inspired by cartilage. (j) SEM characterization of PCG20–PC5 hydrogel. (k) The binding energies of each component in PCG20–PC5 hydrogel with zinc ions. (l) Electric field and (m) ion distribution simulations on zinc anodes with different electrolytes. (n) The long‐cycle performance of Zn||Zn symmetric batteries using different electrolytes. Reproduced with permission [334]. Copyright 2025, American Chemical Society.

Furthermore, a few research teams have developed some natural macromolecular polymer gel electrolytes with special structures through ingenious design and methodological innovations [26, 109, 333, 334]. To mimic wound healing, Li and colleagues constructed a novel chitosan‐based gel/solid hybrid electrolyte (gel/solid‐chitosan electrolyte) through a polymerization‐protonation strategy to achieve the re‐repair of the damaged zinc anodes [333]. It should be noted that the zinc‐chitosan solid electrolyte that constituted these gel/solid hybrid electrolytes was located between zinc anodes and the protonated chitosan gel electrolyte and closely adhered to zinc anodes, forming a layer‐by‐layer distributed structure. These gel/solid electrolytes effectively optimized the transport and interface distribution uniformity of zinc ions while reducing the activity of free water, significantly inhibiting zinc dendrites and side reactions such as HER, thereby markedly enhancing the cycling stability of zinc anodes. Meanwhile, proton neutralization and zinc ion complexation enabled these gel/solid electrolytes to eliminate corrosion byproducts and smooth electrode surface defects, further prolonging the cycling life of zinc anodes. Also, Chen et al. drew inspiration from the composition of articular cartilage and designed and fabricated a gradient network hydrogel electrolyte composed of PVA, CNF, and GO (PCG20–PC5), aiming to optimize AZIBs (Figure 14i) [334]. These constructed PCG20–PC5 electrolytes featured the high‐density PVA/CNF/GO (PCG) networks on the side close to zinc anode, while the side farther from zinc anode consisted of a PVA/CNF (PC) hydrogel layer with a lower network density (Figure 14j). Interestingly, the PC hydrogel layer, rich in ion channels and abundant water, facilitated the rapid transmission of zinc ions, while the PCG hydrogel layer contained numerous zinc‐affinitive polar groups such as –COOH and –OH, which accelerated the desolvation of hydrated zinc ions and enabled the uniform distribution of the electric field and zinc ions at the electrode interface, and simultaneously weakening the activity of interfacial water (Figure 14k–m). Thanks to the synergistic effect of each part of these gradient network hydrogel electrolytes, the PCG20–PC5 electrolytes effectively suppressed side reactions and zinc dendrites while ensuring rapid reaction kinetics, enabling the optimized zinc anode to exhibit excellent cycling durability (Figure 14n).

Another study successfully constructed a novel ultrathin cellulose‐based gel with a gradient hydrophobic interface (DCG) by dissolving cellulose in a deep eutectic solvent (DES) followed by regeneration, which effectively improved the stability of zinc anodes when used as the electrolyte in AZIBs [26]. These DCG electrolytes featured high mechanical strength and abundant ion transmission channels, providing a guarantee for the stability of battery structure and the rapid transport of zinc ions. Notably, the gradient hydrophobic interface formed by trace DES on the side of the DCG electrolyte near zinc anodes excluding water molecules at the electrode interface, effectively restraining water‐induced side reactions and guiding zinc ions to deposit uniformly along the Zn (002) facet, thereby eliminating zinc dendrites. In addition, Zhang and collaborators fabricated a kind of sol‐gel gradient electrolyte with a novel configuration through the electrochemical co‐deposition of zinc ions and alginate [109]. This constructed sol‐gel gradient electrolyte integrated the advantages of both aqueous electrolytes and gel electrolytes while compensating for each of their respective shortcomings, effectively improving the cycling reversibility of zinc anodes. To be specific, zinc ions in the sol region farther away from zinc anodes followed the same vehicular mechanism‐driven transport as in aqueous electrolytes, and were transmitted at high speed. Meanwhile, the gel layer formed by alginate gelation in the sol‐gel gradient electrolyte closely adhered to zinc anodes, lowering the activity of interfacial water and confining the 2D deposition/stripping of zinc ions within the alginate ‘egg‐shell’ structures, thereby effectively suppressing side reactions and the generation of zinc dendrites.

Reviewing the selection of raw materials for the aforementioned natural macromolecular polymer‐based gel electrolytes, their cost advantages are evident. Regarding the assessment of the environmental friendliness and sustainability of such electrolytes, some researchers have analyzed the biodegradability and recyclability of the constructed natural polymer‐based gel electrolytes. For instance, Xu et al. demonstrated that the constructed PASP‐chitosan hydrogel electrolytes could be recovered from post‐cycled Zn/MnO_2_ batteries for subsequent reuse and they also monitored the complete degradation process of PASP‐chitosan hydrogel electrolytes in soil, fully confirming that this inexpensive and readily fabricated hydrogel electrolyte had excellent recyclability and biodegradability [328]. Hu and collaborators not only confirmed the complete biodegradation of their synthesized chitosan‐based gel electrolytes in soil but also demonstrated their non‐flammable properties, fully verifying both the biodegradability and high safety of these chitosan‐based gel electrolytes [159]. Furthermore, the chitosan‐based hydrogel electrolyte developed by Chen et al. had also been demonstrated to be recoverable and reusable through a straightforward processing procedure, and their economic advantages and environmental friendliness had also been validated through techno‐economic analyses and life cycle assessment [319]. Concurrently, benefitting from the structural advantages and surface properties of natural macromolecular polymers, some of the constructed natural macromolecular polymer‐based gel electrolytes exhibited numerous benefits including flexibility, self‐healing capabilities, freeze resistance, and high‐temperature tolerance. It is precisely these multifaceted advantages that have enabled the progressive expansion of natural macromolecular polymer‐based gel electrolytes into applications such as wearable flexible batteries and energy storage in extreme environments, showing an accelerating development trend, which further reflected the enormous application potential of such electrolytes [26, 134, 319, 335].

All in all, the development and utilization of natural macromolecular polymer gel electrolytes have made a significant contribution to optimizing the overall electrochemical performance of AZIBs by improving the stability of zinc anodes. These sorts of gel electrolytes constructed so far each have their own characteristics, and their key performance metrics, such as ionic conductivity, were detailed in Table 2 for peers to further understand and compare the features and superiorities of these gel electrolytes.

**TABLE 2:** Ionic conductivities and mechanical properties of natural macromolecular polymer‐based gel electrolytes and their cycling stabilities in Zn||Zn symmetric batteries.

Gel electrolyte	Ionic conductivity	Mechanical property	Cycling performance of symmetric Zn||Zn cells	Refs
Dual cross‐linked cellulose gel	38.6 mS cm^−1^	Tensile strength of 4.46 MPa	2000 h (0.5 mA cm^−2^/0.5 mAh cm^−2^)	[107]
DCH‐S	24.1 mS cm^−1^	Tensile strength of 0.53 MPa	1850 h (1 mA cm^−2^/1 mAh cm^−2^)	[118]
COOH‐f‐CellPZ gel	10.6 mS cm^−1^	Tensile strength of 220 kPa	5250 h (0.5 mA cm^−2^/0.5 mAh cm^−2^)	[318]
Chitosan‐Zn	71.8 mS cm^−1^	Tensile strength of 7.4 MPa	1000 h (50 mA cm^−2^/10 mAh cm^−2^)	[159]
SA‐Zn(Ac)_2_ hydrogel	32.8 mS cm^−1^	—	600 h (2 mA cm^−2^/2 mAh cm^−2^)	[315]
Zinc‐alginate /Nafion gel	25.5 mS cm^−1^	Tensile strength of 345 kPa	4600 h (1 mA cm^−2^/ 1 mAh cm^−2^)	[134]
CTPHE	2.68 S m^−1^	Tensile strength of 2.3 MPa	4000 h (1 mA cm^−2^/1 mAh cm^−2^)	[320]
CMC/GO	20.5 mS cm^−1^	Tensile strength of 1.38 kPa	800 h (1 mA cm^−2^/1 mAh cm^−2^)	[321]
PLCZ gel	10‐40 mS cm^−1^ (from 263.15 to 353.15 K)	—	4400 h (0.5 mA cm^−2^/0.25 mAh cm^−2^)	[137]
PCZ‐gel	27.5 mS cm^−1^	Tensile strength of 200 kPa	3000 h (0.5 mA cm^−2^/ 0.5 mAh cm^−2^)	[322]
Sulfonated chitosan‐PAM hydrogel	38.1 mS cm^−1^	Tensile strength of 54.2 kPa	2600 h (1 mA cm^−2^/1 mAh cm^−2^)	[269]
SC‐PAM	8.54 mS cm^−1^	—	7500 h (0.5 mA cm^−2^/ 0.5 mAh cm^−2^)	[147]
C‐PAMCS hydrogel	22.6 mS cm^−1^	Tensile strength of 50 kPa	1500 h (10 mA cm^−2^/10 mAh cm^−2^)	[323]
PAM/trehalose gel	1.93 S m^−1^	Tensile strength of 55 kPa	1200 h (1 mA cm^−2^/1 mAh cm^−2^)	[324]
(PAM)/ 45% trehalose	10.98 mS cm^−1^	Tensile strength of 100 kPa	2400 h (1 mA cm^−2^/1 mAh cm^−2^)	[253]
Hydroxypropyl Chitosan/PAM gel	36.2 mS cm^−1^	Tensile strength of 180 kPa	1800 h (1 mA cm^−2^/1 mAh cm^−2^)	[325]
CMC‐Cellulose gel	11 mS cm^−1^	Tensile strength of 70 ± 5 MPa	3500 cycles (80 mA cm^−2^/4 mAh cm^−2^)	[326]
Cellulose/6 methylcellulose gel	17.7‐26.3 mS cm^−1^ (from 40°C to 120°C)	Tensile strength of 126 kPa	—	[249]
CNF‐chitosan polymer gel	—	Tensile strength of 30.1 ± 3.9 MPa	2750 h (1 mA cm^−2^/1 mAh cm^−2^)	[22]
LS–Chitosan gel	18.6 mS cm^−1^	Tensile strength of 940 ± 63 MPa	—	[327]
CS gel	14.39 mS cm^−1^	Tensile strength of 1.14 MPa	1500 h (0.5 mA cm^−2^/0.5 mAh cm^−2^)	[98]
PASP‐chitosan gel	5.51 mS cm^−1^	Tensile strength of 53.2 kPa	900 h (25 mA cm^−2^/25 mAh cm^−2^)	[328]
Gel/SA‐acetate	5.1 mS cm^−1^	Tensile strength of 300.1 kPa	1580 h (0.1 mA cm^−2^/0.1 mAh cm^−2^)	[329]
Zn‐Alg gel	2.5 mS cm^−1^	—	500 h (5 mA cm^−2^/1.25 mAh cm^−2^)	[330]
Electrogelated chitosan gel	19.5 mS cm^−1^	Tensile strength of 4.4 MPa	2800 h (1 mA cm^−2^/1 mAh cm^−2^)	[319]
Mesoporous chitosan gel	25.1 mS cm^−1^	—	2500 h (1 mA cm^−2^/1 mAh cm^−2^)	[155]
SA/EIDC gel	41 mS cm^−1^	Tensile strength of about 480 kPa	6750 h (1 mA cm^−2^/1 mAh cm^−2^)	[332]
CPZ gel	16.4 mS cm^−1^	Tensile strength of 7.09 MPa	1000 h (50 mA cm^−2^/10 mAh cm^−2^)	[331]
Gel/solid‐chitosan	2.4 µm cm^−1^	—	1200 h (10 mA cm^−2^/1 mAh cm^−2^)	[333]
PCG20–PC5 hydrogel	16.18 mS cm^−1^	Tensile strength of 0.43 MPa	930 h (5 mA cm^−2^/1 mAh cm^−2^)	[334]
Ultrathin cellulose‐based gel	29.5 mS cm^−1^	Tensile strength of over 0.82 MPa	4000 h (4 mA cm^−2^/4 mAh cm^−2^)	[26]
Sol‐gel gradient alginate gel	about 40 mS cm^−1^	—	200 h (10 mA cm^−2^/10 mAh cm^−2^)	[109]

### 3.3 Functional Separators

In addition to constructing artificial interface SEI layers and optimizing electrolytes, developing functional separators based on natural macromolecular polymers also represents a significant and highly promising approach for enhancing the cycling stability of zinc anodes in AZIBs [140, 336]. Owing to the outstanding physical and chemical properties of natural polymers, they possess unique advantages in constructing functional separators that are conducive to improving the electrochemical performance of AZIBs. This significant research field has garnered increasing attention, with numerous studies emerging in recent years [120, 149, 337, 338, 339]. These existing researches can be subdivided into two mainstream research directions: functionalizing GF separators or filter paper by combining them with these polymers, and constructing natural polymer‐based self‐supporting separators [120, 121, 136, 338, 340, 341, 342, 343, 344].

Regarding the former research direction, it is readily understood that it typically involves using GF separators or filter papers as the framework, integrating the benefits brought by natural macromolecular polymers, such as high mechanical strength, multifunctional group adsorption, and efficient ion transport to develop advanced composite separators. Over the past few years, the endeavors of several research teams have yielded promising results [149, 340, 345]. Relatively early on, a research group utilized a spin‐coating technique to composite sulfonated cellulose‐improved G sheets onto the commercial GF membrane to fabricate a sort of functional Janus separator with entirely distinct compositions and properties on each side [340]. This engineered Janus separator exhibited non‐flammability, readily wettability, high ionic conductivity, and outstanding mechanical properties. The sulfonated cellulose chains with negative charges and abundant ‒OH repelled SO_4_^2‒^ while effectively anchoring protons, enabling these Janus separators to selectively permeate zinc ions, thereby suppressing side reactions such as interfacial corrosion and HER. Owing to the relatively small lattice mismatch with the Zn (002) facet, G sheets guided zinc ions to deposit horizontally along the Zn (002) crystal plane [340, 345], thus effectively restraining the growth of zinc dendrites. Consequently, the cycling stability of zinc anode was markedly enhanced under the multiple functions of the Janus composite separators.

In another study, Wang and collaborators fabricated a functional composite separator with a multi‐scale ion sieving effect by sequentially constructing a nanocellulose layer as an intermediate layer and a surface‐exposed PEDOT:PSS layer on a GF separator through stepwise vacuum filtration [136]. On one hand, the PEDOT:PSS layer, which was rich in sulfonic acid groups and possessed excellent ionic and electronic conductivity, not only confined the disordered migration of SO_4_^2‒^ but also homogenized the electric field distribution at the electrode interface. On the other hand, the CNF layer with uniform mesopores facilitated faster and more uniform transport of zinc ions. Due to the synergistic optimization effect of the PEDOT:PSS layer and the nanocellulose layer, these constructed functionalized composite separators effectively promoted the uniform deposition of zinc ions while inhibited the generation of byproducts, resulting in the dendrite‐free zinc anodes that circumvented severe side reactions. Besides, some researchers have also employed the flexible composite membrane composed of CNFs and aramid nanofibers (ANFs) as the interlayer in combination with a GF separator to optimize the interface stability of zinc anodes [149]. The constructed flexible composite membrane, rich in polar groups and featuring a uniform and dense fiber network structure, overcame the limitations of conventional GF separators, effectively promoting desolvation, transport, and uniform deposition of zinc ions, thereby effectively suppressing side reactions and zinc dendrites.

Beyond utilizing GF separators as the substrates to manufacture functional composite separators modified with natural polymers, other researches have also employed filter paper as the substrates for compositing these polymers. For instance, a chitosan‐coated filter paper separator, employed to optimize the electrochemical performance of zinc anodes, was readily constructed by thoroughly soaking traditional filter paper in a solution containing chitosan followed by rinsing [341]. The functional groups such as ‒NH_2_ and –O– groups on the chitosan molecular chains endowed the constructed chitosan‐coated filter paper separator with excellent ion adsorption capacity. Therefore, these functionalized filter paper separators not only accelerated the transport of zinc ions and restricted the contact between SO_4_^2‒^ and zinc anodes by effectively adsorbing zinc ions and SO_4_^2‒^, achieving the uniform deposition of zinc ions while suppressing the generation of byproducts, but also efficiently anchored protons to suppress their activity, thereby inhibiting side reactions such as HER on zinc anodes. In addition, Yang and collaborators recently constructed a composite layer composed of SA and halloysite nanotubes (HNTs) on cellulose filter paper through vacuum filtration, fabricating a new type of composite (SA/HNTs‐filter paper) separators that combined multiple functions, including ion sieving and ion transport kinetics regulation (Figure 15a,b) [343]. These HNTs, which have both positive and negative charges on their surfaces, respectively adsorbed zinc ions and SO_4_^2‒^ on their outer and inner surfaces, thereby restricting the irregular shuttle of SO_4_^2‒^ while promoting the efficient transport of zinc ions (Figure 15c). Meanwhile, the SA molecular chains rich in hydrophilic functional groups exhibited strong zinc affinity, promoting the desolvation of zinc ions and their uniform distribution on the electrode surface while reducing the interfacial water molecules (Figure 15d,e). The aforementioned favorable ion sieving and ion transport kinetics improvements enabled these SA/HNTs‐filter paper separators with good wettability and mechanical strength to promote the uniform deposition of zinc ions along the Zn (002) facet (Figure 15f), prevent zinc dendrites, and suppress various electrode corrosion side reactions, thereby significantly improving the cycling stability of zinc anodes.

**FIGURE 15:**
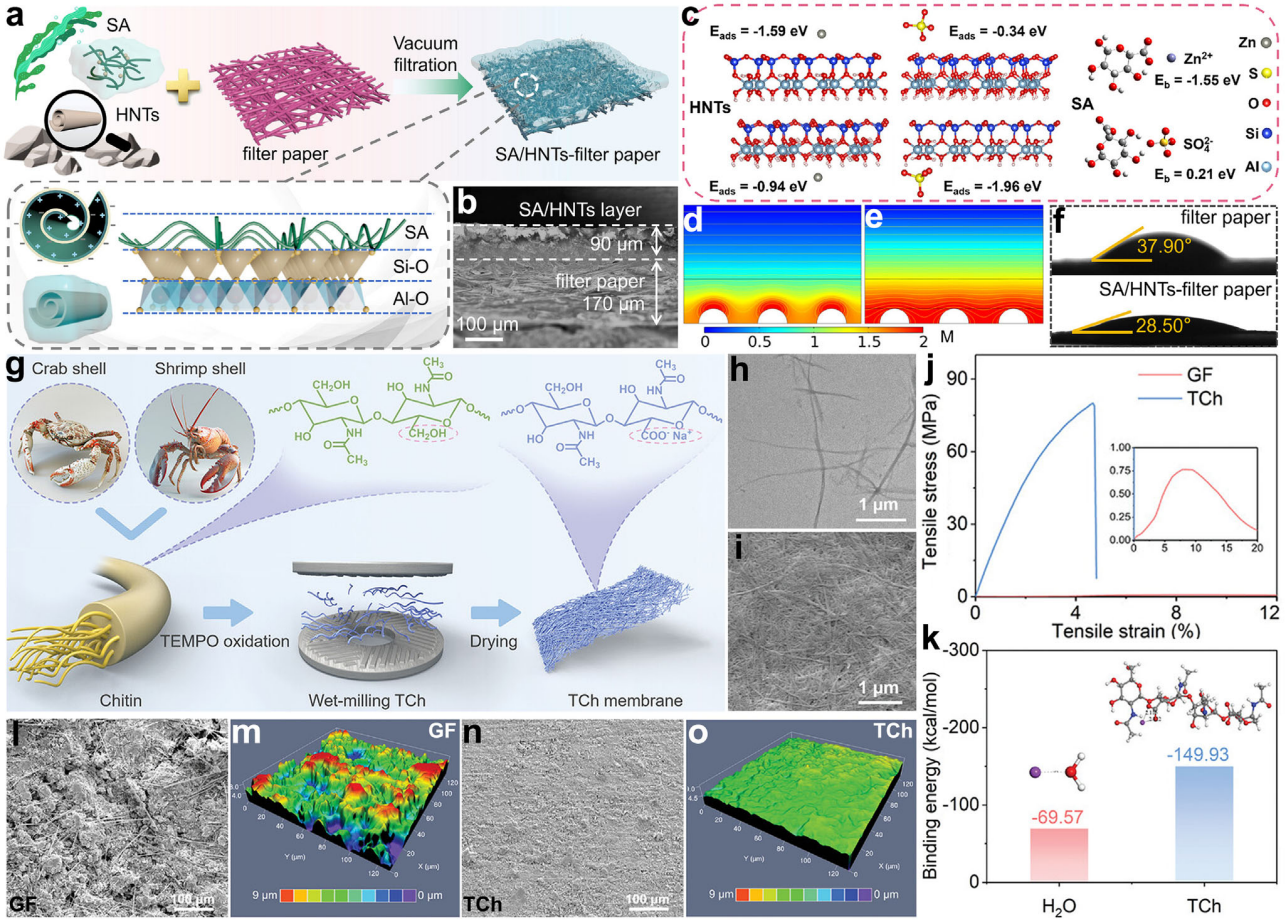
(a) Illustration of the construction of SA/HNTs‐filter paper. (b) SEM characterization of the cross‐section of SA/HNTs‐filter paper. (c) The adsorption configurations and corresponding adsorption energies of HNTs and SA for zinc ions and SO_4_^2‒^. (d, e) The simulated concentration field of zinc ions on zinc anodes with filter paper and SA/HNTs‐filter paper separators. (f) Wetting angles of different filter papers. Reproduced with permission [343]. Copyright 2025, Wiley‐VCH. (g) Schematic diagram of the construction of the TCh separator. (h) TEM characterization of the TCh nanofibers. (i) SEM characterization of the TCh separator. (j) Stress‐strain profiles of different separators. (k) The binding energies of zinc ions with H_2_O and TCh. (l–o) SEM and CLSM characterizations of the cycled zinc anodes protected by different separators. Reproduced with permission [120]. Copyright 2024, Wiley‐VCH.

Unlike the improved GF separators and filter papers functionalized with natural macromolecular polymers, natural macromolecular polymer‐based self‐supporting separators typically employed natural polymer chains and fibers as their structural framework [120, 337, 338]. The composition of these natural polymer‐based self‐supporting separators used in AZIBs included various forms such as single natural component, multiple natural components, and the integration of natural components with inorganic materials or organic polymers. Thereinto, the self‐supporting separators composed of single natural polymer components were the earliest to be investigated, generally exhibiting relatively simpler structures. In 2021, Chen and collaborators fabricated a self‐supporting cotton cellulose film through simple vacuum filtration and used it as a functionalized separator to replace the traditional GF separators to overcome the challenges faced by zinc anodes [337]. The constructed cellulose separators, rich in ‒OH and featuring uniform nanopores, exhibited high ionic conductivity and outstanding mechanical properties. By effectively reducing the desolvation energy barrier for hydrated zinc ions and the nucleation overpotential for the deposition of zinc ions, this cellulose separator significantly accelerated the transport and deposition kinetics of zinc ions, thereby markedly enhancing the cycling durability of zinc anodes.

In addition, a biodegradable self‐supporting membrane composed of lignin‐containing cellulose nanofibers (LCNFs) derived from waste palm has also been employed to optimize AZIBs [338]. These constructed LCNF membranes with a hydrophobic surface and uniform nanopores exhibited high ionic conductivity and wonderful mechanical properties. As the functionalized self‐supporting separators, they effectively reduced active water molecules at the electrode interface and accelerated the transport and deposition kinetics of zinc ions, thereby efficiently restraining corrosion side reactions and zinc dendrites, enabling zinc anodes to demonstrate outstanding cycling performance. As the research progressed, some chemically treated natural macromolecular polymers were also employed to construct functionalized self‐supporting separators. For instance, a self‐supporting membrane, constructed by casting, drying, and subsequent hot pressing of 2,2,6,6‐tetramethylpiperidin‐1‐oxyl (TEMPO) oxidized chitin (TCh) nanofibers derived from discarded shrimp and crab shells, was employed as a functionalized separator to enhance the electrochemical performance of zinc anodes (Figure 15g,h) [120]. These manufactured TCh separators not only possessed uniform nanopores and high mechanical strength, but also contained abundant zinc‐affinity polar groups, which facilitated the desolvation of hydrated zinc ions while enhancing the transport kinetics of zinc ions and their uniform distribution at the electrode interface, thereby inducing uniform deposition of zinc ions (Figure 15i–k). At the same time, electrochemical tests showed that these TCh separators also effectively reduced the nucleation barrier of zinc ions and corrosion current of zinc metal anodes. Thanks to these multiple optimizations, these TCh separators effectively suppressed zinc dendrites and side reactions such as HER, thereby significantly improving the cycling stability of zinc anodes (Figure 15l–o).

Moreover, some research teams have also utilized natural macromolecular polymers grafted with polar groups to construct self‐supporting separators, striving to optimize the chemical reactions at the electrode interface more efficiently and further enhance the electrochemical cycling durability of zinc anodes [144, 166, 167, 344]. Yan et al. developed a sulfonated bacterial cellulose (SBC) membrane that could be manufactured on a large scale and display high ionic conductivity and tensile strength, serving as a functional self‐supporting flexible separator for AZIBs (Figure 16a–c) [144]. The sulfonate groups on SBC chains restrained the disordered migration of SO_4_^2‒^ toward zinc anodes through electrostatic repulsion, whilst simultaneously optimizing the transmission pathways of zinc ions to facilitate their rapid transport. Through the combined effects of surface functional groups such as sulfonate groups and fiber network structure, the constructed SBC separators effectively modulated the solvation structure of hydrated zinc ions to promote their desolvation (Figure 16d), while enhancing the transport and deposition kinetics of zinc ions and substantially decreasing SO_4_^2‒^ on zinc anodes (Figure 16e), thereby effectively suppressing zinc dendrites and undesirable side reactions at the electrode interface. Analogously, another research team also reported a functionalized self‐supporting separator composed of sulfonated cellulose (CF‐SO_3_ separator), which had a low thickness and excellent mechanical properties as well as high ion conductivity [167]. This fabricated CF‐SO_3_ separator not only restricted the massive disordered migration of SO_4_^2‒^ to the surface of the zinc metal electrode but also suppressed the 2D diffusion of zinc ions on the electrode surface, enabling uniform deposition of zinc ions while inhibiting by‐product formation and electrode corrosion, thereby yielding dendrite‐free zinc anodes with high cycling stability.

**FIGURE 16:**
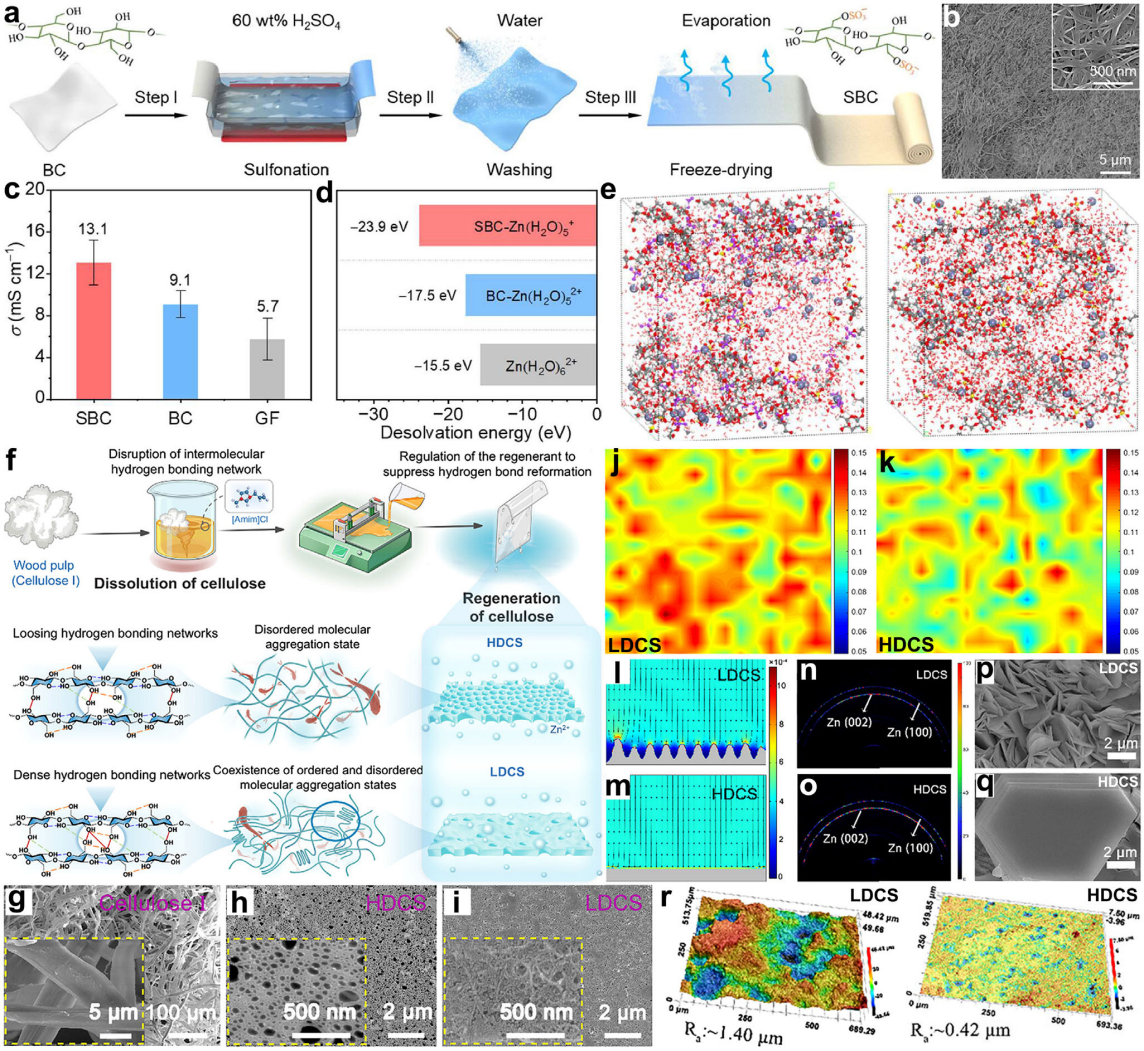
(a) Schematic diagram of the construction of SBC separator. (b) SEM characterizations of the surface of SBC separator. (c) The ionic conductivity of different separators. (d) The desolvation energies of zinc ions regulated by different separators. (e) MD simulations of SBC and BC in ZSO electrolyte. Reproduced with permission [144]. Copyright 2025, Elsevier. (f) Illustration of the construction and structural features of HDCS and LDCS. g‐i) SEM characterizations of different separators. (j, k) Raman characterizations of different separators. (l, m) The simulated concentration distributions of zinc ions on zinc anodes regulated by different separators. (n–r) 2D grazing incidence wide angle X‐Ray scattering patterns, SEM characterizations and CLSM characterizations of the cycled zinc anodes protected by LDCS and HDCS. Reproduced with permission [132]. Copyright 2025, Wiley‐VCH.

In addition, a carbonylation side group‐modified bacterial cellulose (CBC) has also been utilized to construct functionalized self‐supporting separators to optimize the deposition and stripping of zinc ions on zinc anodes [344]. The carbonyl side groups facilitated the release of adsorbed zinc ions from the cellulose chains while enhancing the transport efficiency of zinc ions, thereby enabling these CBC separators to promote uniform zinc ion deposition and inhibit zinc dendrites. Meanwhile, Cui and colleagues developed a hydrophilic self‐supporting membrane constructed from CNFs grafted with zinc sulfonate (CNF‐SO_3_Zn), which exhibited excellent mechanical properties and served as a functional separator to significantly improve the electrochemical performance of zinc anodes [166]. In aqueous batteries, these manufactured CNF‐SO_3_Zn separators with uniform pore structures selectively and efficiently transmitted zinc ions while inhibiting water molecules and SO_4_^2‒^ from corroding zinc anodes, thereby simultaneously suppressing zinc dendrites and harmful side reactions. Consequently, the assembled Zn||Zn symmetric batteries incorporating these CNF‐SO_3_Zn separators displayed stable cycling performance even under conditions of lean‐electrolyte and high deposition areal capacity.

Recently, in order to further optimize the performance of natural macromolecular polymer‐based self‐supporting separators, some functional self‐supporting separators derived from either regenerated natural polymers or a combination of two natural polymers have been progressively developed. Qiu et al. utilized wood pulp as raw material and employed dissolution‐regeneration technique to construct a novel cellulose separator featuring a highly disordered aggregated state structure (Figure 16f) [132]. These constructed highly disordered cellulose separators (HDCS) had a looser H‐bond network compared to the lightly disordered cellulose separators (LDCS), owing to the effective suppression of H‐bond rearrangement by weakly protic solvents during the regeneration process. Simultaneously, the fabricated HDCS displayed a more pronounced porous structure with a more uniform molecular aggregation state (Figure 16g–k). Leveraging these structural advantages, the constructed HDCS promoted the uniform distribution of zinc ions and electric fields at the electrode interface (Figure 16l,m), enabling the uniform deposition of zinc ions along the Zn (002) crystal plane (Figure 16n–q), thereby suppressing zinc dendrites and side reactions (Figure 16r).

In another study, a self‐supporting composite cellulose separator composed of CNFs rich in ‒OH and carboxyl‐rich oxidized CNFs (CTNF separator) was successfully constructed by vacuum filtration (Figure 17a) [42]. These self‐supporting composite separators, formed by the close interweaving of two types of CNFs, featured abundant nanopores and a thickness of merely 28 µm, significantly enhancing the cycling stability of zinc anodes (Figure 17b,c). To be specific, CNFs effectively anchored water molecules through the hydrogen bonding of their –OH groups, whilst the carboxyl‐rich oxidized CNFs (CONFs) enhanced their adsorption of zinc ions through the electrostatic interactions of their negatively charged ‒COOH (Figure 17d). Therefore, the synergistic effect of the two types of CNFs allowed these self‐supporting composite separators to effectively promote the desolvation of zinc ions, reduce the water content at the electrode interface, and significantly optimize the deposition kinetics of zinc ions, thereby effectively suppressing interfacial side reactions and the formation of zinc dendrites (Figure 17e,f).

**FIGURE 17:**
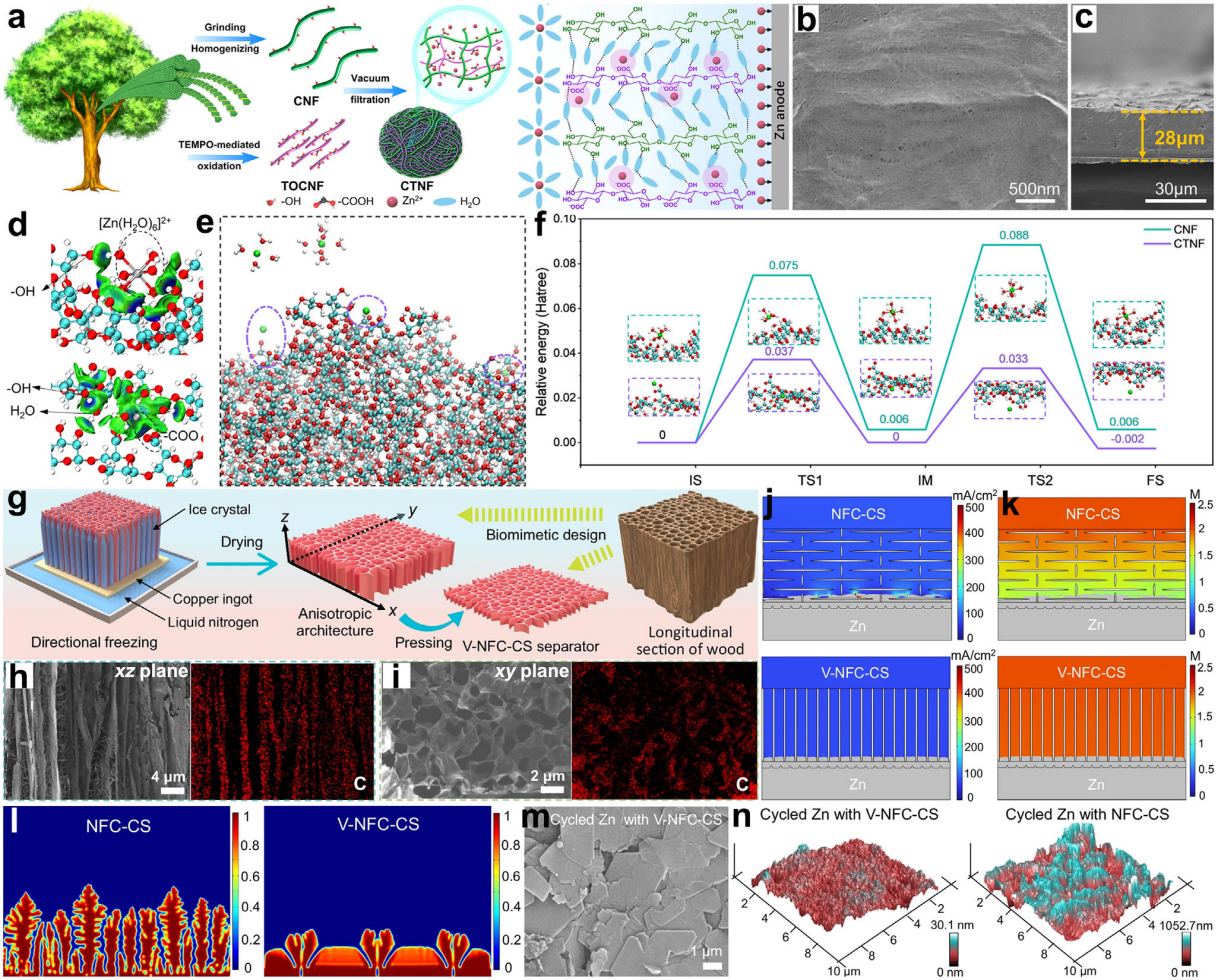
(a) Schematic diagram of construction, structural characteristics and efficacy of CTNF separator. (b, c) SEM characterizations of the surface and cross‐section of CTNF separator. (d) The simulated interactions among ‒OH, ‒COOH, and zinc ions in the solvated structure. (e) The simulated interactions between CTNF separator and electrolyte via MD. (f) The energy variations and corresponding molecular configurations of zinc ions migrating along different CNF chains. Reproduced with permission [42]. Copyright 2025, Elsevier. (g) Schematic depiction of construction of V‐NFC‐CS separator. (h, i) SEM characterizations and corresponding EDS images of the V‐NFC‐CS separator on the *xz* and *xy* planes. (j) The simulated electric field distributions and (k) the simulated concentration distributions of zinc ions on zinc anodes protected by different separators. (l) Deposition morphology simulation of zinc ions on zinc anodes protected by different separators. (m) SEM characterization of the cycled zinc anode protected by V‐NFC‐CS separator. (n) AFM characterizations of the cycled zinc anodes protected by different separators. Reproduced with permission [121]. Copyright 2025, Springer Nature.

In the meantime, Chen and colleagues fabricated a self‐supporting anisotropic separator (V‐NFC‐CS separator) by subjecting a mixed aqueous solution of chitosan and nanofibrillated cellulose (NFC) to directional freezing and further compression, which effectively overcame the problem of poor cycling durability faced by zinc anodes (Figure 17g) [121]. These self‐supporting separators with a thickness of 23 µm contained numerous uniformly distributed channels parallel to the xz plane, exhibiting high ionic conductivity and excellent mechanical properties (Figure 17h,i). Benefitting from their unique structural advantages and outstanding physicochemical properties, these constructed V‐NFC‐CS separators not only homogenized the current density field and the distribution of zinc ions at the electrode interface (Figure 17j,k), but also accelerated the transmission of zinc ions and inhibited their vertical deposition on zinc electrodes (Figure 17l), thus acquiring dendrite‐free zinc anodes while avoiding side reactions (Figure 17m,n). Additionally, a self‐supporting composite cellulose (CSC) membrane constructed by cross‐linking sulphonated cotton fibers (SCFs) and carboxylated sisal fibers (CSFs) has also been employed as a functionalized separator for AZIBs [133]. These self‐supporting CSC separators, with excellent pH tolerance and mechanical properties, optimized the transport of zinc ions and suppressed the activity of interfacial water, contributing to the high cycling stability of dendrite‐free zinc anodes.

Beyond that, compounding inorganic functional material with natural macromolecular polymer is also a significant approach for constructing advanced natural macromolecular polymer‐based self‐supporting separators tailored to optimize the electrochemical performance of zinc anodes. Thereinto, the research on simply physically mixing to jointly construct natural polymer‐based composite self‐supporting separators began earlier, with notable progress achieved in recent years [346, 347, 348, 349, 350, 351, 352]. Owing to the inherent structural advantages of CNFs, they are frequently employed as the framework for composite self‐supporting separators. Yang and collaborators developed a cellulose‐based composite self‐supporting membrane composed of CNFs and LMS, which served as a functionalized separator to enhance the stability of zinc anodes [346]. This hydrophilic self‐supporting separator possessed both high ionic conductivity and high tensile strength, facilitating the desolvation and rapid transport of zinc ions alongside their uniform deposition, thereby achieving suppression of side reactions and zinc dendrites. Analogously, another cellulose‐based self‐supporting separator composed of CNFs and HNTs has been employed to protect zinc anodes also eliminating zinc dendrites and side reactions on zinc anodes by promoting the desolvation and uniform deposition of zinc ions [347]. Concurrently, fly ash was also utilized in conjunction with CNFs to construct self‐supporting cellulose‐based composite separators aimed at optimizing zinc anodes [348]. The incorporation of fly ash endowed the constructed cellulose‐based composite separator with higher ionic conductivity compared to pure cellulose separators, while more effectively promoting the desolvation of zinc ions. Moreover, fly ash particles induced zinc ions to selectively deposit uniformly along the Zn (002) crystal facet. Consequently, the constructed cellulose‐based composite separator effectively suppressed water‐induced side reactions and zinc dendrites, achieving performance optimization of zinc anodes.

In addition, Ying et al. constructed a hydrophilic self‐supporting composite separator composed of boehmite (BM) rich in polar groups and bacterial cellulose (BC), featuring excellent flexibility and high mechanical strength, which effectively improved the cycling performance of zinc anodes (Figure 18a–c) [349]. Specifically, the strong coordination interactions between polar groups such as ‒OH on BM and BC chains with water molecules enabled the constructed BM/BC separators to not only promote the desolvation of hydrated zinc ions but also disrupt the hydrogen‐bond network between water molecules, thereby inhibiting the activity of interfacial water and the generation and transport of protons (Figure 18d–f). Meanwhile, the competitive adsorption of zinc ions by BM and BC chains enabled zinc ions to undergo rapid adsorption and desorption, facilitating their swift transport and uniform distribution at the electrode interface (Figure 18g). Additionally, BM and BC chains anchored SO_4_^2‒^ through a mutual ‘water bridge’ effect, preventing the aggregation of SO_4_^2‒^ at the electrode interface (Figure 18h,i). Benefiting from the synergistic optimization of BM and BC, these BM/BC separators effectively suppressed water‐induced side reactions and zinc dendrites, eliminated byproducts (Figure 18j,k), and enabled Zn||Zn symmetric batteries incorporating them to achieve stable cycling for 2000 h at 1 mA cm^−2^/1 mAh cm^−2^.

**FIGURE 18:**
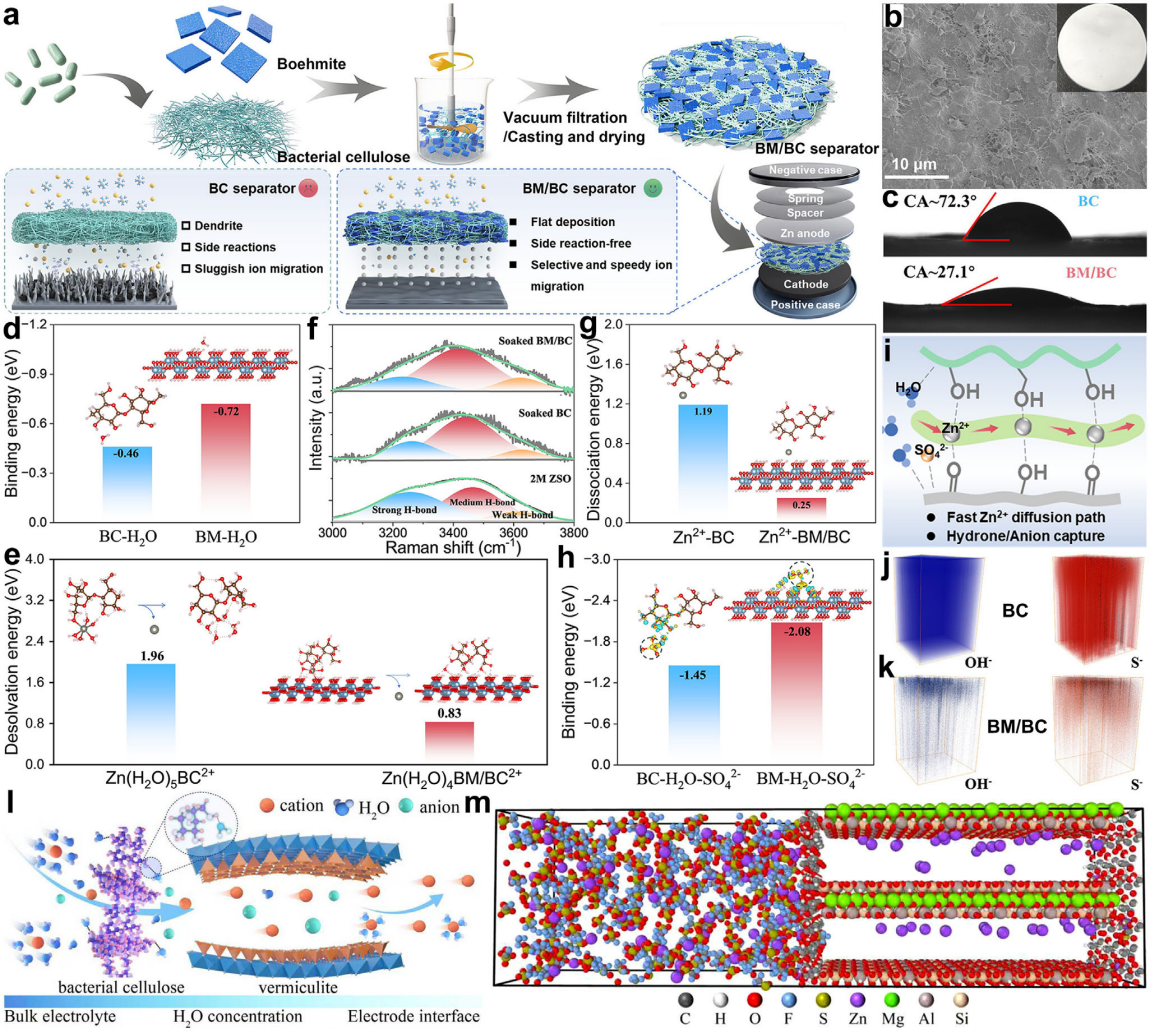
(a) Schematic diagram of the construction of the BM/BC separator and its optimization effect on zinc anodes. (b) SEM characterization and optical image of the BM/BC separator. (c) Wetting angles of BM/BC and BC separators. (d) The binding energies of H_2_O with BC and BM. (e, g) The dissociation energies of hydrated zinc ions and free zinc ions detaching from BC and BM/BC. (f) Raman characterizations of ZSO electrolyte and the soaked separators. (h) The binding energies of SO_4_^2−^ with BC‐H_2_O and BM‐H_2_O structures. (i) Mechanism diagram of transport of zinc ions in BM/BC separator. (j, k) TOF‐SIMS results revealing the spatial distribution of S^‒^ and OH^‒^ fragments on zinc anodes after cycling protected by BC and BM/BC separators. Reproduced with permission [349]. Copyright 2025, American Chemical Society. (l) Mechanism diagram of BC/V separator regulating the transmission of solvents and ions. (m) MD simulation diagram of selective ion transport regulated by the BC/V separator. Reproduced with permission [350]. Copyright 2025, Elsevier.

Furthermore, some researchers have also explored constructing composite separators with specific functions by integrating natural macromolecular polymer fibers as a framework with functional inorganic materials to optimize zinc anodes [148, 350, 351, 352]. Huang et al. fabricated a self‐supporting composite separator with biomimetic functionality by using BC fibers as a skeleton to composite 2D vermiculite (V) [350]. The constructed hydrophilic self‐supporting BC/V separator featured excellent thermal stability and mechanical properties, and demonstrated an ion sieving effect similar to enzyme gating, which significantly improved the performance of zinc anodes (Figure 18l). Notably, the BC fiber network and the abundant micro‐ and nano‐pores formed in conjunction with 2D V facilitated the desolvation of hydrated zinc ions while confining water molecules. Simultaneously, 2D V selectively transported zinc ions through its enzyme‐gated effect (Figure 18m). As a result, the synergy of these two components enabled these BC/V separators to optimize the transport kinetics and deposition uniformity of zinc ions while reducing water molecules at the electrode interface, thus suppressing zinc dendrites and water‐induced corrosion side reactions. Meanwhile, cellulose‐based Janus composite separators with distinct compositions and properties on each side have also been developed for AZIBs. For instance, Cao and colleagues constructed a self‐supporting Janus composite separator by simply vacuum‐filtering silver nanowires (Ag NWs) onto a BC membrane [351]. Thanks to the uniformly distributed Ag NW network on one side of the separator, which provided high electrical/thermal conductivity and an ionic pumping effect, these Janus Ag NWs/BC separators exhibited superior mechanical properties and significantly higher ionic conductivity compared to the BC separators. Moreover, the Ag NW network adjacent to zinc anode induced uniform distribution of the electric field at the electrode interface, [257, 351] promoting the uniform deposition of zinc ions on zinc anodes. Bolstered by the aforesaid advantages, the assembled Zn||Zn symmetric batteries incorporating these Janus Ag NWs/BC separators demonstrated stable cycling performance exceeding 1000 h at a current density as high as 80 mA cm^−2^.

In another research, a self‐supporting cellulose‐based Janus separator with one side coated with a layer of C/Cu nanocomposite derived from metal‐organic frameworks (MOF) was also constructed via vacuum filtration to overcome issues such as dendrites faced by zinc anodes [352]. On one hand, the CNF membranes with uniform nanopores and high mechanical strength sieved and facilitated the transport of zinc ions. Also, owing to the high specific surface area and abundant zinc‐affinity sites distributed throughout the C/Cu nanocomposite layer, its placement near zinc anodes effectively optimized the deposition kinetics of zinc ions, enabling uniform deposition of zinc ions and thereby effectively inhibiting zinc dendrites. Recently, Wu and collaborators developed a self‐supporting BC‐based Janus separator with a negatively charged surface and a gradient of hydrophobicity using a controllable gradient SiO_2_ impregnation approach [148]. One side of this gradient‐hydrophobic BC separator (gBC) was loaded with a large number of SiO_2_ particles, exhibiting strong hydrophobicity, while the other side exposed the BC fiber network, displaying hydrophilicity (Figure 19a,b). The gradient impregnation of SiO_2_ endowed the gBC separators with superior mechanical properties compared to the BC separators and enabled the gBC separator to effectively promote the desolvation of hydrated zinc ions (Figure 19c,d). Concurrently, the negatively charged groups on the surface of the gBC separator homogenized the distribution of zinc ions at the electrode interface while resisting corrosion by chloride ions. These beneficial effects synergistically enhanced the cycling durability of zinc anodes paired with the gBC separator, resulting in a smoother zinc anode after cycling (Figure 19e,f). Notably, the assembled Zn||Zn symmetric batteries with the gBC separator demonstrated outstanding cycling stability even under high current densities and deep discharge conditions (Figure 19g,h).

**FIGURE 19:**
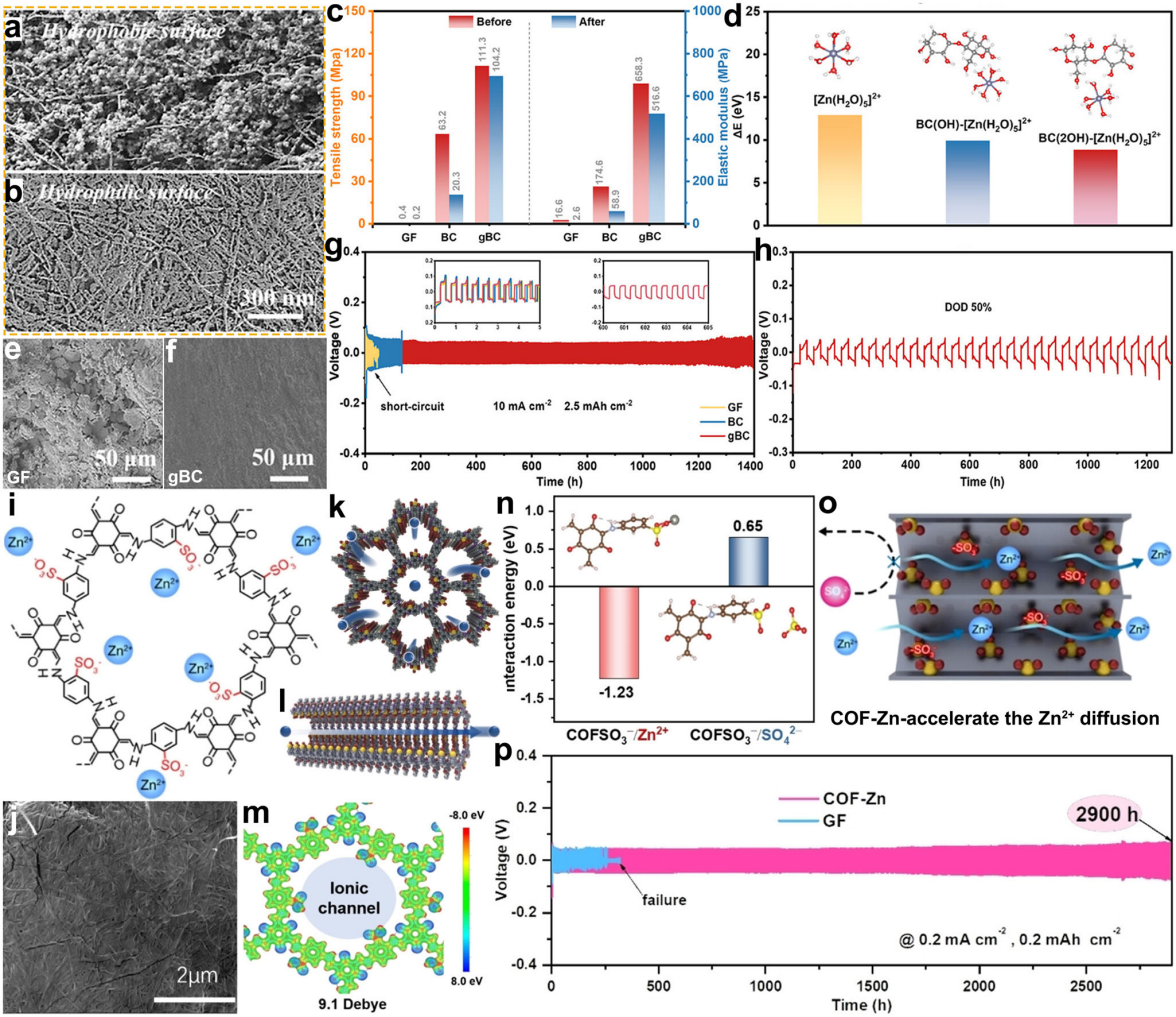
(a, b) SEM characterizations of the hydrophobic and hydrophilic sides of the gBC separator. (c) Comparison of mechanical properties of different separators before and after expansion in water. (d) The desolvation energy barriers of hydrated zinc ions under different circumstances. (e, f) SEM characterizations of the cycled zinc anodes protected by the GF and gBC separators. (g) The cycle performance of the assembled Zn||Zn symmetric batteries using different separators. (h) The cycling performance of Zn||Zn symmetric battery with the gBC separator under deep discharge conditions. Reproduced with permission [148]. Copyright 2025, Wiley‐VCH. (i) Molecular ring of the synthesized COF‐Zn. (j) SEM characterization of COF‐Zn membrane. (k, l) Vertical view and side view of the transport of zinc ions in stacked COF‐Zn along the stacking direction. (m) ESP of COF‐SO_3_^‒^. (n) The interaction energies of COFSO^3−^ with zinc ions and SO_4_^2‒^. (o) Schematic diagram of the selective transport of zinc ions in COF‐Zn. (p) The cycling stabilities of Zn||Zn symmetric batteries using different separators. Reproduced with permission [354]. Copyright 2025, Wiley‐VCH.

What's more, some novel functional materials featuring large specific surface areas and porous structures, such as MOFs and covalent organic framework (COF) materials have also been integrated into cellulose fiber networks to fabricate a superior composite separator. In 2023, an amino‐modified Zr‐based MOF material (UiO‐66‐NH_2_) was blended with lignocellulose (LC) to construct a low‐thickness self‐supporting composite membrane with high ionic conductivity via vacuum filtration, which, as a functionalized separator, improved the electrochemical stability of zinc anodes [353]. Because the channels contained numerous ‒NH_2_ groups with favorable zinc affinity for zinc ions and strong interactions with water molecules, these UiO‐66‐NH_2_ materials not only accelerated the desolvation of hydrated zinc ions but also facilitated the efficient migration, uniform distribution and deposition of zinc ions. Meanwhile, the LC network endowed the constructed LC@UiO‐66‐NH_2_ separator with excellent flexibility and high mechanical strength. Consequently, these LC@UiO‐66‐NH_2_ separators effectively suppressed side reactions such as HER and zinc dendrites. Xu and cooperators mixed the synthesized COF material grafted with Zn^2+^‐substituted sulfonic acid groups (COF‐Zn) with the BC fibers and then subjected them to reduced‐pressure filtration to obtain a novel self‐supporting composite COF‐Zn membrane (Figure 19i,j) [354]. COF‐Zn selectively transported dehydrated zinc ions through its channels, whilst the abundant negatively charged sulfonate groups within these channels repelled SO_4_^2‒^, optimizing the transport kinetics of zinc ions and reducing the concentration of SO_4_^2‒^ and water molecules migrating to the anode interface (Figure 19k–o). On the other hand, the hydrophilic BC fibers trapped active water molecules, lowering the activity of interfacial water. Benefiting from the synergistic optimization of the two components, zinc dendrites, and corrosion side reactions have been suppressed, enabling zinc anodes to achieve long‐term cycling durability exceeding 2900 h in Zn||Zn symmetrical batteries with the COF‐Zn separators (Figure 19p).

Also, some researchers have concurrently employed both conventional polymers and inorganic materials to jointly optimize the performance of cellulose separators. A self‐supporting Janus composite membrane comprising three components—CNFs, PVA, and ZrO_2_—was fabricated via vacuum filtration and employed as a functionalized separator to enhance the stability of zinc anodes [355]. Among the three components, PVA acting as the binder enhanced the stability and mechanical properties of the CNF network, while the CNF network and ZrO_2_ layer jointly optimized the transmission channel of zinc ions. And, the ZrO_2_ layer with a high dielectric constant homogenized the interface electric field, inducing the uniform deposition of zinc ions. Thus, the constructed CNF/PVA/ZrO_2_ separator was more effective in suppressing zinc dendrites compared to the CNF separators, achieving long‐term stable cycling of zinc anodes.

More interestingly, the in situ anchoring and chemical bonding of inorganic functional materials or synthetic polymers onto natural macromolecular polymer fibers demonstrated a marked enhancement of the mechanical and electrochemical properties of natural polymer‐based separators [265, 356, 357, 358, 359]. Earlier, Wang and collaborators demonstrated that a self‐supporting cellulose‐based composite separator composed of TEMPO‐oxidized CNFs (TCNFs) coated with amorphous Zr‐O layers (Zr‐CNF separator), constructed via in situ hydrolysis of Zr^4+^, more effectively enhanced the electrochemical performance of zinc anodes than TCNF separator [356]. It was found that the chemical cross‐linking of TCNFs by Zr^4+^ and the subsequently formed Zr–O coating layer endowed the constructed Zr‐CNF separator with a stable porous structure and excellent anti‐swelling properties. At the same time, the amorphous Zr–O coating layer induced a uniform oriented electric field around the separator that could accelerate the migration of zinc ions, enhancing the deposition kinetics and uniformity of zinc ions. Precisely due to these optimizations conferred by the Zr–O coating layer, the zinc metal electrodes assembled with the Zr–CNF separators within the symmetric batteries achieved stable cycling for up to 680 h at high current densities and deposition areal capacities.

Thereafter, a self‐supporting cellulose‐based composite separator composed of BC fibers anchored with BaTiO_3_ nanoparticles (BTO NPs) was also demonstrated to exhibit superior efficacy in stabilizing zinc anodes [265]. The BTO NPs with a high dielectric constant, evenly dispersed on BC fibers, enhanced the vertical component of the local electric field around them, empowering the constructed BC‐BTO separator to effectively induce the vertical diffusion of zinc ions and promote their uniform deposition on the electrode surface, while repelling SO_4_^2‒^ from migrating toward zinc anodes. Therefore, the multiple functions of the constructed BC‐BTO separator effectively suppressed zinc dendrites and corrosion side reactions, achieving long‐term cycling stability of zinc anodes. Moreover, He et al. utilized spontaneous hydrogen bonding to composite hydroxyapatite nano particles (HAP) with BC fibers, constructing a self‐supporting composite membrane (ZnHAP/BC membrane) [357]. These fabricated composite membranes, when applied as functional separators in AZIBs, not only accelerated the desolvation of hydrated zinc ions and restricted the activity of water molecules through the abundant oxygen‐containing groups on BC chains, but also enhanced the transport kinetics of zinc ions and their uniform dispersion on the electrodes through HAP, which has ionic conductor properties, effectively suppressing side reactions and zinc dendrites on zinc anodes. Accordingly, these zinc anodes protected by the ZnHAP/BC separators demonstrated high cycling stability.

In comparison with the foregoing several inorganic materials, a few single nonmetallic crystals, polymers, and organic compounds with higher chemical activity either formed more functional chemical bonds or generated stronger interactions when composited or bonded with natural macromolecular polymeric fibers such as cellulose. Recently, boron (B) powder has been used in conjunction with CNFs to construct a self‐supporting boron‐integrated cellulose‐based separator (B/CNF separator) that effectively overcame the interface stability issue of zinc anodes (Figure 20a,b) [358]. It was worth noting that the B─O─C and B─O bonds generated by the mutual combination of B powder and CNFs provided active Lewis base sites for coordinating zinc ions, inducing the rapid desolvation of hydrated zinc ions and facilitating the directional and efficient migration of dehydrated zinc ions (Figure 20c). Meanwhile, the uniformly dispersed B particles homogenized the interfacial electric field, effectively suppressing local charge accumulation at the electrode interface. Owing to these synergistic optimization effects, the constructed B/CNF separators effectively inhibited side reactions and zinc dendrites, significantly improving the electrochemical stability of zinc anodes. In another study, a self‐supporting cellulose‐based composite separator (TOCN‐A separator) composed of TEMPO‐oxidized CNFs (TOCN) modified by anionic PAM (APAM) through strong hydrogen bonding has been proven to optimize the electrochemical stability of zinc anodes more effectively than TOCN separator (Figure 20d) [359]. Benefiting from the strong H‐bonds formed between TOCN and APAM, coupled with the incorporation of negatively charged groups such as ‒COOH, these constructed TOCN‐A separators exhibited enhanced mechanical strength and surface electronegativity as well as improved affinity for zinc ions. On the one hand, the pronounced zinc affinity endowed these TOCN‐A separators to facilitate the desolvation of hydrated zinc ions and improve the transport kinetics and deposition uniformity of zinc ions. On the other hand, the increased surface electronegativity enabled these TOCN‐A separators to effectively restrict the migration of SO_4_^2‒^ towards zinc anodes through electrostatic repulsion. Consequently, by integrating these advantageous functions, these TOCN‐A separators effectively inhibited the generation of zinc dendrites and byproducts, thereby facilitating long‐term cycling stability of zinc anodes.

**FIGURE 20:**
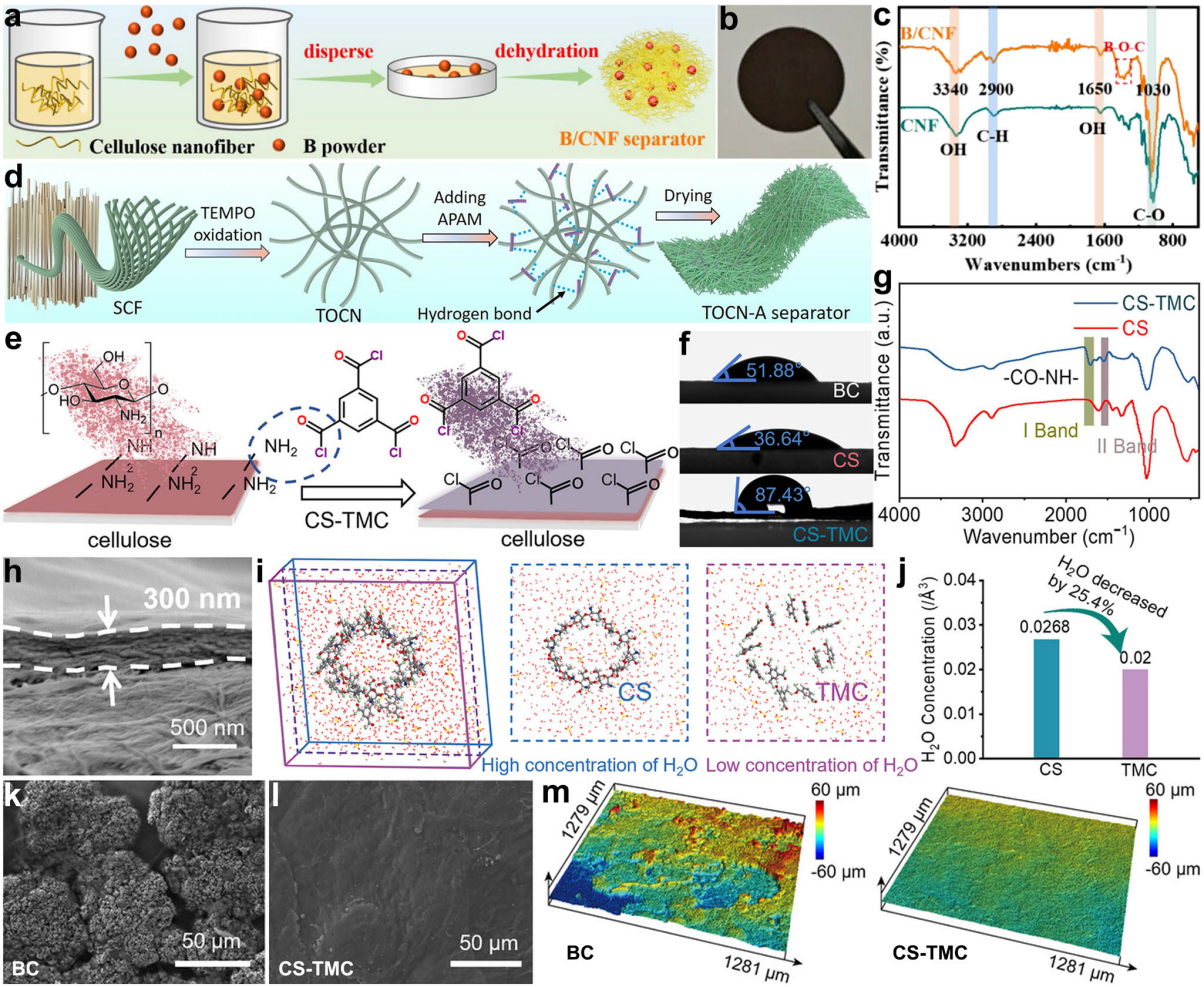
(a) Illustration of the fabrication of B/CNF separator. (b) Digital photograph of B/CNF separator. (c) Fourier‐transform infrared (FTIR) spectrums of different separators. Reproduced with permission [358]. Copyright 2025, Wiley‐VCH. (d) Schematic diagram of the preparation of the TOCN‐A separator. Reproduced with permission [359]. Copyright 2025, Springer Nature. (e) Schematic diagram of the synthesis of CS‐TMC layer. (f, g) Wetting angles and FTIR spectrums of different separators. (h) SEM characterization of the cross‐section of TCS‐TMC separator. (i) MD 3D simulation of the CS/TMC interface and corresponding screenshots for CS and TMC. (j) Comparison of H_2_O concentration within the different regions. (k–m) SEM characterizations and CLSM images of the cycled zinc anodes protected by BC and CSTMC separators. Reproduced with permission [100]. Copyright 2025, Wiley‐VCH.

In addition, Han and collaborators employed an interface tandem chemical design strategy to chemically crosslink a chitosan‐trimesoyl chloride (CS‐TMC) layer on BC membrane, constructing a self‐supporting composite separator (CS‐TMC separator) with excellent wettability and absorption rate for the electrolyte (Figure 20e,f) [100]. Despite the CS‐TMC layer formed by the successful crosslinking of chitosan and trimesoyl chloride (TMC) being extremely thin (∼ 300 nm), it markedly enhanced the electrochemical performance of BC separators in AZIBs (Figure 20g,h). The cross‐linked TMC bearing hydrophobic groups such as benzene rings and acyl chlorides enabled the fabricated CS‐TMC separators to promote the desolvation of zinc ions while reducing the concentration of active water at the electrode interface, thereby effectively suppressing active water‐induced side reactions. Concurrently, the chitosan polymer chains, crosslinked with the BC membrane, were rich in zinc‐affinity ‒OH, enhancing the transport efficiency of zinc ions within the CS‐TMC separators and boosting the uniform deposition of zinc ions (Figure 20i,j). Leveraging the synergistic effects of each component, these CS‐TMC separators more significantly suppressed corrosion side reactions and zinc dendrites compared to the BC separators, more effectively ensuring the surface flatness of zinc anodes after cycling (Figure 20k–m).

To more intuitively compare and analyze the effectiveness of functionalized separators constructed from different natural polymers in optimizing zinc anodes, a radar diagram as shown in Figure 21a was established based on three key electrochemical parameters, which clearly demonstrates the electrochemical performance differences of Zn||Zn symmetrical batteries assembled based on various natural polymer‐based functional separators. From the perspective of industrial implementation, natural polymer‐based composite separators that use standardized materials such as commercial GF separators as their main frameworks currently appear to be more practical, as their manufacturing processes are simpler, they are more compatible with existing manufacturing technologies, and product consistency is easier to control. On the other hand, it is easy to find that cellulose with better film‐forming properties has been more widely used to construct self‐supporting functionalized separators to optimize the cycling stability of zinc anodes. In contrast, chitin, chitosan, lignin, and alginate, due to their insufficient mechanical strength, limited chemical stability, inadequate ionic conductivity, or potential dissolution/swelling in weakly acidic electrolytes (pH<6.5), have been employed alone at a lower frequency as frameworks for fabricating self‐supporting functionalized separators suitable for AZIBs. Nevertheless, the inherent degradability, tunable mechanical properties, high processability, and abundant surface functional groups of these natural macromolecular polymers make them highly attractive candidate materials for constructing functional separators that can optimize the stability of zinc anodes. Fabricating novel functional composite separators with better performance by integrating the advantages of different natural polymers through the combined use of multiple natural polymers may become a future research direction. Although this prediction remains uncertain, the exploration of developing advanced separators based on these natural polymers will persist, ultimately strongly driving the advancement of AZIBs.

**FIGURE 21:**
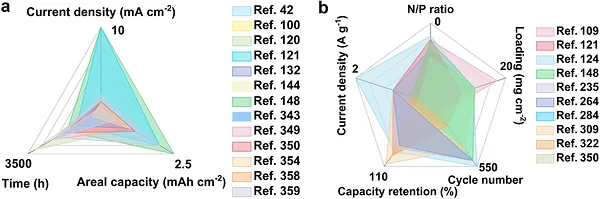
(a) Electrochemical performance comparison of Zn||Zn symmetric batteries with different natural polymer‐based functional separators. (b) Cycling performance comparison of the full batteries optimized with natural polymers under the corresponding test conditions.

Furthermore, in order to more effectively evaluate the practicality of natural polymer‐based zinc metal anodes optimized by different strategies (artificial SEI layer, electrolyte optimization, functional separators), we carefully analyzed the electrochemical performance of zinc‐ion full batteries assembled with these functionalized zinc anodes under specific test conditions, such as negative/positive (N/P) ratio and electrode material loading, and so on. The analysis results showed that the practicality of natural polymer‐based zinc metal anodes and zinc‐ion full batteries assembled with them still needed further improvement (Figure 21b). Thereinto, the N/P ratio can effectively reveal the zinc utilization in AZIBs, while the electrode material loading is the key factor determining their actual energy densities, cumulative capacities and service lives. The higher the electrode material loading and the closer the N/P ratio is to 1, the more it can reflect the practicality of the battery. Currently, testing natural polymer‐based zinc‐ion full batteries under conditions of high electrode material loading to assess their practicality has been adopted by numerous studies. However, the N/P ratio, as another important testing parameter, has been underutilized in the electrochemical performance evaluation of these batteries and requires further promotion to more comprehensively assess the practicality of natural polymer‐based zinc anodes and corresponding AZIBs that continue to be developed in the future.

## 4 Conclusions and Perspectives

In summary, applying natural macromolecular polymers to construct artificial SEI layers, optimizing electrolytes, and developing functionalized separators can effectively address issues such as corrosion, passivation, and zinc dendrites encountered by zinc metal anodes, significantly enhancing the stability of zinc anodes and thereby improving the electrochemical performance of AZIBs. The pronounced efficacy of natural polymers in optimizing zinc anodes stems primarily from their unique structural and surface properties, including multi‐level self‐assembled chain/network architectures, superior mechanical characteristics, self‐assembly capacity, modifiability, processability, and chemical multifunctionality conferred by multiple functional groups. Moreover, their inherent characteristics of low cost, readily availability, renewability, environmental friendliness, and biodegradability render the optimization strategies for zinc anodes developed based on this type of materials highly sustainable and industrially viable, providing a green and sustainable development pathway for energy storage and conversion powered by AZIBs. In this review, the multiple structure‐function relationships between the structural/chemical properties of various natural polymers and their optimization effect on the stability of zinc anodes are revealed, and five regulation mechanisms oriented by structural/surface features are effectively elucidated from microscopic and mesoscopic levels. By sorting out and analyzing a series of research advances aimed at optimizing zinc anodes using natural polymers from aspects such as constructing artificial SEI layers, optimizing electrolytes, and designing functionalized separators, this review uncovers the interplay and mutually guiding relationship among structural features/surface chemical properties, zinc anode optimization strategies, and regulatory mechanisms, delivering important inspiration for the rational design of regulatory strategies driven by natural polymers that can better optimize zinc anodes.

In despite of significant advances have been made in optimizing the stability of zinc anodes utilizing natural polymers, effectively enhancing the electrochemical performance of AZIBs, the large‐scale application of these natural polymers in addressing the interface stability issues of zinc anodes still faces some critical challenges due to their certain inherent undesirable properties (Figure 22).

**FIGURE 22:**
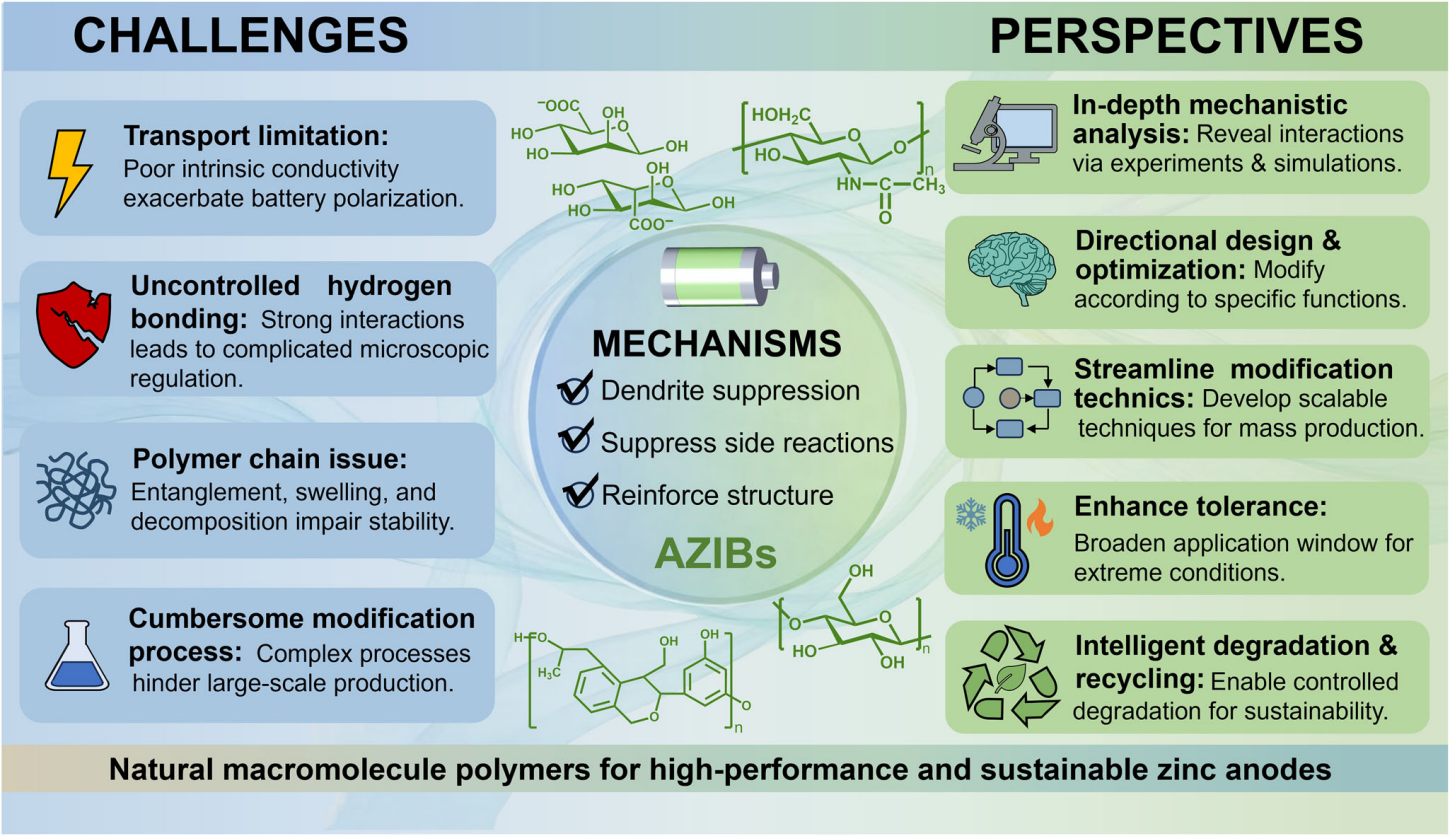
Challenges faced by natural macromolecular polymers in optimizing zinc anodes and related future research prospects.

### 4.1 Electrical/Ionic Transport Limitations Exacerbate Battery Polarization

Natural polymers are polysaccharides and their derivatives or aromatic polymers, derived from abundant biomass. They possess linear or quasi‐linear chain structures with numerous polar groups distributed on their surfaces, which provide significant structural and surface advantages for optimizing zinc anodes. However, most natural macromolecule polymers exhibit poor intrinsic electronic conductivity and insufficient ionic conductivity, and their incorporation often increases the internal electronic and ionic transport resistance within AZIBs. The sluggish electron conduction and inefficient ion transport cause a decrease in the electrode reaction rates and slow down the conversion efficiency of internal substance in AZIBs, thereby exacerbating battery polarization and diminishing its electrochemical performance.

### 4.2 Uncontrolled Molecular Hydrogen Bonding Interactions Diminish the Microstructural Controllability of Battery Components

Natural polymers are rich in polar functional groups and possess excellent film‐forming properties, rendering them promising for constructing functional layered structures such as artificial SEI layers, gel electrolytes, or separators. However, the strong hydrogen bonding interactions between the molecular chains of these natural polymers readily lead to high crystallinity during processing, making it challenging to precisely control microstructural parameters such as pore size, porosity, fiber distribution, and thickness uniformity of the layered structures. Moreover, during the solvent removal process (such as drying), the capillary forces between nanofibers or molecular chain segments are prone to cause irreversible collapse of the pore structure, resulting in a densified structure. This not only impedes the effective penetration of electrolytes and the rapid transmission of ions at the electrode interface, but also restricts the interfacial compatibility and structural stability of functional layered structures (e.g., gel electrolytes, functional separators or artificial SEI layers), thereby compromising the rate performance and cycle life of AZIBs.

### 4.3 Entanglement, Swelling, and Decomposition of Polymer Chains Impair Ionic Transport Efficiency and Structural Stability

Although the abundant polar groups on natural polymer chains are conducive to ion conduction, the interaction between them would also promote the aggregation or entanglement of these molecular chains. In AZIBs, hydrated zinc ions and anions may enhance this aggregation and entanglement effect via specific coordination or hydrogen bonding interactions, generating complex network structures with clogged pores and lacking ion transport channels, which hinders the efficient transmission of zinc ions while reducing the mechanical strength of the entire network structure. In addition, some natural polymers exhibit insufficient chemical stability in aqueous environments, swelling or degrading in weakly acidic aqueous electrolytes, which is bound to cause damage the structural integrity of the artificial SEI layers, gel electrolytes and separators based on these natural polymers. Meanwhile, this structural deterioration will further disrupt the existing ion transport channels, hindering the efficient transmission of zinc ions and resulting in diminished cycling stability of AZIBs.

### 4.4 Cumbersome Modification Hinders the Large‐scale Production of Natural Polymers

The vast majority of modification processes for natural polymers remain confined to the laboratory stage due to their relative complexity, failing to meet the requirements of simplified procedures and low cost by large‐scale manufacturing. Concurrently, the multi‐step treatments inherent in complex modification processes inevitably cause batch‐to‐batch differences, such as variations in polymer chain length distribution and functional group content. These differences are highly likely to significantly alter the hydrophilicity/zinc affinity, mechanical properties, and surface chemical activity of natural polymers across different batches, which further affect the consistency of their electrochemical performance when utilized to optimize zinc metal anodes, thereby undermining their viability for large‐scale application in AZIBs. Consequently, existing complex modification processes significantly constrain the large‐scale production of natural polymers with consistent performance.

To effectively advance the large‐scale application of natural polymers in optimizing the cycling stability of zinc anodes and ultimately achieve the industrialization of natural polymer‐based AZIBs, in addition to addressing the aforementioned challenges, it is also necessary to leverage the structural and surface advantages of natural polymers and integrate novel technologies and concepts to conduct in‐depth research. Regarding potential future research on this topic, we have delivered the following prospects (Figure 22):

### 4.5 In‐Depth Mechanistic Analysis Based on Experiments and Theoretical Simulations

Revealing the interaction mechanisms between natural polymer chains and their reaction mechanisms with cations and anions, solvents, and zinc anodes is of great guiding significance for the construction of functional natural polymer‐based materials with excellent electrochemical performance. To comprehensively uncover these complex mechanisms, relevant studies need to combine advanced characterization techniques such as in situ Raman/FTIR spectroscopy and cryo‐electron microscopy with MD simulations and machine learning, to observe, simulate, and deeply analyze the interactions between polymer chains, as well as the electrochemical reactions between polymer chains and ions, solvents, and electrodes, with ions, solvents, and electrodes, along with the evolution of electrode interfaces during charge and discharge. Thereinto, in situ Raman/FTIR spectroscopy is an important and advanced characterization technique for tracking the dynamic changes in the physical and chemical properties of the electrode interface during the charging and discharging process. By applying these in situ characterization techniques to compare and analyze in detail the differential variations in the H‐bond networks, Zn^2+^ solvation structures, types and generation amounts of by‐products at the interface of zinc anodes with and without the application of natural polymers during the electrochemical reaction process, it is expected to clarify the relevant natural polymer‐oriented mechanism of electrode interface evolution. Moreover, once the mechanism of the hydrogen bonding interaction between polymer chains, the folding, entanglement and orientation change rules of polymer chains, and the influence mechanism of polymer chain conformation on the ion channels are elucidated, it will help to directionally regulate the strength of H‐bond interaction between polymer chains according to demand, thereby controlling the crystallinity of polymer chains and ultimately achieving effective regulation of parameters such as pore size and porosity within the layered structure of polymer chain textures. Meanwhile, the revelation of relatively complex reaction mechanisms such as the mutual influence laws among polar functional groups on polymer chains and their synergistic regulation mechanisms on the ion transport in electrolytes and the stability of electrode interfaces would provide clear guidance for constructing polymer‐based functional materials that could most effectively optimize the stability of zinc anodes and the overall electrochemical performance of AZIBs.

### 4.6 Directional Design and Optimization of the Structure and Properties

A database encompassing the physical and chemical properties of various natural polymers should be established through a detailed analysis of their composition, structure, and surface characteristics. In particular, it's recommended to focus on and systematically document the mechanical properties and antiswelling performance, which are key features of natural polymers. So far, only a few existing related studies have evaluated the antiswelling property of natural polymers in real electrolyte environments. Future related research should systematically analyze the dynamic change laws of the swelling rate, dimensional stability, pore structure and size, and mechanical strength of natural polymers during the long‐term electrochemical process, in order to provide more direct guidance for designing natural polymer‐based SEI layers/functionalized separators with persistent structural integrity and stable ionic transport channels. Typically, based on the distinct properties and characteristics of different natural polymers, one or more of the strategies such as physical compounding, hybrid crosslinking, chemical anchoring and chemical coating should be rationally selected to construct natural polymer‐based materials with target properties and functions such as high electronic/ionic conductivity, anti‐swelling, high mechanical strength/flexibility, antidissolution, and high acid/alkali resistance. Depending on the requirements, the materials selected to be used together with natural polymers for constructing functional composites can be highly conductive graphene/carbon nanotubes/polymers, hydrophobic zinc affinitive tensile‐resistant tensile inorganic/organic materials, or even one or several other natural polymers. Moreover, the morphology and structure of functional natural polymer‐based materials could be customized by choosing appropriate processing methods (such as gelation, crosslinking, spin‐coating, electrospinning/spraying, and 3D printing, etc.) or even developing a new method, while taking into account the intrinsic properties and structures of the natural polymers being used.

### 4.7 Streamline the Modification Technics to Mass‐produce Functional Natural Polymers

Due to inevitable impurity interference and molecular conformation differences, even natural polymers extracted directly from biomass using the same process exhibit significant interchain variations, such as inconsistencies in structure and surface properties. Directly applying these heterogeneous raw natural polymers to optimize zinc anodes results in inconsistent and uncontrollable optimization effects, while also hindering the accurate establishment of structure‐electrochemical property relationships. Therefore, it is crucial to modify the original natural polymers to improve the uniformity of their structural and surface properties across polymer chains. Although current modification processes can achieve this goal in the laboratory, the complex procedures make it impossible to ensure consistency in the structure and surface properties of functionalized natural polymers across different batches when scaled up for manufacturing. Moreover, the commonly used chemical treatment processes such as multi‐step grafting and crosslinking not only require strict control of reaction conditions but also consume a large amount of expensive chemical reagents such as crosslinking agents. This leads to the high production cost of natural polymers and the inevitable probability of environmental pollution during their production process, offsetting the original advantages of natural polymers such as low cost and environmental friendliness. Therefore, there is an urgent need to streamline processing and modification techniques, develop simple processing techniques based on physical modification methods such as dry/wet heat treatment, freeze‐thaw treatment, ultrasonic treatment, microwave treatment and mechanical ball milling, to meet the requirements of large‐scale production of functionalized natural polymers, thereby enabling their scalable application in optimizing zinc anodes.

It is recommended that cross‐experimental validation be combined with artificial intelligence (AI)/machine algorithm simulation prediction to simultaneously advance this arduous challenge of streamlining the processing and modification techniques of natural polymers. Specifically, by first correlating the raw material type, molecular weight, surface group type and chain structure information of natural polymers with their performance indicators such as ionic conductivity, Zn^2+^ transfer behavior, antiswelling property, interfacial impedance and electrochemical cycling stability, and then correlating them with relevant processing parameters (temperature, ultrasonic intensity, microwave power and ball‐milling speed, etc.), AI simulation or machine learning respectively establish two sets of mapping relationship models. Then, relying on AI simulation/machine learning again, these two sets of models are further integrated and optimized to screen out the processing parameter ranges corresponding to the excellent performance indicators of natural polymers. Concurrently, these theoretical predictions should be further validated through processing experiments and performance tests on natural polymers, with the ultimate goal of identifying the optimal modification technics. Whilst these tasks involve a substantial workload, they hold great significance for advancing the industrial application of natural polymer‐based AZIBs.

### 4.8 Enhance Tolerance to Extreme Conditions and Broaden the Application Window

Enhancing the adaptability and tolerance of natural polymer‐based AZIBs to extreme conditions will positively advance their industrial application. To achieve this goal, developing functionalized natural polymer‐based materials capable of maintaining stable electrochemical performance under extreme conditions, including extreme temperatures, low electrolyte content, and high current densities, has become crucial. Owing to their unique H‐bond networks and flexible molecular chain structures, natural polymers have an inherent potential in constructing functional polymer materials that can withstand extreme temperatures. It is advised that the structure and concentration of the H‐bond network between polymer chains be designed and regulated with the assistance of in situ characterization and theoretical simulation, so that the polymer chains can maintain dynamic bonding between chain segments at both low and high temperatures, ensuring structural integrity and toughness, and thereby achieving good electrochemical performance of the constructed functional polymer materials over a wide temperature range. In addition, it is also important to reveal the reaction mechanisms of surface polar groups and H‐bond networks of natural polymer chains at different temperatures, electrolyte contents and current densities through in situ characterization, theoretical simulation and machine algorithm analysis. The associated research findings would provide a theoretical foundation for designing and constructing functional natural polymer‐based materials with stable electrochemical performance under lean‐electrolyte or high current densities. Meanwhile, constructing functionalized layered structures such as natural polymer‐based biomimetic SEI layers and biomimetic gel electrolytes by imitating biological systems is also considered to be an important approach for stabilizing zinc anodes under extreme conditions, thereby obtaining natural polymer‐based batteries that are resistant to extreme conditions.

### 4.9 Intelligent Degradation and Recycling to Enhance Sustainability

Green sustainability is also a crucial driving force for the industrial application of natural polymer‐based AZIBs. Future research should focus on developing natural polymer systems with environmental responsiveness, enabling controlled degradation, and recycling of these materials. The rich chemical structures and functional groups of natural polymers endow them with adaptive reaction capabilities under external stimuli. When specific electrochemical potentials, pH, temperatures, or light exposure can trigger the scission, rearrangement, or disassembly reactions of molecular chains, the controllable degradation of post‐use natural polymer‐based materials becomes achievable. Although this controllable degradation is attractive for end‐of‐life recycling, we need to keep an eye on the structural/chemical stability of these environmentally responsive natural polymers during long‐term electrochemical cycling. The developed natural polymers should strike a balance between degradability and stability, ensuring they don't undergo unexpected hydrolysis, oxidation, or structural decomposition in acidic or alkaline electrolytes or in the high‐voltage/strongly oxidative environment inside batteries. Future research is recommended to combine experimental and theoretical simulations to systematically reveal the reaction mechanisms of stimulus‐responsive chemical bonds and the influence laws of stimulus conditions on controllable degradation. In the meanwhile, it is also suggested to conduct in‐depth studies on dynamic covalent bonds, reversible cross‐linking networks, and ion‐induced depolymerization of oligomers, and summarize information such as the pH/temperature ranges and voltage windows that can stabilize these covalent bonds, cross‐linking networks, and oligomers, to guide the development of functional natural polymers that are suitable for AZIBs while balancing degradability and structural/chemical stability. These multifaceted endeavors are anticipated to achieve reversible, effective control over the degradation processes (defined as intelligent degradation) of natural polymer‐based materials with good electrochemical cycling stability, enabling the recovery and reuse of decomposition products. Furthermore, integrating such intelligently degradable natural polymer‐based materials with life cycle assessment can establish low‐carbon, closed‐loop manufacturing and recycling models, which is conducive to promoting the industrial application and sustainable development of natural polymer‐based AZIBs.

## Conflicts of Interest

The authors declare no conflicts of interest.

## Data Availability

The data that support the findings of this study are available from the corresponding author upon reasonable request.
